# Mapping the cause-specific premature mortality reveals large between-districts disparity in Belgium, 2003–2009

**DOI:** 10.1186/s13690-015-0060-5

**Published:** 2015-03-23

**Authors:** Françoise Renard, Jean Tafforeau, Patrick Deboosere

**Affiliations:** Department Public Health and Surveillance, Institute of Public Health, Brussels, Belgium; Interface Demography, Section Social Research, Free University of Brussels, Brussels, Belgium

## Abstract

**Background:**

Reducing premature mortality is a crucial public health objective. The goal of this paper, beside updating previous mortality atlases with recent data, is to explore the relative between-districts disparity using a relative-scale map design.

**Methods:**

Causes of deaths and population data were provided by Statistics Belgium. All deaths occurring in the periods 1993–1999 and 2003–2009, in people aged 1–74 residing in Belgium were included. Age-adjusted rates by cause of death were computed for both periods; the 2003-2009 rates were classified using a relative scale with a 10% change between each level and represented on chloropleths maps with a green-to-red divergent colour scheme centred on the national mean. This particular design allows the comparability across all the maps since a same meaning is given to a specific colour. The variation coefficient and the decile ratio were calculated and compared between the 2 periods.

**Results:**

A clear-cut regional divide at the disadvantage of Wallonia, and to some extent, of Brussels, was seen for all-cause, cardiovascular, cerebrovascular, diabetes, alcohol-related, mental and neurological diseases and non-transport accidents premature mortality. A NE-SW pattern parallel to the French border was observed for Lip, Oral Cavity, Pharynx, Larynx and oesophageal cancers, while various patterns are observed for the other cancers. The highest rates of road accident mortality are observed in the Southern districts. Many of those patterns are in continuity with earlier observations. However, the pattern of diabetes and mental and neurological diseases are new features. The variation coefficient and the decile ratios increased by 10% between the 1993-1997 and 2003-2009 periods.

**Conclusions:**

The use of a relative scale in the maps highlights the importance of the between-districts disparity in premature mortality, with for most causes, a clear-cut regional divide at the disadvantage of Wallonia; this health gap results of complex interactions between various factors, including the socioeconomic context, cultural habits and environmental influences, as well as public health policies. It should be considered as a warning signal, warranting the attention of the policy makers. Meanwhile, these results also highlight the potential for improvement in the more disadvantaged regions.

**Electronic supplementary material:**

The online version of this article (doi:10.1186/s13690-015-0060-5) contains supplementary material, which is available to authorized users.

## Background

Premature deaths are deaths that occur before a person reaches an expected age, ranging from 65 to 80 [1-4]. It is an important measure of a population’s health, as well as a tool to evaluate public health policies: indeed, reducing the premature mortality of the citizens is a crucial public health goal. In Belgium as in all industrialized countries, the premature mortality has decreased strongly over the past decennia; however, important regional differences exist, with higher premature mortality rates observed in Wallonia since the World War II [5,6], that have persisted up to now [7,8].

Chloropleths maps [9,10] - in which areas are shaded in proportion to the measurement of a statistical variable - provide an easy way to visualize how a measurement varies across geographical areas; they are well suited for the presentation and exploration of mortality rates. During the 70ths and 80ths, Belgium participated in an European project aiming at producing maps for some selected causes of premature mortality, the so called ‘avoidable’ causes [11,12]; in addition to those comparisons between the European broad regions, three successive Belgian atlases [13-15] produced maps at district level. Rates were classified according to sextile-classes. The Socio-economic survey (census 2001) has given rise to a new Belgian Atlas [16], covering multiple theme’s among which mortality and morbidity. This atlas mapped the overall and cause specific mortality in the period 1991–1995 at the district level: age-standardized mortality rates were classified according to a natural breaks classification [17], while a comparative mortality index was computed for broad age groups and represented in 4 classes (above or below the mean, statistically significant or not).

The latest published maps of premature mortality refer thus to years up to 1995; indeed, there has been a long hiatus in the publication of mortality data at Belgian level due to a reorganisation of the responsibilities in the death certificates processing. Consequently, for more than 12 years (1997 to 2009), Belgian causes of death data were only available for the Flemish and Brussels Regions. Recently, those data have been made available again at the national level. At the time of the analysis (December 2013), the data were available for nearly all years up to 2009 (except for 2000–2002). This prompted us to explore the recent geographical patterns of premature mortality at district level.

The goal of our study was not only to update the previous atlases with recent mortality data, but also to design the maps in a way that highlights the relative disparity between districts and maintain this relative scale comparable across all the maps. The heterogeneity between districts and its evolution over time has also been quantified by calculating summary disparity indices.

## Methods

**Data**: the individual causes of death and the population data aggregated by sex, year, age and districts were provided by Statistics Belgium, Directorate in charge of the publication of vital statistics at the national level. The causes of death are registered on the death certificates by a medical doctor; they are subsequently coded according to the ICD rules by trained staff within 2 regional Health Agencies (one for Flanders and Brussels, one for Wallonia), before being pooled at the national level by Statistics Belgium. Regular coordination meetings between the regional and federal levels are organised in order to guarantee consistency in the coding/registration rules. The causes of deaths data have been coded using the International Codification of diseases (ICD), using the 9^th^ version [18] until 1997 and the 10^th^ version [19] since 1998. Data of the years 1998 and 1999, turned out to be of poor quality and were not used in this work.

**Period**: the data of years 1993–1997 and 2003–2009 were pooled together. The 2003–2009 data were used to construct the maps, while the data of both periods were used to compute and compare the disparity indices.

**Age range:** in this work we defined premature mortality as deaths occurring before age 75; we also excluded infant deaths (before age of one year). **Calculation of rates**: 1–74 years age-adjusted rates were computed for both all-causes and main cause-specific premature mortality, by calculating the age-specific rates by 5 years age groups (4 years for the first group), and using the age distribution of the Belgian 2000 population in weighting. Confidence intervals around age-adjusted rates were calculated assuming a Poisson Distribution, and following the method described in Boyle [20]. Data were analysed according to the place of residence.

**Selection of the causes of death**: we produced maps for selected causes of death, based on two criteria: 1) the importance of the public health problem and 2) the number of deaths. Indeed, to avoid erratic rates due to small numbers in districts with few inhabitants and/or conditions with low mortality rates, maps were only produced for conditions with a sufficient number of cases in a sufficient number of districts. The threshold for a condition to be included was that the standard error should be less than 15% of the mortality rates in at least 75% of the districts. Because of too few cases, the maps of some specific causes were not produced for women.

Table 1 displays the list of conditions for which maps were produced, along with their ICD codes.

**Table 1:** **Causes of deaths selected to be shown on the maps**

**Cause of death**	**ICD-9 codes**	**ICD-10 codes**	**Age groups**	**Maps**	
				**Men**	**Women**
ALL CAUSES			All premature (1-74)	Figure 1a	Figure 1b
			40-59	*Additional file* *3*	*Additional file* *5*
			60-74	*Additional file* *4*	*Additional file* *6*
Cardiovascular diseases	390-398, 410-429, 440-459	I01-I09, I20-I52, I70-I99	All premature (1-74)	Figure 2	*Additional file* *7*
Cerebrovascular diseases & HTA	401-405,430-438	I10-I15,I60-I69	All premature (1-74)	*Additional file* *8*	*Additional file* *9*
Lung cancer	162	C34	All premature (1-74)	Figure 3a	Figure 3b
			40-59	*Additional file* *10*	*Additional file* *12*
			60-74	*Additional file* *11*	*Additional file* *13*
Lip, Oral cavity, Pharynx, Larynx and Oesophageal cancer	140-149, 150, 161	C00-C14, C15, C32	All premature (1-74)	Figure 4	-
Colorectal cancer	153-154.0,154.1	C18-C20	All premature (1-74)	*Additional file* *14*	*Additional file* *15*
Breast cancer	174	C50	All premature (1-74)	-	*Additional file* *16*
					*Additional file* *17*
					*Additional file* *18*
Diabetes mellitus (anal.in multiple causes)	250 (No multiple causes analysis)	E10-E14	All premature (1-74)	Figure 5	*Additional file* *19*
Mental and neurological Diseases excluding alcohol-related deaths	290-319, 320-389 (except 291)	F and G , except F10,G312,G621	All premature (1-74)	*Additional file* *20*	*Additional file* *21*
COPD	490-492, 496	J40-J44	All premature (1-74)	*Additional file* *22*	*-*
Alcohol-related death (an.in mult.causes)	291, 303, 860, 305.0, 425.5, 535.3, 5710:571.3,571.5 (No multiple causes analysis)	F10, G312, G621, I426, K292, K860, K852, K70, K73, K74 (exc. K74.3 K74.4 K74.5), X45	All premature (1-74)	Figure 6	*Additional file* *23*
Road accidents	810-829	V00-V89 (except V81, V82)	All premature (1-74)	Figure 7	-
Non-transport accidents	850-869, 880-915	W00-X59	All premature (1-74)	*Additional file* *24*	-
Suicide	950-959	X60-X84	All premature (1-74)	*Additional file* *25*	-

Some causes of deaths that could present certification/coding regional biases were pooled together: it was the case for cerebrovascular diseases and hypertension [11], ischemic heart diseases and other heart diseases (pooled into a category ‘Cardiovascular Diseases’), and finally mental and neurological diseases that were reported together because of regional differences in the coding of dementia. Alcohol-related deaths list is based on ICD10 definition. To avoid redundancy in our classification, we constructed a class ‘Mental and neurological diseases excluding alcohol-related deaths’.

Multiple-causes analysis has been performed for diabetes and alcohol related mortality, verifying the presence of the specific condition in the underlying as well as in the immediate, intermediate and associated causes of death. This method is more and more recommended in the study of several causes of death [21].

### Construction of the maps

The age-adjusted premature mortality rates by district during the period 2003–2009, for all causes and by main specific causes were mapped (see list of the maps in Table 1). When the number of cases was large enough, we have also produced maps for the specific 40–59 and 60–74 age groups.

**Figure 1:**
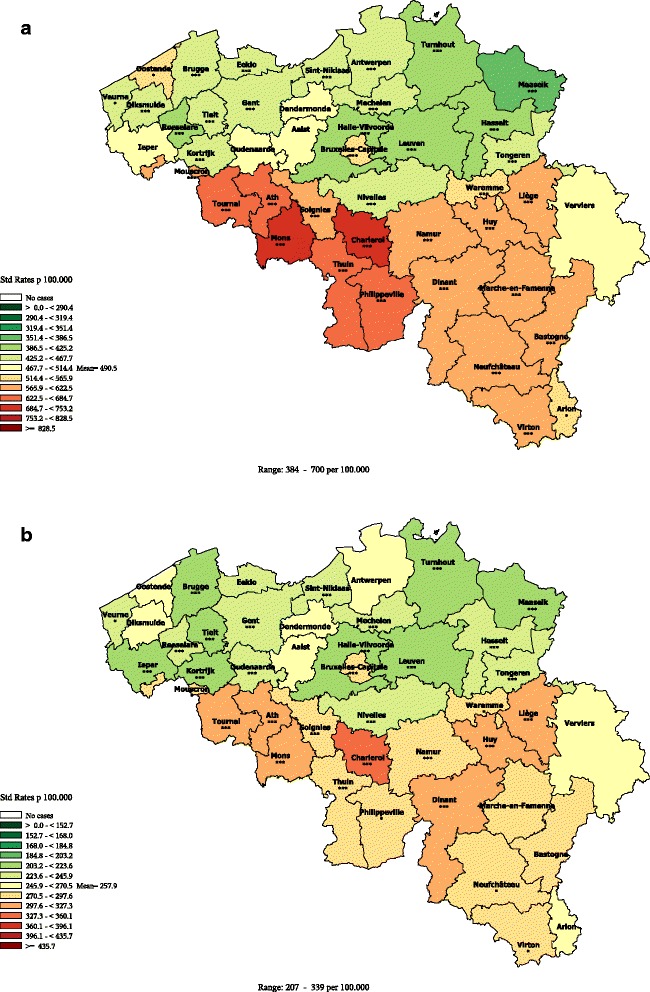
**All causes premature mortality (1–74 years) by district, Belgium 2003–2009.** **a**: Men **b**: Women.

Design: chloropleths maps with a diverging scale of 13 colours varying from green- for low rates- to brown - for high rates were produced. The cut-off colour (yellow) represents the reference rate, which is for all maps the Belgian sex-specific mortality rate for a particular cause of death. This reference colour has the same meaning (average sex-specific level) across all maps, allowing to easily compare the maps. As the main purpose was to represent relative disparity, rates were classified according to a geometric progression. A 1.1 step between the classes was chosen, meaning that each next threshold of a colour level is 1.1 time higher than the previous one [22]. The relative distance between the highest and the lowest classes is 1.1 ^[number of colour classes-1]^. Therefore, the larger the between-district disparity, the more colour classes will be present and the sharper the colour contrast will be. For instance, the map of all cause premature mortality in males (1–74 years) has 8 colour classes (Figure 1a), while the map of females (Figure 1b) only contains 6, because the disparity between the extreme rates is sharper in men (RR = 1.9) than in women (RR = 1.6).

The legend of the maps displays the boundaries of each class expressed in rates. The midpoint of each class is calculated as the average rate * (1.1) ^rank of the class, starting from the average class^. So, for the premature mortality in males, the highest class of rates, which is observed in Mons and Charleroi, ranks at the 4^th^ position above the average. The midpoint of this class is 490.6*(1.1)^4^ = 718 per 100.000 (boundaries 684.3-753.7). The lowest rate is observed in Maaseik (3^rd^ class below the average). The midpoint of this class is 490.6*(1.1)^−3^ = 368.5 per 100.000 (boundaries: 351.4-386.5).

The spatial patterns of each map were visually explored and described. In addition to the maps, tables display the district specific rates ranked in ascending order, along with their confidence intervals, and the p value of a z statistic testing for a difference between the district rate and the mean rates [23] (see tables in Appendix).

### Dispersion measures

For both periods (1993–1997 and 2003–2009), the following statistics were computed:

- The variation coefficient, as the standard error of the adjusted rates divided by the average adjusted rate for a specific condition and by sex.
- The decile ratio (p90/p10) of the adjusted rates of deceased people. Decile ratios [24] stress the gap between the top and the bottom of a distribution. It is calculated as the number of deaths observed in the lowest 10% of the population (after having ranked the districts according to their mortality rates) divided by the number of deaths observed in the upper 10% of the population [25]. The ratios were presented with their confidence interval. The evolution of the decile ratios over time was measured and tested with a z test according to Altman’s method [26].

## Results

### Maps

To facilitate the reading of the text in the results section, administrative maps of Belgium are provided here in Additional files 1 and 2.

### All causes mortality

The maps of all causes premature mortality (Figure 1) show a clear regional divide with lower than average rates in Flanders (at the exception of the district of Ostend) than in Wallonia (except Nivelles) and Brussels, as well in men as in women. The Northeast districts show slightly better rates than the rest of Flanders. The districts situated in the province of Hainaut (Tournai, Ath, Mons, Soignies, Charleroi, Thuin, Philippeville) show higher rates than in the rest of Wallonia. Hot spots are seen respectively in the districts of Mons and Charleroi for men, and the district of Charleroi for women. Age-specific maps show higher disparity in younger (40–59 year) than in older (60–74 year) men (see Additional files 3, 4, 5, and 6).

The spatial pattern observed in all causes mortality reflects the result of the various cause-specific mortality spatial distributions.

### Cause-specific premature mortality

The next maps are focusing on the spatial distribution of those specific causes of death that were selected. Some maps were not displayed for women because of too few cases. Different patterns in the cause specific mortality spatial distribution could be identified.

### Natural causes

#### Cardiovascular diseases premature mortality

**Figure 2:**
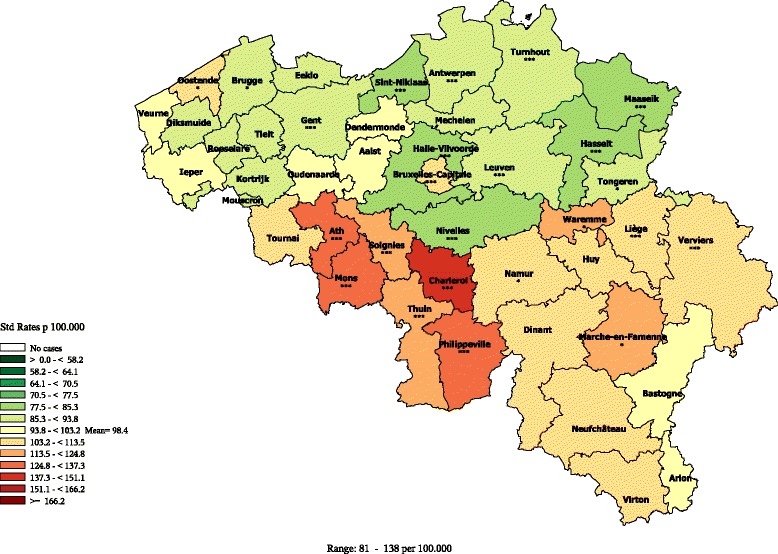
**Cardiovascular diseases premature mortality by district in men (1–74 year), Belgium 2003–2009.**

In men, the cardiovascular (CVD) premature mortality exhibits a clear N-S divide (Figure 2), with a moderately high disparity. In Wallonia, most districts exhibit a higher than average rate (at the notable exception of Nivelles where a better than average rate is observed). The highest rates are observed in the districts of Hainaut (mainly in Charleroi, Philippeville, Mons and Ath). At the contrary, in Flanders, most districts exhibits lower than average rates, with a few districts from the West and East Flanders provinces being at the average Belgian level (except Ostend). The rate in Brussels is slightly higher than the Belgian average. The patterns in female mortality are quite similar as those observed for males with slighter regional disparities (see Additional file 7).

#### Cerebrovascular and hypertensive diseases premature mortality

In men, the global pattern of Cerebrovascular and Hypertensive Diseases premature mortality is similar to the one of Cardiovascular Diseases, with a more pronounced excess in the Hainaut province. Also the districts of the province Antwerp (Sint-Niklaas, Antwerp, Mechelen) show an average level of mortality, contrasting with the favourable Flemish rates. The rate in Brussels is slightly above the average as well (Additional file 8).

In women, the geographical pattern is less clear, with several Flemish districts above the average rate and some Walloon districts below the average rate (Additional file 9).

#### Lung cancer premature mortality

**Figure 3:**
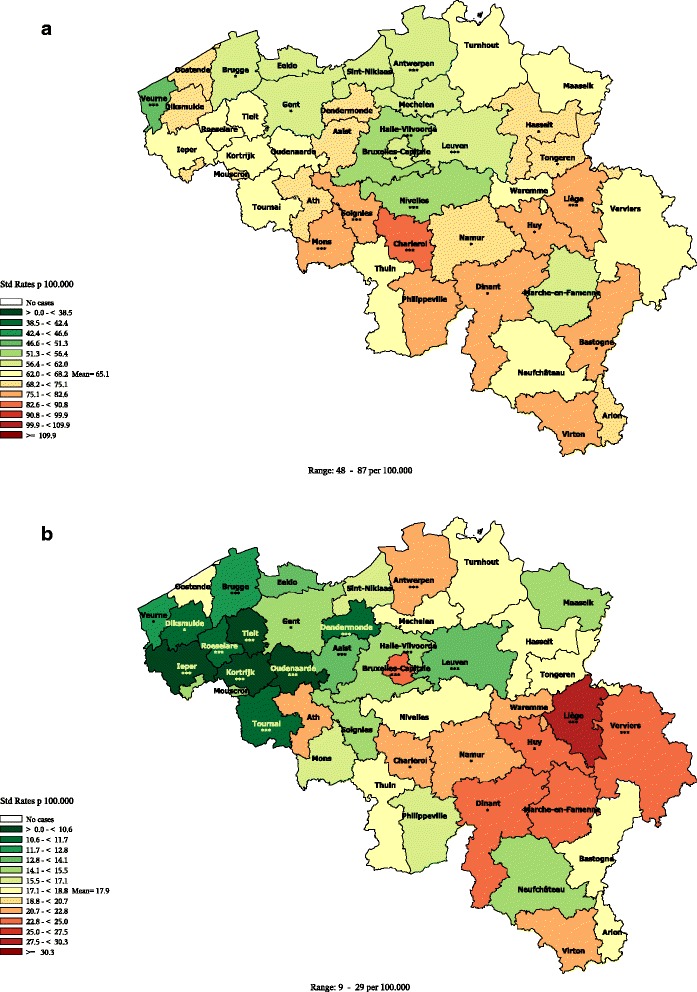
**Lung cancer premature mortality (1–74 year) by district, Belgium 2003–2009.** **a**: Men **b**: Women.

In men, the premature mortality due to lung cancer does not exhibit a strong geographical pattern, and the disparities are moderate (Figure 3). In Flanders, lower than average rates are observed in all the districts of the provinces Flemish Brabant and Antwerp, and in the districts of Gent, Eeklo, Brugge and Veurne. In the other Flemish districts, the rates are close to the average or just above. In Wallonia, the rates are low only in the districts of Nivelles and Marche, being close or above to the average in the rest. The highest rates are observed in some districts of the provinces Hainaut (mostly Charleroi), Liège, Luxemburg and Namur. As the other central districts, Brussels exhibits a low rate.

When looking at the age-specific maps (Additional files 10 and 11), we observe a clear N-S divide in the age group 40–59 exhibits, while no clear pattern is seen in the age-group 60–74.

In women, the distribution of the lung cancer premature mortality exhibits a E-W gradient, with higher rates in South-East than in the North-East, and also higher rates in the big agglomerations (Brussels, Antwerp, Liège). The rates are very low in the west part of Belgium (as well in Wallonia as in Flanders).

As observed in men, the women lung cancer mortality in the age group 40–59 is higher in the South of Belgium (see Additional files 12 and 13).

#### Lip, oral cavity, pharynx,larynx and oesophagus cancers premature mortality in men

**Figure 4:**
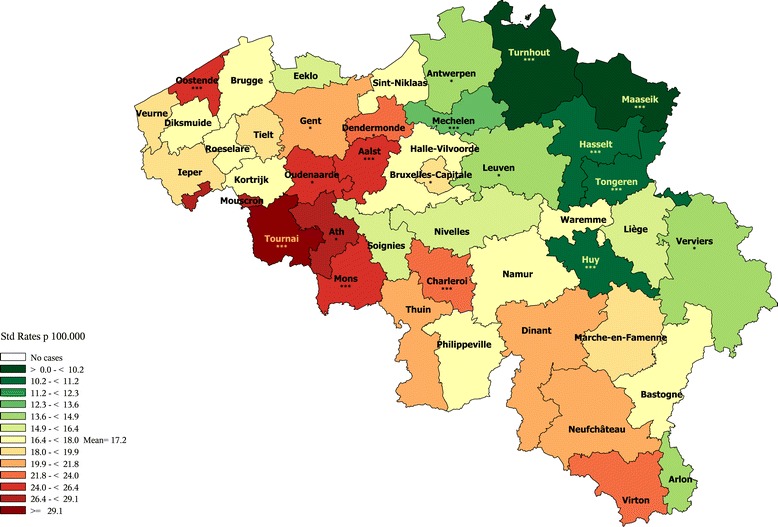
**Lip, oral cavity, pharynx, larynx & oesophagus cancers premature mortality by district in men (1–74 years), Belgium 2003–2009.**

The premature mortality from Lip, Oral cavity, Pharynx, Larynx and Oesophagus cancers (Figure 4) follows a clear gradient parallel to the French border (maximal along a NW-SW axis, decreases when moving to East). This distribution has also been described for the incidence of those cancers [27,28].

#### Colorectal cancer premature mortality

The distribution of the colorectal cancer premature mortality in men does not exhibit a clear geographical pattern (Additional file 14). Most of the districts present close to average rates. Lower rates are observed in the East and Northwest. No clear pattern is observed in women neither (Additional file 15).

#### Breast cancer premature mortality in women

The breast cancer premature mortality in women shows a very slight West–east gradient (Additional file 16); most of the East side districts have below average rates while most of the West side districts –including Brussels- present average or slightly higher than average rates (excepted Veurne) with rates significantly above the average in Dendermonde and Kortrijk. When looking at the age-specific rates, no systematic pattern is observed in women aged 40–59 (Additional file 17), while a clear W-E gradient is seen in the age group 60–74 years (Additional file 18).

#### Diabetes mellitus premature mortality

**Figure 5:**
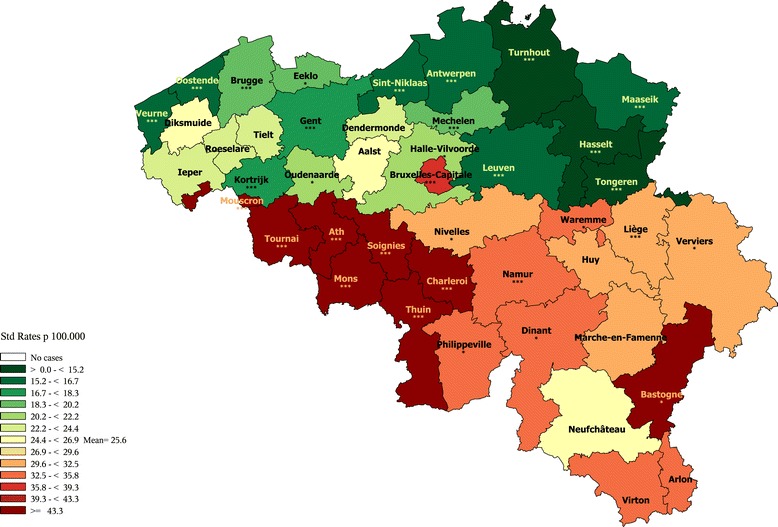
**Diabetes mellitus premature mortality by district in men (1–74 years), Belgium 2003–2009.**

The diabetes premature mortality, analysed in multiple causes**,** presents a sharp regional divide for both sexes. In men (Figure 5), all districts of Wallonia are above the average (except Neufchateau with an average rate); all rates in the Hainaut districts pertain to the highest category; the Brussels rate is also quite high. The Flemish rates are all below the average (except Diksmuide and Aalst with average rates) with the lowest rates in the Limburg province and some districts of the West Flanders. In women, a similar distribution is observed (Additional file 19), except for the districts of Diksmuide (Flemish district with a higher than average rate), Nivelles, Neufchateau and Arlon (Walloon districts with a lower than average rate).

The same distribution was observed when looking only at diabetes premature mortality in the underlying cause (data not shown), except for the female rate in Brussels which was below the average.

#### Mental and neurological diseases premature mortality

The mental and neurological diseases premature mortality (excluding alcohol related deaths) also exhibits a regional pattern with higher rates in Wallonia (except in Nivelles, with medium rates in both sexes, Dinant and Soignies, with medium rates in women, and Marche with a low rate in women) and lower rates in Flanders (except in Aalst with a medium rate). Brussels presents an average rate in both sexes (Additional files 20 and 21).

##### Chronic Obstructive Pulmonary Diseases (COPD) premature mortality in men

The chronic obstructive diseases premature mortality also exhibits a North–south regional pattern; moreover, in Wallonia, hot spots are observed in Mons, Charleroi, Philippeville and Liège. A slightly above-average rate is observed in Brussels (Additional file 22).

#### Alcohol-related premature mortality

**Figure 6:**
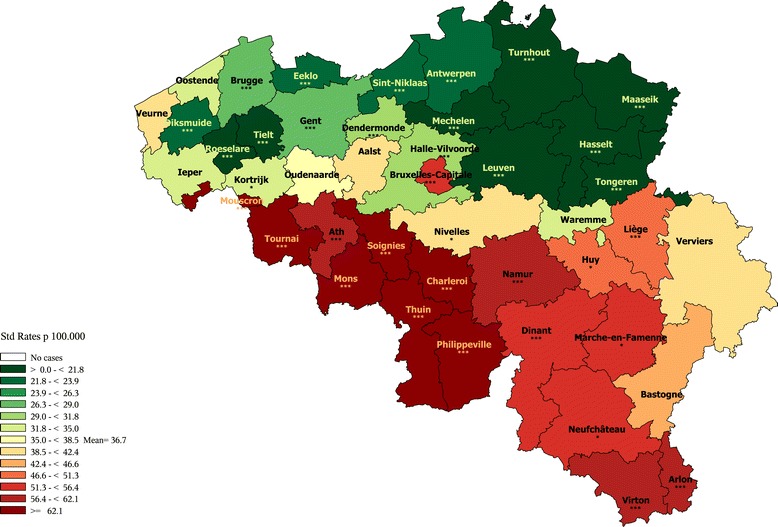
**Alcohol-related premature mortality by district in men (1–74 years), Belgium 2003–2009.**

The alcohol-related premature mortality (Figure 6 and Additional file 23) also exhibits a clear-cut regional divide with higher rates in Wallonia (except in Nivelles, with medium rates in both sexes, Dinant and Soignies, with medium rates in women) and lower rates in Flanders (except in Aalst with a medium rate). Brussels presents higher than average rate in both sexes. A West–east decreasing gradient is present as well.

### External causes

#### Road accident premature mortality in men

**Figure 7:**
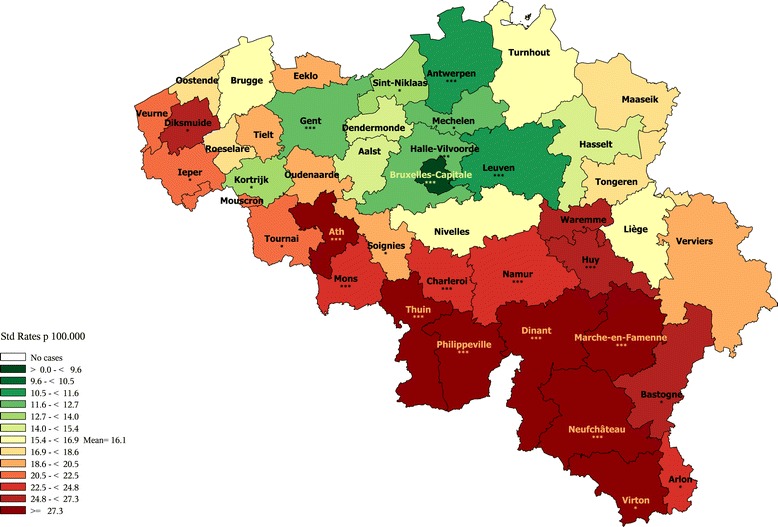
**Road accidents premature mortality by district in men (1–74 years).**

Road accident premature mortality in men (Figure 7) is higher along the French border and in all Wallonia (excepted Liege and Nivelles), where it is particularly high in the South, and in 2 districts of the Limburg (Maaseik and Tongeren). It is very low in Brussels. In Flanders, particularly low rates are observed in the Flemish Brabant and Antwerp provinces, and in Ghent.

#### Non-transport accident premature mortality in men

The geographical pattern of non-transport accident premature mortality in men (Additional file 24) follows again a South–north regional divide (except for the district of Ostend that has a high rate). The Brussels rate is higher than average as well.

#### Suicide premature mortality in men

Low rates of suicide premature mortality (Additional file 25) in men are observed in the Northeast of Belgium (Antwerp, Limburg and Flemish Brabant provinces) and in Brussels. Hot spots are observed in the East of Wallonia (except along the German border) and in Ath and Tournai; in Flanders, the highest rate is observed in Eeklo, with moderately high rates in some districts of West and East Flanders.

### Dispersion measures

Table 2 displays two dispersion measures for each cause of death, for the periods 1993–1997 and 2003–2009: the decile ratio, which expresses the ratio between the lowest and highest deciles, and the variation coefficient, which measures the global disparity between all the districts rates and the mean rate.

**Table 2:** **Evolution of the between-districts disparity by main causes of premature mortality**, **Belgium, periods 1993–1997 and 2003-2009**

**Sex = males**								
***Cause of death***	***Mean rate 1993-1997***	***Mean rate 2003-2009***	***Coeff.Var. 1993-1997***	***Coeff.Var. 2003-2009***	***Decile ratio [CI] 1993-1997***	***Decile ratio [CI] 2003-2009***	***Change dec.ratio***	***p value***
ALL CAUSES	663.6	515.7	15%	18%	1.56 [1.5; 1.6]	1.71 [1.7; 1.7]	9.7%	<0.001
Lip, Oral cav., Pharybx, Larynx & Oesoph. Ca	22.1	18.2	34%	27%	3.19 [2.8; 3.7]	2.56 [2.3; 2.9]	−19.8%	<0.05
Lung Ca	95.6	67.0	13%	12%	1.46 [1.4; 1.5]	1.47 [1.4; 1.6]	0.2%	ns.
Diabetes Mel,multiple causes	.	28.4	.	41%		3.41 [3.1; 3.8]	.	
Diabetes Mel. (underl.cause)	7.6	7.5	36%	50%	3.19 [2.5; 4.0]	3.78 [3.1; 4.6]	18.5%	ns.
Mental & Neurol. DIS	20.7	23.1	24%	34%	2.07 [1.8; 2.3]	2.67 [2.4; 3.0]	29.5%	<0.01
Cerebrov. dis. & HTA	35.5	24.1	16%	22%	1.56 [1.4; 1.7]	1.91 [1.7; 2.1]	22.1%	<0.01
Cardio-Vasc. Dis	155.4	101.5	14%	14%	1.52 [1.5; 1.6]	1.56 [1.5; 1.6]	2.8%	ns.
Chron. Obs. Pulm. Dis.	35.7	23.5	29%	28%	2.20 [2.0; 2.4]	2.34 [2.1; 2.6]	6.1%	ns.
Alcohol-rel_dt (Mult.causes)	.	40.6	.	46%		5.60 [5.1; 6.2]	.	
Alcohol-rel dt (underl.cause)	21.0	19.8	49%	48%	7.52 [6.2; 9.1]	5.64 [4.9; 6.5]	−25.0%	<0.05
EXTERNAL CAUSES	83.1	72.0	25%	26%	1.93 [1.8; 2.1]	2.00 [1.9; 2.1]	4.1%	ns.
Road accident	29.1	20.9	35%	38%	2.70 [2.4; 3.0]	3.42 [3.0; 3.9]	27.0%	<0.01
Non transp.acc. (Pois/fall/envir.)	15.3	17.0	30%	32%	2.60 [2.2; 3.0]	2.45 [2.2; 2.8]	−5.5%	ns.
Suicide	31.9	29.8	25%	24%	2.14 [1.9; 2.4]	1.83 [1.7; 2.0]	−14.8%	<0.05
***Sex = females***								
***Cause of death***	***Mean rate 1993-1997***	***Mean rate 2003-2009***	***Coeff.Var. 1993-1997***	***Coeff.Var. 2003-2009***	***Decile ratio [CI] 1993-1997***	***Decile ratio [CI] 2003-2009***	***Change dec.ratio***	***p value***
ALL CAUSES	315.7	261.1	11%	14%	1.40 [1.4; 1.4]	1.54 [1.5; 1.6]	9.8%	<0.001
Lip, Oral cav., Pharybx, Larynx & Oesoph. Ca	3.6	3.8	46%	33%	4.74 [3.2; 6.9]	2.54 [2.0; 3.3]	−46.3%	<0.01
Lung Ca	12.4	16.8	33%	28%	2.69 [2.3; 3.2]	2.52 [2.2; 2.8]	−6.3%	ns.
Breast Ca	34.2	26.1	14%	13%	1.48 [1.3; 1.6]	1.45 [1.3; 1.6]	−1.8%	ns.
Diabetes Mel, multiple causes	.	15.8	.	35%		2.55 [2.3; 2.9]	.	
Diabetes Mel. (underl.cause)	6.6	4.4	33%	38%	2.41 [1.9; 3.1]	2.93 [2.3; 3.7]	21.2%	ns.
Mental & Neurol. DIS	13.6	14.5	23%	31%	1.99 [1.7; 2.3]	2.16 [1.9; 2.4]	8.5%	ns.
Cerebrov. dis. & HTA	23.2	15.7	18%	18%	1.72 [1.5; 1.9]	1.72 [1.5; 1.9]	0.5%	ns.
Cardio-Vasc. Dis	60.9	39.2	15%	18%	1.54 [1.4; 1.7]	1.79 [1.7; 1.9]	15.9%	<0.01
Chron. Obs. Pulm. Dis.	7.2	7.9	39%	49%	3.23 [2.6; 4.1]	4.00 [3.4; 4.8]	23.9%	ns.
Alcohol-rel_dt (Mult.causes)	.	15.2	.	49%		6.06 [5.1; 7.2]	.	
Alcohol-rel dt (underl.cause)	9.4	8.5	58%	53%	7.24 [5.6; 9.4]	7.39 [5.8; 9.4]	2.2%	ns.
EXTERNAL CAUSES	30.8	25.4	21%	22%	1.86 [1.7; 2.1]	1.91 [1.7; 2.1]	2.5%	ns.
Road accident	9.3	6.0	38%	45%	2.68 [2.2; 3.3]	3.65 [2.9; 4.6]	36.0%	ns.
Non transp.acc. (Pois/fall/envir.)	6.7	7.0	28%	40%	2.59 [2.0; 3.3]	3.25 [2.7; 3.9]	25.5%	ns.
Suicide	11.1	10.1	25%	22%	2.06 [1.7; 2.4]	2.02 [1.7; 2.3]	−1.9%	ns.

The decile ratio (d90/d10) is 1.75 in men and 1.54 in women (Table 2) during the period 2003–2009, indicating a moderate to high dispersion.

In men, high decile ratios (> = 3) were observed, in both periods for alcohol-related, diabetes and road accident mortality, with also a high variation coefficient (38-45%). Moderate (1.5-3) decile ratios were observed for cardiovascular, cerebrovascular and suicide mortality, with variation coefficient ranging from 14% to 34%. A low decile ratio (<1.5) was observed for lung cancer with a variation coefficient of 12%. In women, high decile ratios (> = 3) were observed for alcohol-related, diabetes, chronic obstructive pulmonary diseases, road accident and non-transport accident mortality, with variation coefficients higher than 40%. Moderate decile ratios (1.5-3) and variation coefficient (18-38%) were observed for Lung cancer, Lip, oral cavity, pharynx, larynx & oesophagus cancers, diabetes mellitus, mental and neurological diseases, cardiovascular and cerebrovascular and suicide mortality. A low (<1.5) decile ratio was observed for breast cancer, with a 13% variation coefficient.

The decile ratio is significantly higher in men than in women for diabetes and mental and neurological diseases and significantly higher in women than in men for lung cancer and cardiovascular disease.

Both the decile ratio and the variation coefficient show an increased disparity between the 2 periods: as compared to the period 1993–1997, the 2003–2009 decile ratio increased by 10% in both sexes. The variation coefficient increased from 15% (1993–1997) to 18% (2003–2009) and from 11% (1993–1997) to 14% (2003–2009) respectively in men and women Qua evolution of the cause-specific disparity between the 2 periods, a significant increase of the decile ratio was observed in men for all-causes, cerebrovascular, mental health and road accident mortality, with a variation coefficient evolving the same way. The disparity in alcohol-related and suicide mortality decreased significantly, but the variation coefficient remained stable, meaning a discrepancy between the disparity between the lowest and highest levels and the global disparity in the whole districts distribution.

In women, a significant decrease of the decile ratio between the 2 periods was observed for Lip, Oral Cavity, Pharynx, Larynx and Oesophagus cancers mortality, with also a decrease in the variation coefficient; an increase in the disparity was observed for the cardiovascular mortality.

## Discussion

This study updates, for the period 2003–2009, previous works on geographical disparity of the premature mortality by specific causes in Belgium. At the same time, the maps have been designed with a classification scheme focusing on the relative between-districts disparity. This type of scale allows comparing in a glance the importance of the disparity across the various specific causes of deaths for each sex. We think this scaling principle provides added value compared to the commonly used percentiles classification [29,30], where always regions with lowest and highest rates are coloured with the extreme colours, no matter how large or small the variation is.

### Coding and quality of data issues

The production of cause-of-death statistics relies on two steps, both of them being crucial for the data quality. First, the certifying physician reports the chain of events leading to death on the certificate (certification step). Second, this information is coded (codification step) following guidelines published in the International Classification of Diseases (ICD9 and ICD10) [31]. In Belgium, the codification step is carried out by two regional Health Agencies (the first one coding for the Flemish and Brussels regions, the second one coding for Wallonia).

Aelvoet [32] has investigated the coding procedure in Belgium in the 90th; he concluded that the quality of death certificates had substantially improved since 1993 due to a more centralized organisation. However, he recommends caution in analysing the oldest age groups. By restricting the recent analysis to the premature mortality (1–74 years), this study thus avoids the more questionable age groups.

Regional differences in certifying/coding practices can lead to comparability biases. In Belgium, no systematic analyse of those differences in practices has ever been performed. For some causes, regional differences in the certifying/coding practices can be suspected, for instance when mirror rates are observed between the regions for codes assigned to close conditions. This is the case for dementia, with much higher rates of organic dementia (coded F0.0, F03) in Flanders than in Wallonia, while the reverse is observed for Alzheimer dementia (coded G30) with higher rates in Wallonia. However, without supplemental surveys with an appropriate methodology (for instance with a cross-coding), it is impossible to quantify precisely the extent to which certification and coding practices are involved in regional mortality disparities.

To minimize the risk of regional comparability bias, those conditions in which varying coding habits have been reported in the literature or observed in Belgium have been pooled into broader categories: for instance the ‘Ischemic Heart Diseases’ and the ‘Other Cardiovascular Diseases’ were pooled into a category ‘Cardiovascular Diseases’, the Cerebrovascular Diseases’ were pooled with the ‘Hypertensive Diseases’, and the ‘Mental Diseases’ were pooled with the ‘Neurological Diseases’.

Official statistics on the causes of death are limited to the underlying cause; however, a multiple-causes analysis (which is an analysis of a specific condition across all the causes mentioned in the death certificates: underlying, immediate, intermediate or associated) is an interesting additional approach [21,33]. It presents a double advantage: first, it gives a better estimate of the burden of the disease. Second, it reduces comparability problems linked to the choice of the underlying cause when several causes are mentioned. This approach has often been used for diabetes [21,34], alcohol-related deaths [35] and COPD [36]. In the present study, a same sharp regional divide was observed for alcohol-related and diabetes premature mortality when analysed by underlying or multiple causes. This made us confident that the observed pattern was not due to a difference in the way of selecting the underlying cause.

**Timeliness of the Belgian mortality data:** we used the more recent data available in 2013, that is data of year 2009; although this still represented a 4 years backlog, this was a real improvement as compared to the situation that prevailed before, where the publication of mortality data at Belgian level had stopped during about 12 years, due both to the reorganization of the registration and to difficulties in one of the regions when upgrading the registration according to the 10^th^ version of the ICD. This backlog has now still reduced to no more than 2–3 years. A gap persist for the years 2000–2002 that should also be fulfilled during calendar year 2015.

**Age limits:** studies of premature deaths use various upper age cut-off. This upper cut-off used to be set at 65 in the last century, then gradually increased as life expectancy lengthened. After the year 2000, upper cut-offs are more commonly situated between 70 to 80 years [1,4,37,38]. In the current study the upper cut-off was set at 75 years for two reasons: reported conditions for deaths occurring after 75 years are generally less reliable because of more frequent competing causes of death in older people; moreover the choice of a 75 year upper- limit is consistent with the recent definition of avoidable mortality [39].

Infant deaths have been excluded from the present study, because infant mortality is due to causes that are very specific to this age group. Also, more appropriate indicators exist that specifically address death in this particular age group.

### Summary and interpretation of the main findings

#### All cause premature mortality

The health gap -represented by the differences in all causes premature mortality rates - between the Belgian districts, reflects a clear regional divide; this is true for all-cause as well as for many (but not all) specific causes premature mortality. The between-district disparity in all cause premature mortality has increased by 10% between the periods 1993–1997 and 2003–2009, as well in males as in females. In our previous work [8], we had studied the ratios of the age-adjusted rates at regional rather than at district level, and arrived to a similar conclusion: the Wallonia/Flanders rate ratio increased from 1.30 to 1.37 in males and from 1.21 to 1.29 in females respectively for the periods 1993–1997 and 2003–2009. The health gap between the regions in Belgium is large and has increased during the last decennia in both sexes.

The level of the mortality rates is the result of a complex interaction of many factors, including genetic, behavioural and environmental features as well as the health system performance. Those factors are themselves shaped by the individual socio-economic status, the global socio-economic context, the culture and the health-oriented public policies.

The EU-SILC survey 2011 (http://ec.europa.eu/eurostat/web/income-and-living-conditions/overview) stresses the socio economic differences between the regions, with a much better situation in Flanders than in Wallonia qua unemployment, poverty risk and deprivation [40,41]. The most worrying situation is described in Brussels, for all poverty and precarity indicators. Among the Walloon districts, the district of Nivelles occupies a privileged position, ranking 3^rd^ of Belgium for the wealth index (http://statbel.fgov.be/fr/modules/publications/statistiques/marche_du_travail_et_conditions_de_vie/Statistique_fiscale_des_revenus.jsp).

There is no doubt that this worse socio-economic context explains a large part of the current Walloon excess in all-cause premature mortality, both directly and by influencing a whole range of risk factors. However, the regional inversion in mortality appeared in 1947, before the economic decline of the Wallonia, suggesting that other factors than the macro-economic context are also involved in the regional difference [42]. Cultural habits (for instance nutritional) and occupational conditions are important. Environmental pollution probably has a role also, but the study of the distribution of the various health-affecting pollutants was out of the scope of this analysis. Moreover, many public health policies (namely policies related to health promotion and prevention) have been regionalized and this can have resulted in different health outcomes by region.

#### Interpretation of the cause-specific premature mortality patterns

North – South

A clear North–south divide with locally some hot-spots or spared districts was observed for a large number of causes. For most of those causes, the district of Nivelles performed better than the rest of Wallonia. Brussels generally exhibits an intermediate rate between Flanders and Wallonia. This N-S divide was observed for:

- Cardiovascular diseases in both sexes
- Cerebrovascular diseases in men.
- Diabetes in both sexes
- Mental and neurological Diseases in both sexes
- Chronic Obstructive Pulmonary Diseases in men (not mapped for women because of a too low number of cases)
- Alcohol-related deaths (excluding cancer-related and external causes) in men (not mapped for women).
- Non-Transport accident in men (not mapped for women)

The classical risk factors for **Cardiovascular Diseases** include tobacco use, obesity/overweight, excessive consumption of animal fat, lack of physical activity and stress. A low socioeconomic position has shown to be important risk factors of CVD mortality as well. Recent studies also stressed a link of cardiovascular mortality with fine particles pollution [43,44]. The cardiovascular mortality has strongly decreased in Belgium since the 70^th^ [16] as well as in most industrialized countries; such an improvement can be attributed to public health efforts in the prevention of the risk factors by means of the primary prevention (promotion of healthy lifestyles), secondary prevention (via the reduction of the blood pressure and the cholesterol level) as well as medical/surgical technology. Despite this favourable evolution at country level, the well-known regional disparities in cardiovascular premature mortality at the disadvantage of the Walloon Region persist. Both the distribution of the behavioural risk factors and socio economic features in Belgium can contribute to explain this pattern. Indeed, the Health Interview Surveys, realised in 1997,2001,2004 and 2008, (https://his.wiv-isp.be/SitePages/Home.aspx) reveal, for most lifestyle, healthier habits in Flanders than in Wallonia [45]: higher fruits and vegetable consumption, less animal fat consumption, higher rate of physical activity, lower tobacco use (except for women in the province Antwerp); also the prevalence of obesity is higher in Wallonia [46]. The **Cerebrovascular Diseases** share to a large extend the same risk factors as cardiovascular disease, because the main patho-physiological mechanism of both diseases is atherosclerosis; not surprisingly, the patterns of the cardiovascular and cerebrovascular diseases mortality are quite similar.

The very sharp regional difference in the **Diabetes** premature mortality had not been described in previous studies. It is at least partially due to a difference in the obesity prevalence, which has been documented in all the waves of the Health Interview Surveys. Other causes could include a difference in the accessibility/quality of care and in the treatment compliance.

**Alcohol-related deaths** are known to have a strong social gradient and the N-S divide, already present in previous studies, is most probably explained by the more disadvantaged socio-economic context in Wallonia.

The **COPD** premature mortality has strongly declined since the beginning of the 90ths [8]. However, it has declined faster in Flanders than in Wallonia. Indeed, the 1993–1997 distribution highlighted hot spots in the Eastern Flanders that have all disappeared. Main risk factors for COPD are tobacco smoking, environmental air pollution and occupational exposure to dusts and chemicals [36]. The current pattern could reflect a residual excess of COPD mortality due to industrial pollution, former occupational exposure to coal dust (coalfield area), and higher tobacco use.

The regional difference in **Mental and Neurological Diseases** premature mortality (studied after exclusion of the alcohol-related causes) is also appealing, and was never described before. Those two chapters of the ICD classification group a set of various diseases; there is no straightforward explanation for this complex matter. This issue definitely deserves more research.

The category **Non-transport Accidents** groups various events like falls, poisoning, drowning; it would be worthwhile to further explore the subcategories to attempt finding an explanation for the observed differences. However, the coding of external causes has become very difficult in the ICD10 classification, and a detailed analysis might suffer from coding habits differences at regional level.

NorthWest - SouthEast

A NW-SE gradient (parallel to the French boundary and diminishing to the NE direction) is observed for:

- Head and Neck, Oesophagus and Liver Cancers (in men)
- Suicide (in men)

The major risk factor for developing **Lip,Oral cavity, Pharynx, Larynx & oesophagus cancers** is the conjunction of alcohol and tobacco consumption. Very high incidence rates were formerly observed in France [47], with a progressive decrease during the last decennia [48]; it is possible that some patterns of alcohol and tobacco consumption (frequency, products) were shared across the border and could explain the typical NW-SE gradient observed in incidence as well as in mortality. Henau et all [28] describe a same NW-SE gradient for **Lip, Oral cavity, Pharynx, Larynx & oesophagus cancers** incidence as the one observed for their mortality. They also describe an identical NW-SE geographical pattern of chronic liver diseases mortality, considered as a proxy of alcohol consumption; however, the all alcohol-related causes pattern seems to present also a North–south divide, with a superimposition of a W-E gradient. This point should be further explored in order to propose prevention strategies.

**Suicide** is known to be underreported, with a degree varying from country to country, and mainly related to cultural and legal issues [49-51]. The reporting of suicide is the responsibility of the certifying doctor; it is not excluded that some of the variation can be due to variation in certification habits. In particular in Brussels, it is known that many external causes of with undetermined intend are waiting for the result of an investigation (Flemish Office of registration of the causes of deaths, personal communication). We have looked at the suicide repartition after applying the Jougla’s correction [50]: the variation remained quite the same, except in Brussels where the rates moved towards one colour class higher.

Varying patterns

Various other patterns were observed for:

- Breast Cancer in women: slightly higher rates are observed in the Western part of the country.
- Lung cancer in men: for all ages (1–74), the disparity is moderate and shows lower-than-average rates in a central northern zone. However, a North–south gradient is observed in men aged 40–59.
- Lung Cancer in women: higher rates are observed in the Eastern part of the country and Brussels
- Colorectal Cancer in men: medium rates in the centrum, some hot spots in a few districts of West Flanders and Hainaut, and lower than average rates in the external districts.
- Road accidents: highest rates in the South of Hainaut, Namur and Luxemburg; rather high rates along the French and German borders.

Main risk factors for **Breast Cancer** are low fecundity, late age at 1^st^ pregnancy, hormone substitution therapy, fat intake, alcohol consumption. Mortality is amenable to secondary prevention (screening strategy) followed by an adequate treatment. The breast cancer mortality has decreased substantially since 1986, while the incidence remains quite stable and one of the highest in the world [52-54]. This results from an important improvement in the survival, partly due to advances in the treatment (hormone, chemo, immunotherapy) and partly to the implementation of screening programmes. The origin of the W-E gradient in premature breast cancer mortality is currently not known and should be further explored.

The low rates of **Lung Cancer** observed in some districts attest that there is a large potential to reduce the lung cancer premature mortality: indeed, it is largely amenable to smoking eviction. Tobacco smoking represents by far the main risk factor of lung cancer, being responsible for 80-90% of the cases in the European countries. Additional risk factors are environmental pollutants: the radon and the exposure to fine particles and asbestosis. The lung cancer mortality in Belgium has first sharply increased from the 50^th^ to the end of the 90^th^ (it was multiplied by 4 in men [16]) when it was one of the highest of Europe; since then it has begun to decrease in men. At the contrary, the female lung cancer mortality rate, initially much lower than the male one, continuously increased, as the smoking behaviour of men and women evolved in opposite sense. Recent data on smoking habits show a slightly higher prevalence of daily and heavy smokers in Wallonia than in Flanders in both sexes [45]; at provincial level, the lowest rates of daily smokers were observed, for both sexes, in the two Flanders and Antwerp provinces. The highest rates were observed in the Walloon Brabant in men, in Liège, Hainaut and Limburg in women. However, because of the decennia’s long lag between exposure to tobacco use and the onset of lung cancer, the current geographical pattern in smoking habits cannot totally explain the differences in lung cancer mortality. The variation in lung cancer mortality pattern among males aged 40–59 (N-S pattern) or aged 60–74 (rather E-W pattern) could correspond in a cohort-effect in the smoking behaviour, but this should be further studied. The geographical distribution of the radon, measured by the Federal Agency of Nuclear Control, is high in some villages of the Luxemburg and the South of Namur provinces; it could possibly explain part of the geographical distribution observed for lung cancer mortality.

The main risk factor for the **Colorectal Cancer** is familial history of colorectal cancer; no strong association with a known risk factor has currently been demonstrated. The control strategy lays on secondary prevention (screening) and adequate treatment. The repartition of the premature mortality from colorectal cancer does not currently allow formulating health policy recommendations.

The **Road Accident** mortality is linked to the distance travelled, the speed, and the consumption of alcohol (and other drugs or medications). It is not surprising that very low rates are observed in big towns, where the distance to travel daily and the speed are usually small, and where the high density of public transportation allows reducing the use of cars. At the contrary, in rural areas, which are characterised by a low population density and poor public transportations, long distances are usually travelled by car on fast roads. This can partly explain the observed geographical pattern. The pattern of road accident mortality of present similarities with the one of alcohol-related deaths, and of course, alcohol consumption can be an additional explanation to the observed pattern.

### Comparison with previous findings

Grimmaud and all [42] (described the evolution of the regional differences in overall mortality since 1840; after almost a century where the life expectancy was higher in Wallonia than in Flanders, an inversion occurred after the World War II: since 1947, the life expectancy turned out to be lower in Wallonia.

Dhooghe studied all cause premature mortality by district in the periods 1948 to 1962 [5]; large between districts differences were observed, with generally lower rates in the Flemish districts for males. However, there was not yet at this time such a clear regional divide as the one observed here. Moreover, no regional difference was then reported in women.

Van Houte-Minet [6] studied the mortality only in men aged 15–75, in the years 1961–1970. An excess of mortality was observed in Wallonia for all causes mortality and in some specific causes: cardiovascular mortality (mainly ischemic heart diseases), cerebrovascular mortality, most malignant tumours, cirrhosis, COPD and external causes were higher in Wallonia. Endocrinal diseases (in particular diabetes) mortality did not show a regional difference. Stomach cancer was higher in Flanders than in Wallonia.

Humblet [13], Lagasse [14] and Leveque [15] studied the premature mortality in a selection of avoidable causes, respectively in the periods 1974–1978, 1980–1984 and 1985–1989; they all reported a disadvantage in disfavour of the Walloon districts. Humblet [7] also found a correlation between the mortality due to causes strongly related to a social gradient (for instance tuberculosis or cirrhosis) and the districts more affected by the economic recession. Those previous work already reported unfavourable results for cirrhosis in the South-West of the country and for road accidents in the provinces Limburg, Luxemburg and Namur.

In the more recent Atlas of Belgium [16] the 1991–1995 premature mortality for cardiovascular diseases, lung cancer, alcohol-related deaths, suicides and road accidents reveal the same regional patterns as the one observed during the next decennia in our study.

Many of the present findings about cause-specific mortality are in line with earlier observations. Since most unfavourable results are observed with continuity in the same part of Belgium, they should definitively be considered as warrant signal that deserve the attention of policy-makers. The regional patterns in diabetes-related mortality and in mental and neurological diseases mortality are new observations.

## Conclusions

The present study updates previous work on the geographical distribution of the mortality by cause in Belgium. A map presentation allows to highlight the geographical patterns. The choice of a relative scaling principle brings added value to conventional percentiles maps, as it stresses the magnitude of the between-district differences in premature mortality.

The between district health gap observed in this study is the result of a complex interaction between various factors, including the socioeconomic context, cultural habits and environmental influences, as well as public health policies. Some or all of those factors may have evolved differently in the different regions of Belgium, but it was not possible to disentangle the relative role of each of them in this study. Meanwhile, these results also highlight the potential for improvement in the more disadvantaged regions.

## Appendix

All tables with the cause-specific age-standardised rates by district and sex, ranked by ascending rate, along with the confidence-intervals, the smr, and the p-value of the difference with the average rate, are displayed in Additional files 26, 27, 28, 29, 30, 31, 32, 33, 34, 35, 36, 37, 38, 39, 40, 41, 42, 43, 44, 45, 46, 47, 48, 49, 50, 51, and 52.

## Additional files

Additional file 1: Figure S1. Belgium Regions Province map. Additional file 2: Figure S2. Belgium Province districts map. Additional file 3: Figure S3. MAP All cause Men4060. Additional file 4: Figure S4. MAP All cause Men6075. Additional file 5: Figure S5. MAP All cause Women4060. Additional file 6: Figure S6. MAP All cause Women6075. Additional file 7: Figure S7. MAP Cardiovascular Diseases Women175. Additional file 8: Figure S8. MAP Cerebrovasc. dis. & Hypertension Men175. Additional file 9: Figure S9. MAP Cerebrovasc. dis. & Hypertension Women175. Additional file 10: Figure S10. MAP Lung Ca Men4060. Additional file 11: Figure S11. MAP Lung Ca Men6075. Additional file 12: Figure S12. MAP Lung Ca Women4060. Additional file 13: Figure S13. MAP Lung Ca Women6075. Additional file 14: Figure S14. MAP Colorectal Ca Men175. Additional file 15: Figure S15. MAP Colorectal Ca Women175. Additional file 16: Figure S16. MAP Breast Ca Women175. Additional file 17: Figure S17. MAP Breast Ca Women4060. Additional file 18: Figure S18. MAP Breast Ca Women6075. Additional file 19: Figure S19. MAP Diabete (analysis in multiple causes) Women175. Additional file 20: Figure S20. MAP Mental & neurol. Dis (excluding alc-rel) Men175. Additional file 21: Figure S21. MAP Mental&neurol. Dis (excluding alc-rel) Women175. Additional file 22: Figure S22. MAP Alcohol-related (analysis in multiple causes) Women175. Additional file 23: Figure S22. MAP C.O.P.D. Men175. Additional file 24: Figure S24. MAP Non-Transport accident Men175. Additional file 25: Figure S25. MAP Suicide Men175. Additional file 26: Table S1. All cause Men 175. Additional file 27: Table S2. All cause Women 175. Additional file 28: Table S3. Cardiovascular Diseases Men 175. Additional file 29: Table S4. Cardiovascular Diseases Women 175. Additional file 30: Table S5. Cerebrovasc.dis. &Hypertension Men 175. Additional file 31: Table S6. Cerebrovasc. dis. & Hypertension Women 175. Additional file 32: Table S7. Lung Ca Men 175. Additional file 33: Table S8. Lung Ca Women 175. Additional file 34: Table S9. Lip, Oral Cavity, Pharynx, Larynx & oesoph. Ca Men 175. Additional file 35: Table S10. Lip, Oral Cavity, Pharynx, Larynx & oesoph. Ca Women 175. Additional file 36: Table S11. Breast Ca Women 175. Additional file 37: Table S12. Colorectal Ca Men 175. Additional file 38: Table S13. Colorectal Ca Women 175. Additional file 39: Table S14. Diabete (analysis in multiple causes) Men 175. Additional file 40: Table S15. Diabete (analysis in multiple causes) Women 175. Additional file 41: Table S16. Mental & neurol. Dis (excluding alc-rel) Men 175. Additional file 42: Table S17. Mental & neurol. Dis (excluding alc-rel) Women 175. Additional file 43: Table S18. C.O.P.D. Men 175. Additional file 44: Table S19. C.O.P.D. Women 175. Additional file 45: Table S20. Alcohol-related (analysis in multiple causes) Men 175. Additional file 46: Table S21. Alcohol-related (analysis in multiple causes) Women 175. Additional file 47: Table S22. Road Accident Men 175. Additional file 48: Table S23. Road Accident Women 175. Additional file 49: Table S24. Non-Transport accident Men 175. Additional file 50: Table S25. Non-Transport accident Women 175. Additional file 51: Table S26. Suicide Men 175. Additional file 52: Table S27. Suicide Women 175.
