# Supplementary figures and images for: Mapping the cause-specific premature mortality reveals large between-districts disparity in Belgium, 2003–2009
Source: Arch Public Health. 2015 Mar 23;73(1):13. doi: 10.1186/s13690-015-0060-5 (PMC4412101; doi:10.1186/s13690-015-0060-5)

# Regions and Provinces of Belgium

## REGIONS

- 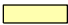 Flemish Region
- 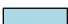 Brussels
- 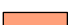 Walloon Region

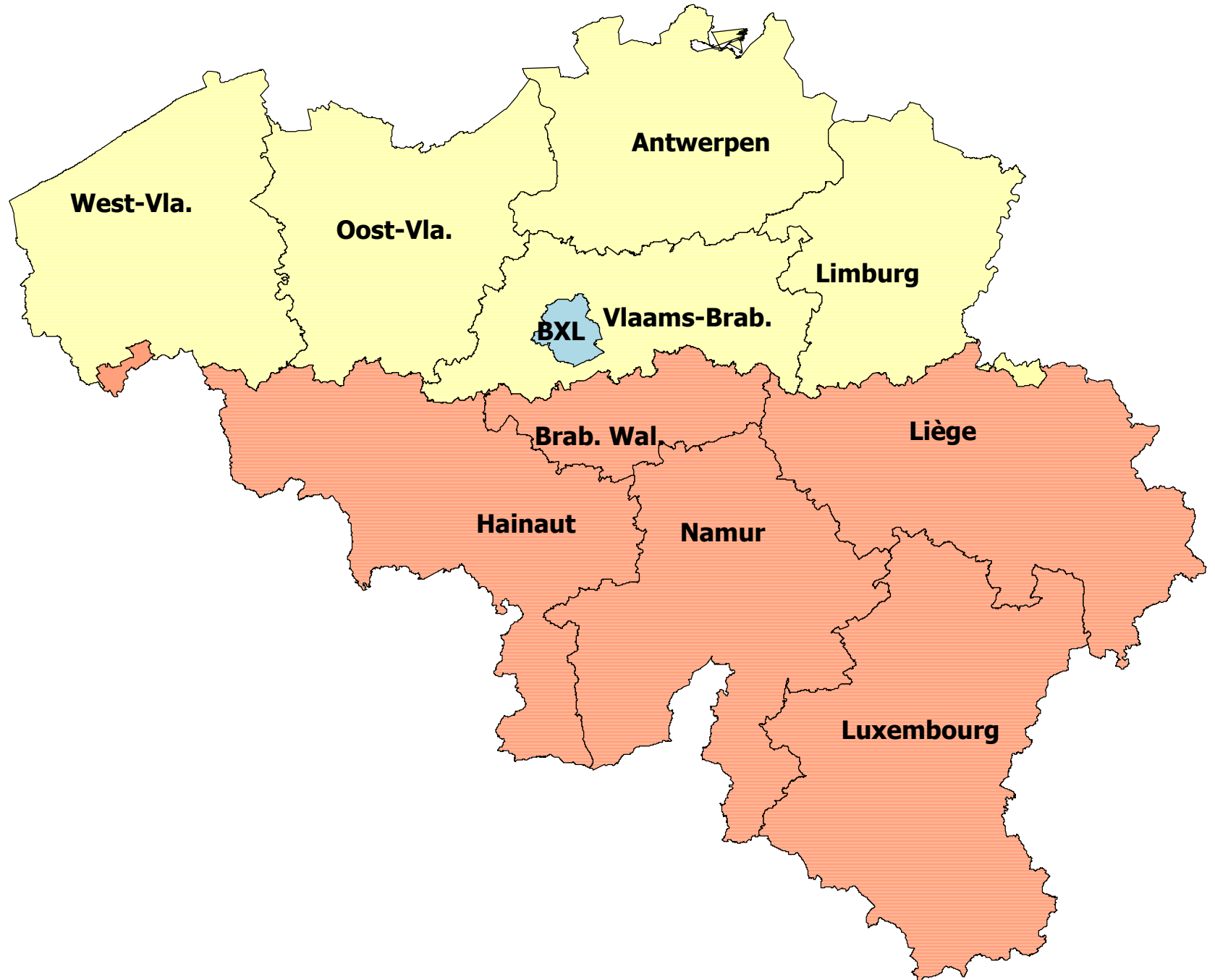

Supplement: Additional file 1: Figure S1. — Belgium Regions Province map. [file 13690_2015_60_MOESM1_ESM.pdf]
