# Supplementary material for: Mapping the cause-specific premature mortality reveals large between-districts disparity in Belgium, 2003–2009
Source: Arch Public Health. 2015 Mar 23;73(1):13. doi: 10.1186/s13690-015-0060-5 (PMC4412101; doi:10.1186/s13690-015-0060-5)
Supplement: Additional file 2: Figure S2. — Belgium Province districts map. [file 13690_2015_60_MOESM2_ESM.pdf]

Provinces and Districts of Belgium

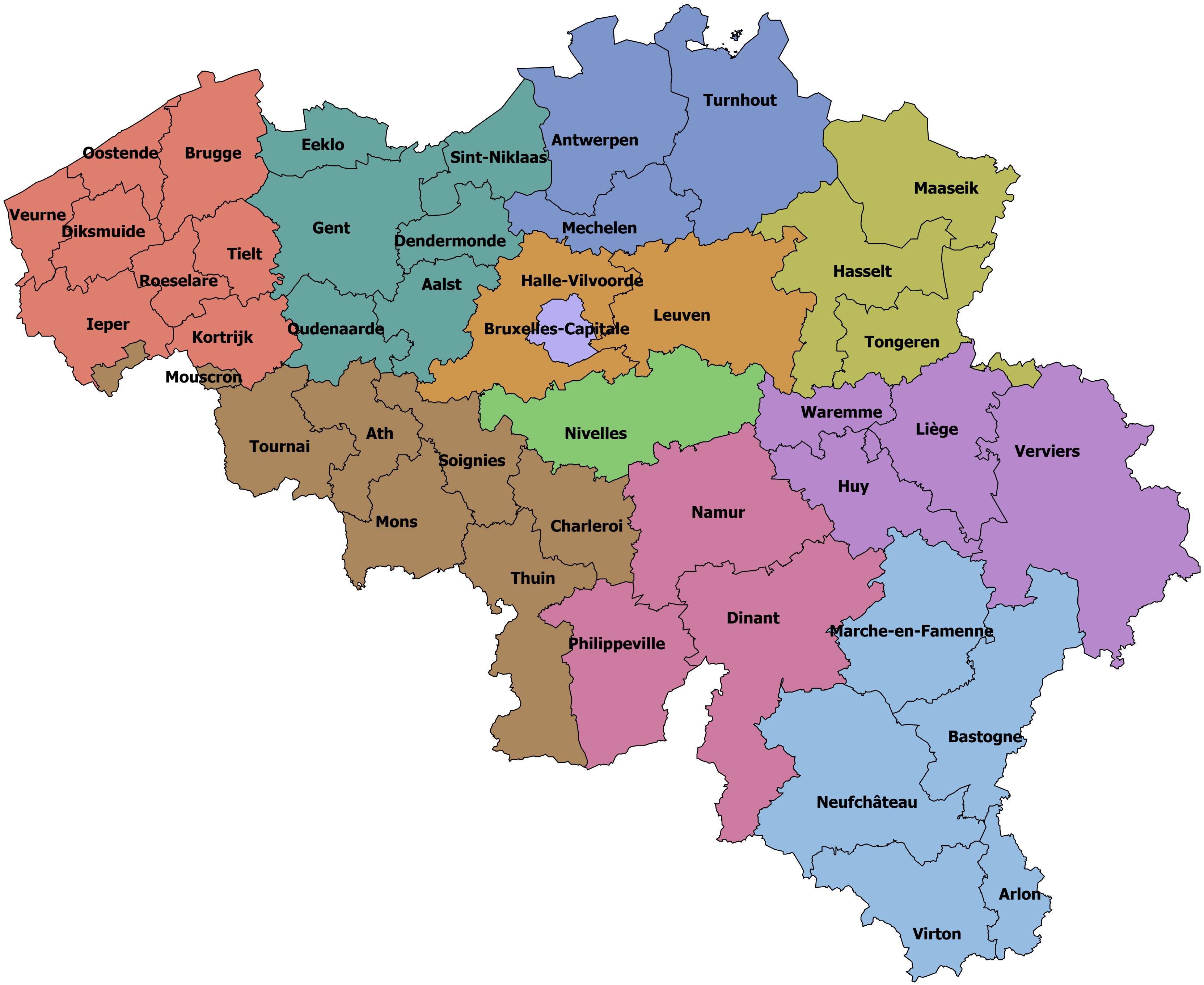

PROVINCES

- Antwerpen
- West-Vlaanderen
- Oost-Vlaanderen
- Hainaut
- Liège
- Limburg
- Luxembourg
- Namur
- Vlaams-Brabant
- Brabant Wallon
- Brussels
