# Supplementary material for: Mapping the cause-specific premature mortality reveals large between-districts disparity in Belgium, 2003–2009
Source: Arch Public Health. 2015 Mar 23;73(1):13. doi: 10.1186/s13690-015-0060-5 (PMC4412101; doi:10.1186/s13690-015-0060-5)
Supplement: Additional file 3: Figure S3. — MAP All cause Men4060. [file 13690_2015_60_MOESM3_ESM.pdf]

Mortality in Men aged 40-59 yr, Belgium 2003-2009

Age-Adjusted Mortality Rates (Std: Belgian population 2000)

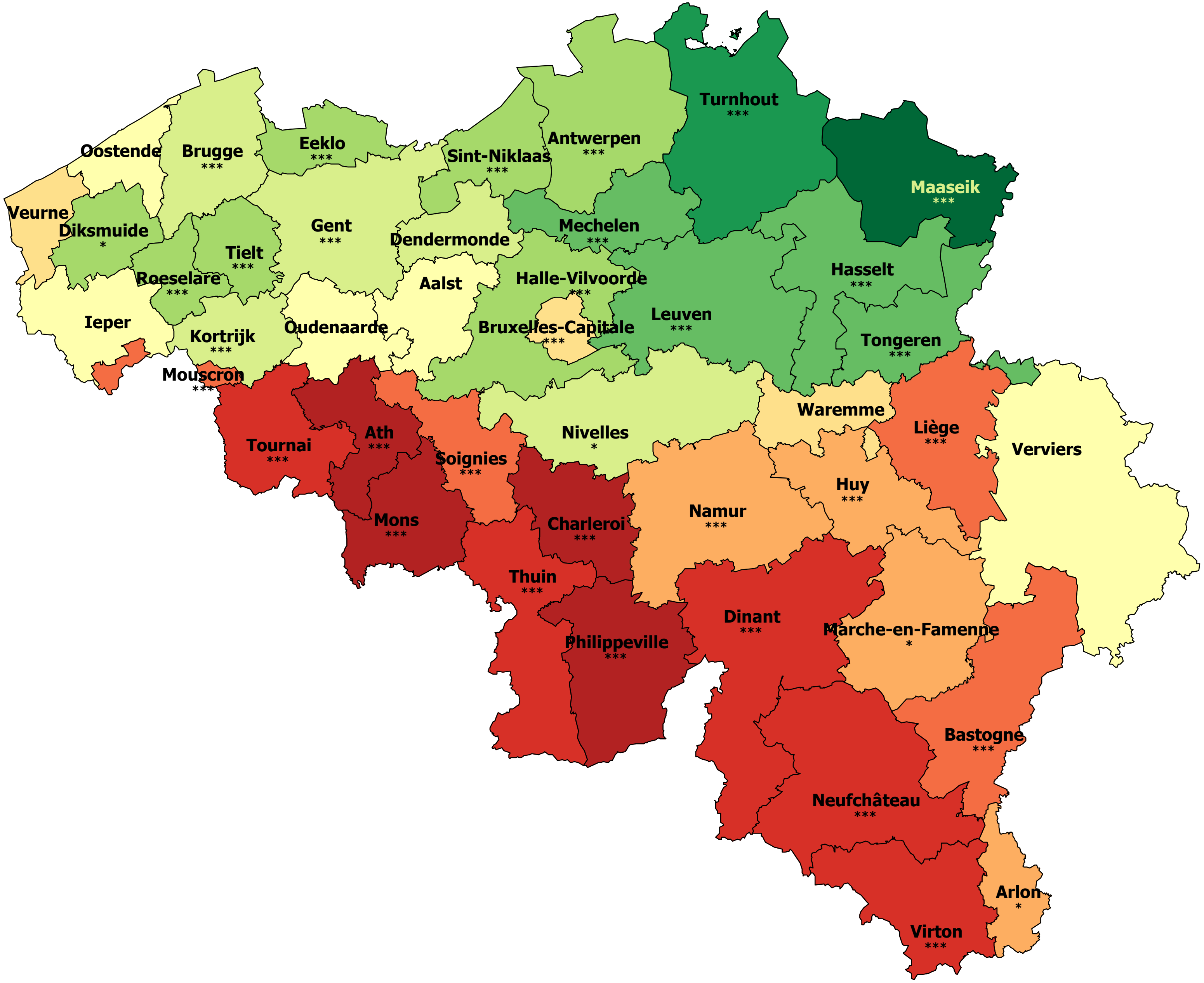

Std Rates p 100.000

- No cases
- > 0.0 - < 263.5
- 263.5 - < 289.8
- 289.8 - < 318.8
- 318.8 - < 350.7
- 350.7 - < 385.8
- 385.8 - < 424.3
- 424.3 - < 466.8 Mean= 445.0
- 466.8 - < 513.4
- 513.4 - < 564.8
- 564.8 - < 621.3
- 621.3 - < 683.4
- 683.4 - < 751.7
- >= 751.7

Range: 282 - 714 per 100.000
