# Supplementary material for: Mapping the cause-specific premature mortality reveals large between-districts disparity in Belgium, 2003–2009
Source: Arch Public Health. 2015 Mar 23;73(1):13. doi: 10.1186/s13690-015-0060-5 (PMC4412101; doi:10.1186/s13690-015-0060-5)
Supplement: Additional file 4: Figure S4. — MAP All cause Men6075. [file 13690_2015_60_MOESM4_ESM.pdf]

Mortality in Men aged 60-74 yr, Belgium 2003-2009

Age-Adjusted Mortality Rates (Std: Belgian population 2000)

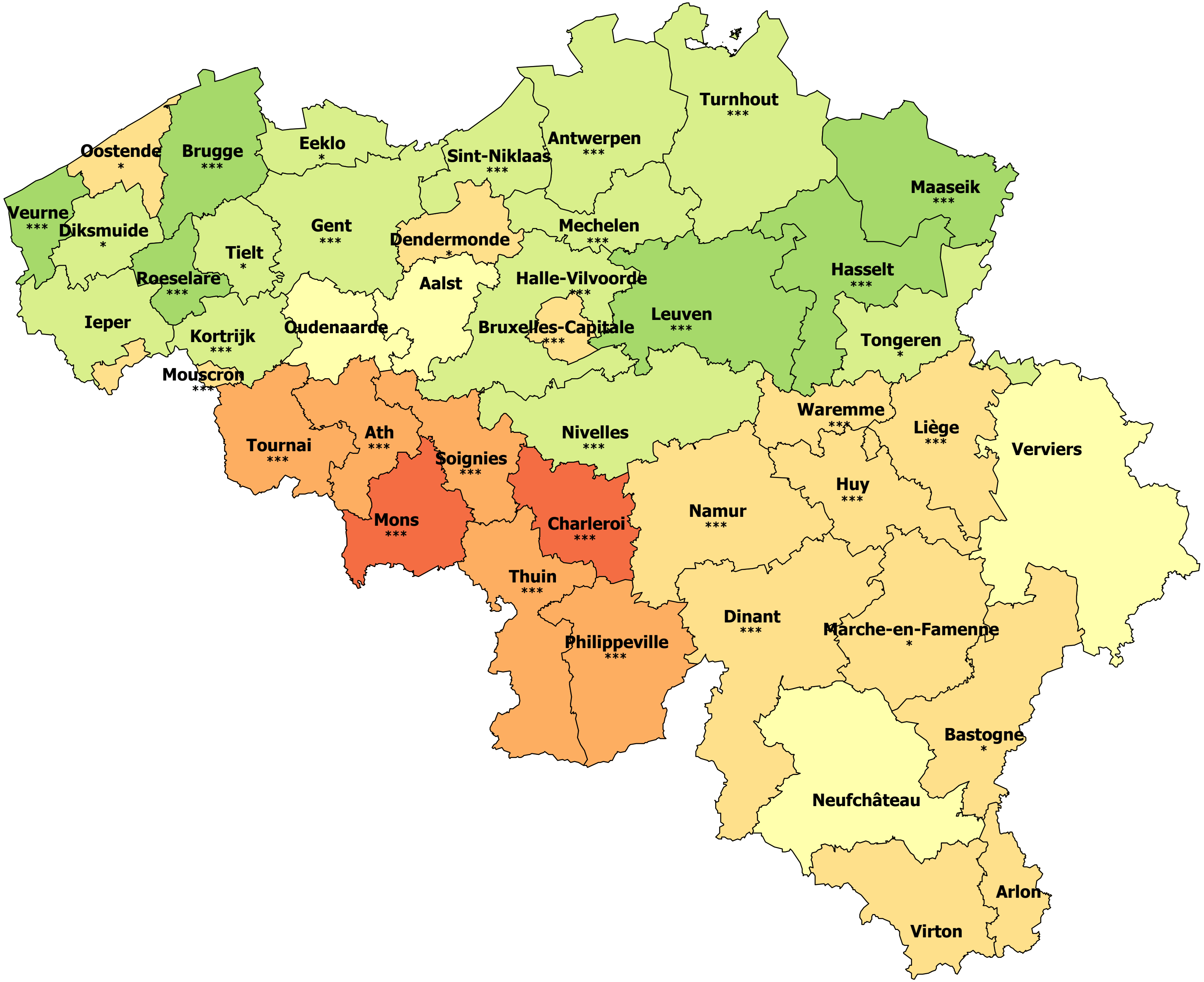

Std Rates p 100.000

- No cases
- > 0.0 - < 1201
- 1201 - < 1321
- 1321 - < 1453
- 1453 - < 1599
- 1599 - < 1759
- 1759 - < 1935
- 1935 - < 2128 Mean= 2029.0
- 2128 - < 2341
- 2341 - < 2575
- 2575 - < 2832
- 2832 - < 3116
- 3116 - < 3427
- >= 3427

Range: 1687 - 2795 per 100.000
