# Supplementary material for: Mapping the cause-specific premature mortality reveals large between-districts disparity in Belgium, 2003–2009
Source: Arch Public Health. 2015 Mar 23;73(1):13. doi: 10.1186/s13690-015-0060-5 (PMC4412101; doi:10.1186/s13690-015-0060-5)
Supplement: Additional file 5: Figure S5. — MAP All cause Women4060. [file 13690_2015_60_MOESM5_ESM.pdf]

Mortality in Women aged 40-59 yr, Belgium 2003-2009

Age-Adjusted Mortality Rates (Std: Belgian population 2000)

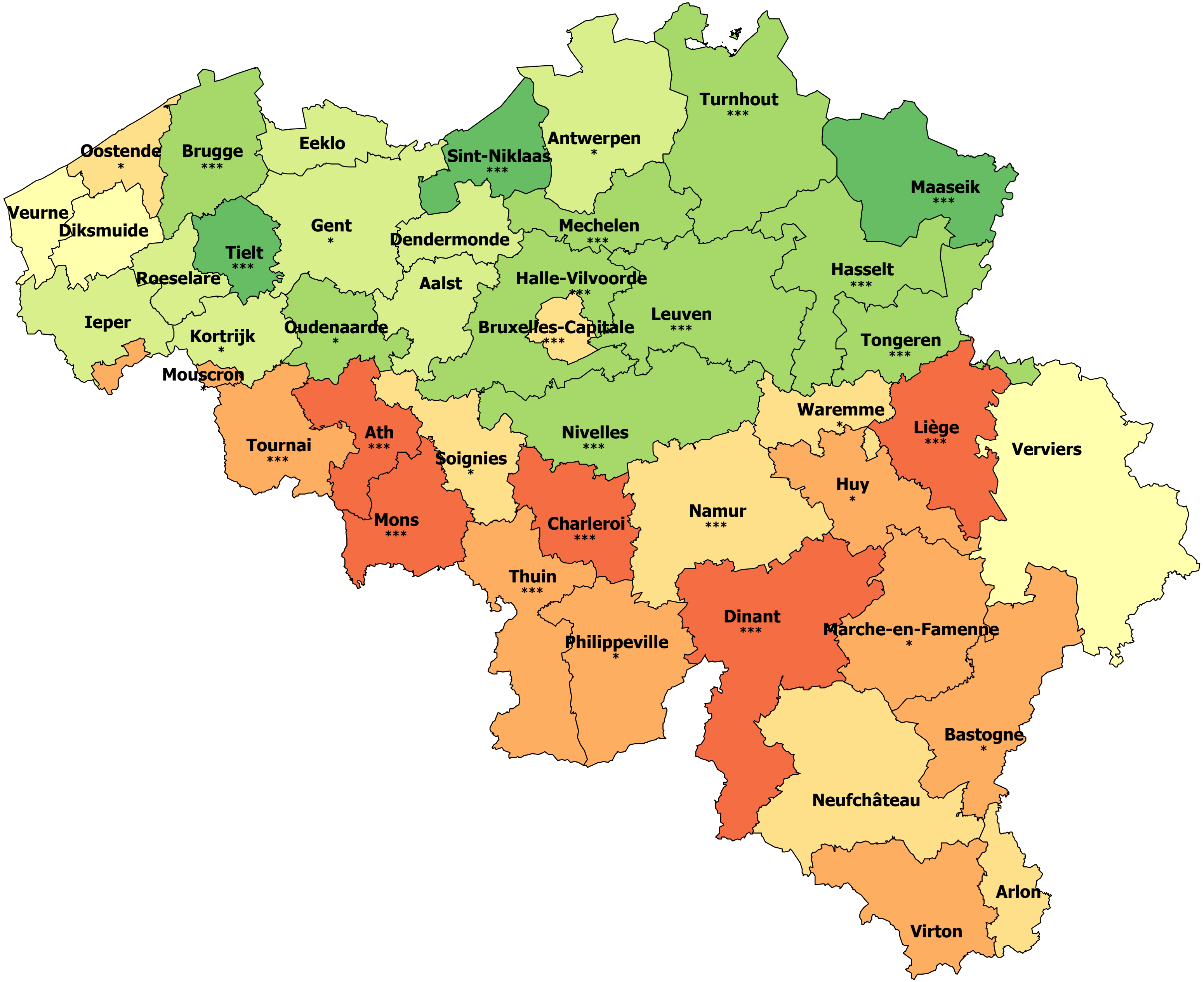

Std Rates p 100.000

- No cases
- > 0.0 - < 149.5
- 149.5 - < 164.4
- 164.4 - < 180.9
- 180.9 - < 199.0
- 199.0 - < 218.9
- 218.9 - < 240.8
- 240.8 - < 264.8 Mean= 252.5
- 264.8 - < 291.3
- 291.3 - < 320.5
- 320.5 - < 352.5
- 352.5 - < 387.7
- 387.7 - < 426.5
- >= 426.5

Range: 187 - 350 per 100.000
