# Supplementary material for: Mapping the cause-specific premature mortality reveals large between-districts disparity in Belgium, 2003–2009
Source: Arch Public Health. 2015 Mar 23;73(1):13. doi: 10.1186/s13690-015-0060-5 (PMC4412101; doi:10.1186/s13690-015-0060-5)
Supplement: Additional file 6: Figure S6. — MAP All cause Women6075. [file 13690_2015_60_MOESM6_ESM.pdf]

# Mortality in Women aged 60-74 yr, Belgium 2003-2009

## Age-Adjusted Mortality Rates (Std: Belgian population 2000)

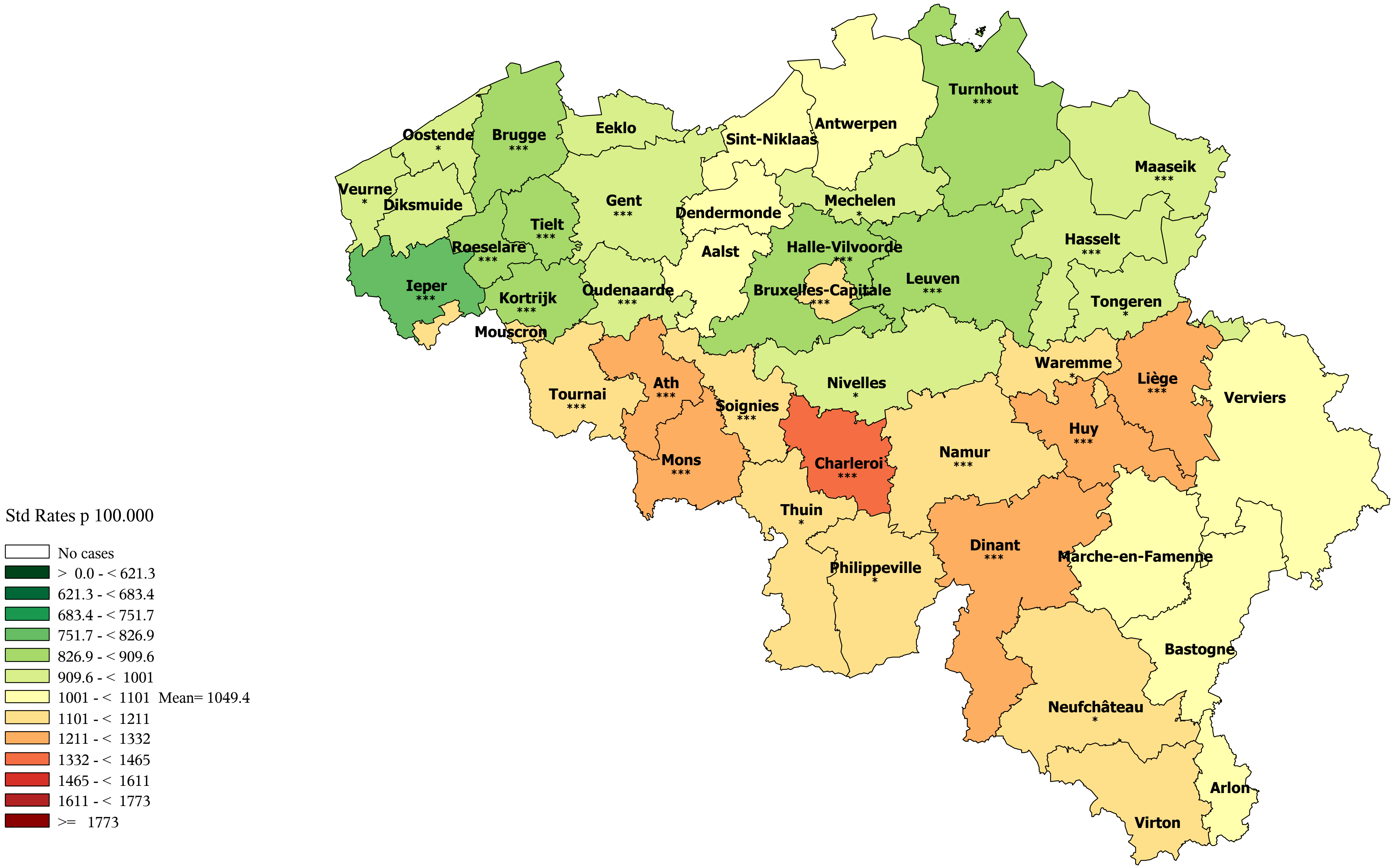


Range: 823 - 1345 per 100.000

| Directory | |
|---|---|
| **Libref** | WORK |
| **Engine** | V9 |
| **Physical Name** | C:\Users\FRenard\AppData\Local\Temp\SAS Temporary Files_TD6028_LT100219SLCDFRE_ |
| **Filename** | C:\Users\FRenard\AppData\Local\Temp\SAS Temporary Files_TD6028_LT100219SLCDFRE_ |

| # | Name | Member Type | File Size | Last Modified |
|---|---|---|---|---|
| 1 | CLASS | DATA | 17408 | 06Feb15:09:32:14 |
| 2 | FORMATS | CATALOG | 29696 | 06Feb15:09:32:14 |
| 3 | GSEG | CATALOG | 472064 | 06Feb15:09:32:15 |
| 4 | LABEL_ARR | DATA | 25600 | 06Feb15:09:32:14 |
| 5 | LABEL_ARR_0 | DATA | 13312 | 06Feb15:09:32:14 |
| 6 | LABEL_ARR_1 | DATA | 13312 | 06Feb15:09:32:14 |
| 7 | MAPARR | DATA | 414720 | 06Feb15:09:32:14 |
| 8 | MAPPROV | DATA | 9364480 | 06Feb15:09:32:14 |
| 9 | PDF | CATALOG | 472064 | 06Feb15:09:32:15 |
| 10 | RATESPYLL_ARR | DATA | 33792 | 06Feb15:09:32:14 |
| 11 | RATESPYLL_AVERAGE_2 | DATA | 9216 | 06Feb15:09:32:14 |
| 12 | RATESPYLL_GEO | DATA | 33792 | 06Feb15:09:32:14 |
| 13 | RBYSEX_GEO | DATA | 10421248 | 06Feb15:09:32:14 |
| 14 | RES1 | DATA | 9216 | 06Feb15:09:32:14 |
| 15 | SASGOPT | CATALOG | 5120 | 06Feb15:08:10:23 |
| 16 | SASMACR | CATALOG | 5120 | 06Feb15:08:10:23 |
| 17 | SELECT_2_PERC | DATA | 9216 | 06Feb15:09:32:14 |
