# Supplementary material for: Mapping the cause-specific premature mortality reveals large between-districts disparity in Belgium, 2003–2009
Source: Arch Public Health. 2015 Mar 23;73(1):13. doi: 10.1186/s13690-015-0060-5 (PMC4412101; doi:10.1186/s13690-015-0060-5)
Supplement: Additional file 10: Figure S10. — MAP Lung Ca Men4060. [file 13690_2015_60_MOESM10_ESM.pdf]

Lung Ca Mortality in Men aged 40-59 yr, Belgium 2003-2009

Age-Adjusted Mortality Rates (Std: Belgian population 2000)

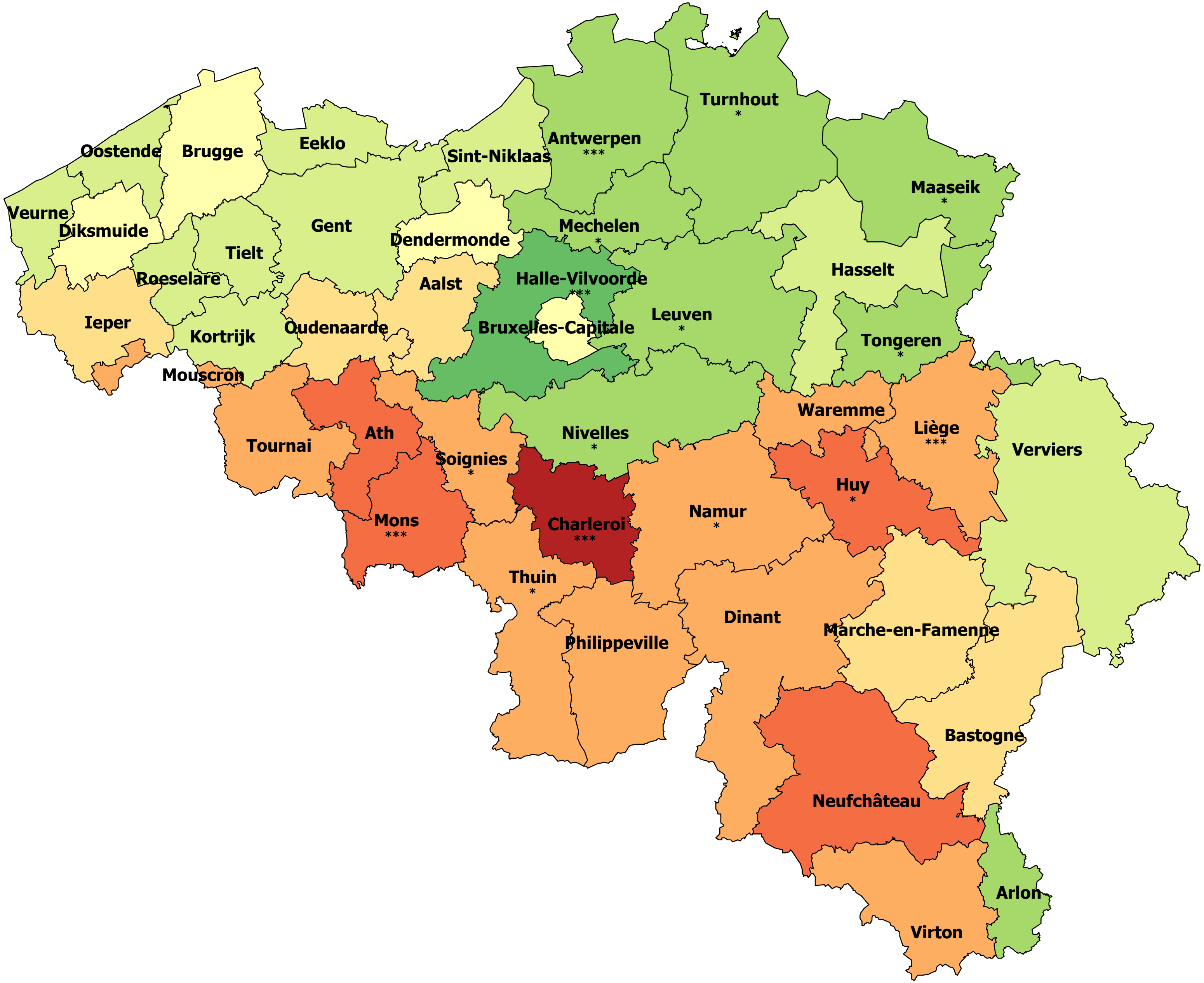

Std Rates p 100.000

- No cases
- > 0.0 - < 30.8
- 30.8 - < 33.8
- 33.8 - < 37.2
- 37.2 - < 41.0
- 41.0 - < 45.1
- 45.1 - < 49.6
- 49.6 - < 54.5 Mean= 52.0
- 54.5 - < 60.0
- 60.0 - < 66.0
- 66.0 - < 72.6
- 72.6 - < 79.8
- 79.8 - < 87.8
- >= 87.8

Range: 40 - 83 per 100.000
