# Supplementary material for: Mapping the cause-specific premature mortality reveals large between-districts disparity in Belgium, 2003–2009
Source: Arch Public Health. 2015 Mar 23;73(1):13. doi: 10.1186/s13690-015-0060-5 (PMC4412101; doi:10.1186/s13690-015-0060-5)
Supplement: Additional file 13: Figure S13. — MAP Lung Ca Women6075. [file 13690_2015_60_MOESM13_ESM.pdf]

Lung Ca Mortality in Women aged 60-74 yr, Belgium 2003-2009

Age-Adjusted Mortality Rates (Std: Belgian population 2000)

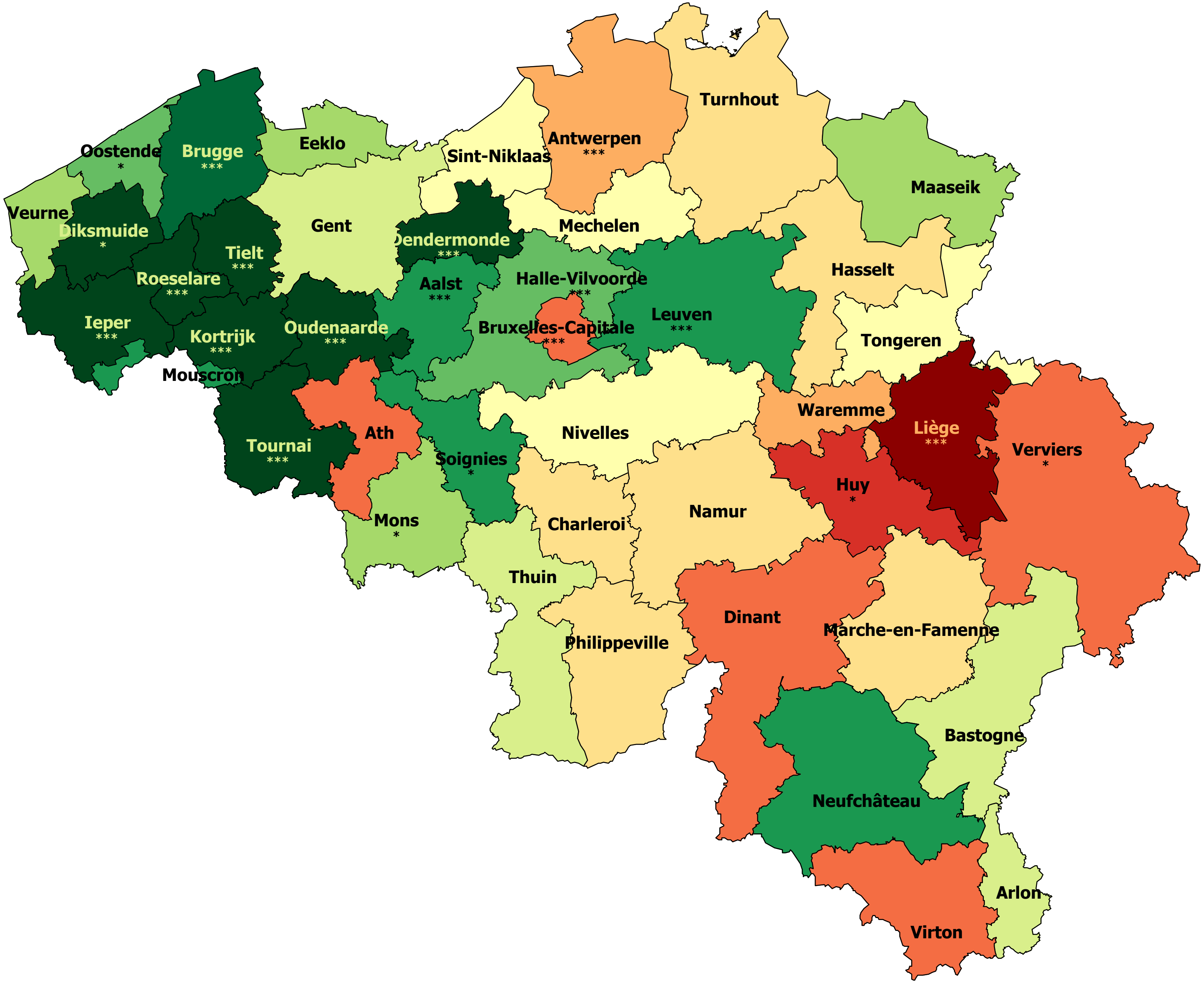

Std Rates p 100.000

- No cases
- > 0.0 - < 40.7
- 40.7 - < 44.7
- 44.7 - < 49.2
- 49.2 - < 54.1
- 54.1 - < 59.5
- 59.5 - < 65.5
- 65.5 - < 72.0 Mean= 68.7
- 72.0 - < 79.2
- 79.2 - < 87.1
- 87.1 - < 95.9
- 95.9 - < 105.4
- 105.4 - < 116.0
- >= 116.0

Range: 28 - 120 per 100.000

Directory	
**Libref**	WORK
**Engine**	V9
**Physical Name**	C:\Users\FRenard\AppData\Local\Temp\SAS Temporary Files_TD6028_LT100219SLCDFRE_
**Filename**	C:\Users\FRenard\AppData\Local\Temp\SAS Temporary Files_TD6028_LT100219SLCDFRE_

#	Name	Member Type	File Size	Last Modified
1	CLASS	DATA	17408	06Feb15:09:33:12
2	FORMATS	CATALOG	29696	06Feb15:09:33:12
3	GSEG	CATALOG	472064	06Feb15:09:33:13
4	LABEL_ARR	DATA	25600	06Feb15:09:33:12
5	LABEL_ARR_0	DATA	13312	06Feb15:09:33:12
6	LABEL_ARR_1	DATA	13312	06Feb15:09:33:12
7	MAPARR	DATA	414720	06Feb15:09:33:12
8	MAPPROV	DATA	9364480	06Feb15:09:33:12
9	PDF	CATALOG	472064	06Feb15:09:33:13
10	RATESPYLL_ARR	DATA	33792	06Feb15:09:33:12
11	RATESPYLL_AVERAGE_2	DATA	9216	06Feb15:09:33:12
12	RATESPYLL_GEO	DATA	33792	06Feb15:09:33:12
13	RBYSEX_GEO	DATA	10421248	06Feb15:09:33:12
14	RES1	DATA	9216	06Feb15:09:33:13
15	SASGOPT	CATALOG	5120	06Feb15:08:10:23
16	SASMACR	CATALOG	5120	06Feb15:08:10:23
17	SELECT_2_PERC	DATA	9216	06Feb15:09:33:12
