# Supplementary material for: Mapping the cause-specific premature mortality reveals large between-districts disparity in Belgium, 2003–2009
Source: Arch Public Health. 2015 Mar 23;73(1):13. doi: 10.1186/s13690-015-0060-5 (PMC4412101; doi:10.1186/s13690-015-0060-5)
Supplement: Additional file 15: Figure S15. — MAP Colorectal Ca Women175. [file 13690_2015_60_MOESM15_ESM.pdf]

Colorectal Ca Premature Mortality in Women (1-74 yr), Belgium 2003-2009

Age-Adjusted Mortality Rates (Std: Belgian population 2000)

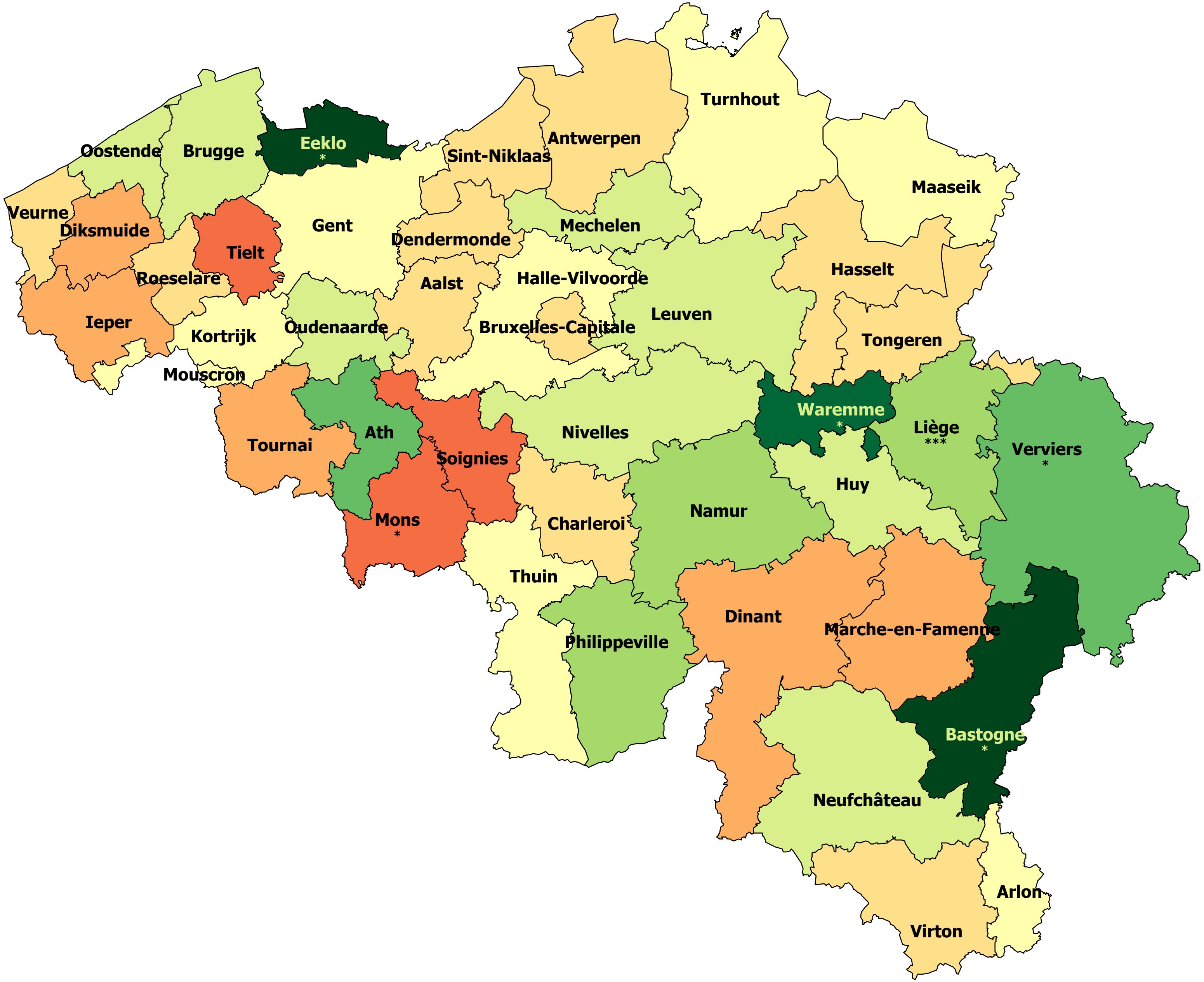

Std Rates p 100.000

- No cases
- > 0.0 - < 5.9
- 5.9 - < 6.5
- 6.5 - < 7.1
- 7.1 - < 7.8
- 7.8 - < 8.6
- 8.6 - < 9.5
- 9.5 - < 10.4 Mean= 10.0
- 10.4 - < 11.5
- 11.5 - < 12.6
- 12.6 - < 13.9
- 13.9 - < 15.3
- 15.3 - < 16.8
- >= 16.8

Range: 4 - 13 per 100.000
