# Supplementary material for: Mapping the cause-specific premature mortality reveals large between-districts disparity in Belgium, 2003–2009
Source: Arch Public Health. 2015 Mar 23;73(1):13. doi: 10.1186/s13690-015-0060-5 (PMC4412101; doi:10.1186/s13690-015-0060-5)
Supplement: Additional file 16: Figure S16. — MAP Breast Ca Women175. [file 13690_2015_60_MOESM16_ESM.pdf]

Breast Ca Premature Mortality in Women (1-74 yr), Belgium 2003-2009

Age-Adjusted Mortality Rates (Std: Belgian population 2000)

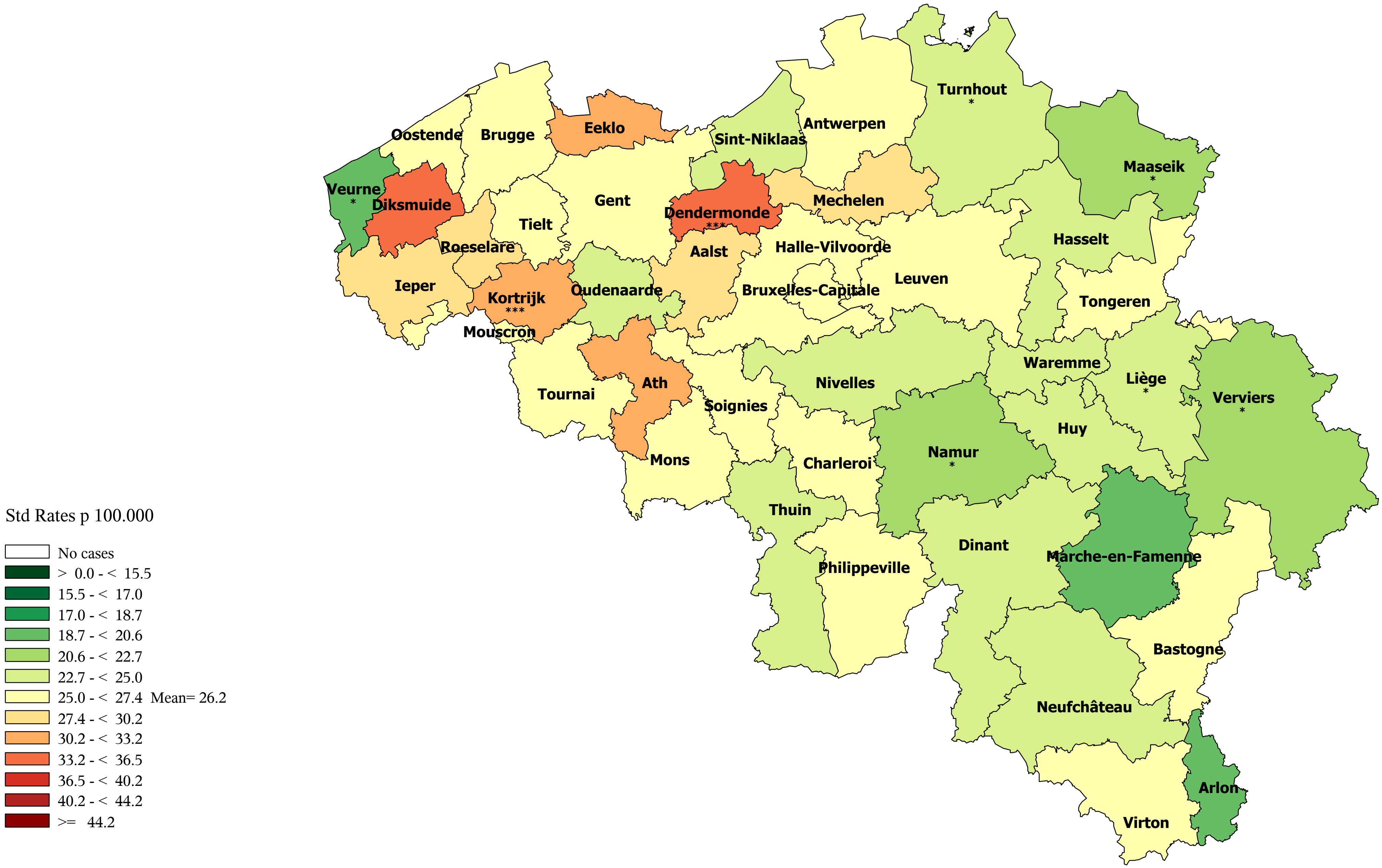

Range: 20 - 35 per 100.000
