# Supplementary material for: Mapping the cause-specific premature mortality reveals large between-districts disparity in Belgium, 2003–2009
Source: Arch Public Health. 2015 Mar 23;73(1):13. doi: 10.1186/s13690-015-0060-5 (PMC4412101; doi:10.1186/s13690-015-0060-5)
Supplement: Additional file 17: Figure S17. — MAP Breast Ca Women4060. [file 13690_2015_60_MOESM17_ESM.pdf]

Breast Ca Mortality in Women aged 40-59 yr, Belgium 2003-2009

Age-Adjusted Mortality Rates (Std: Belgian population 2000)

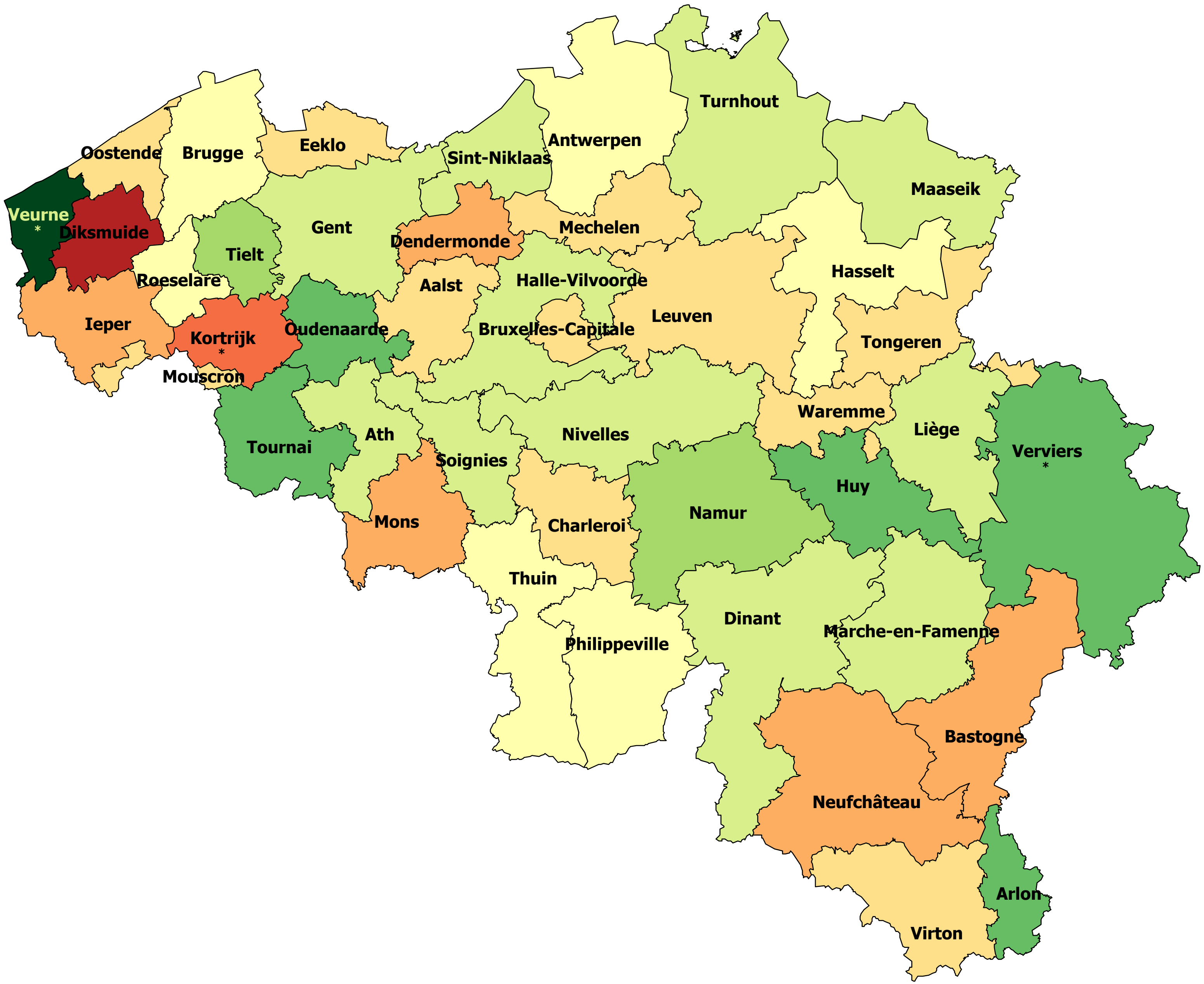

Std Rates p 100.000

- No cases
- > 0.0 - < 21.1
- 21.1 - < 23.2
- 23.2 - < 25.5
- 25.5 - < 28.1
- 28.1 - < 30.9
- 30.9 - < 34.0
- 34.0 - < 37.4 Mean= 35.6
- 37.4 - < 41.1
- 41.1 - < 45.2
- 45.2 - < 49.7
- 49.7 - < 54.7
- 54.7 - < 60.2
- >= 60.2

Range: 20 - 56 per 100.000
