# Supplementary material for: Mapping the cause-specific premature mortality reveals large between-districts disparity in Belgium, 2003–2009
Source: Arch Public Health. 2015 Mar 23;73(1):13. doi: 10.1186/s13690-015-0060-5 (PMC4412101; doi:10.1186/s13690-015-0060-5)
Supplement: Additional file 18: Figure S18. — MAP Breast Ca Women6075. [file 13690_2015_60_MOESM18_ESM.pdf]

Breast Ca Mortality in Women aged 60-74 yr, Belgium 2003-2009

Age-Adjusted Mortality Rates (Std: Belgian population 2000)

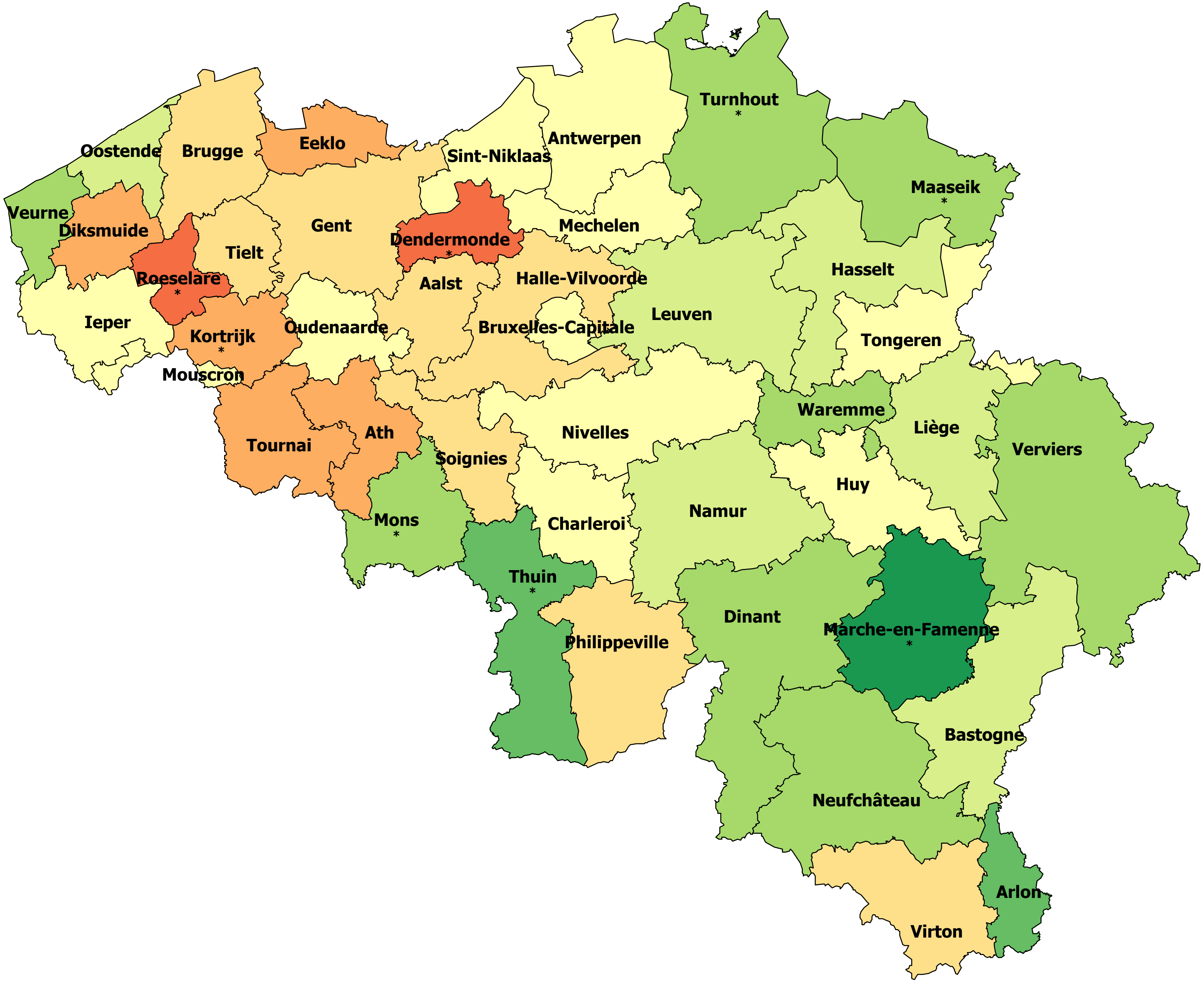

Std Rates p 100.000

- No cases
- > 0.0 - < 55.0
- 55.0 - < 60.5
- 60.5 - < 66.6
- 66.6 - < 73.2
- 73.2 - < 80.5
- 80.5 - < 88.6
- 88.6 - < 97.5 Mean= 92.9
- 97.5 - < 107.2
- 107.2 - < 117.9
- 117.9 - < 129.7
- 129.7 - < 142.7
- 142.7 - < 157.0
- >= 157.0

Range: 62 - 128 per 100.000

Directory	
**Libref**	WORK
**Engine**	V9
**Physical Name**	C:\Users\FRenard\AppData\Local\Temp\SAS Temporary Files_TD6028_LT100219SLCDFRE_
**Filename**	C:\Users\FRenard\AppData\Local\Temp\SAS Temporary Files_TD6028_LT100219SLCDFRE_

#	Name	Member Type	File Size	Last Modified
1	CLASS	DATA	17408	06Feb15:09:33:43
2	FORMATS	CATALOG	29696	06Feb15:09:33:44
3	GSEG	CATALOG	697344	06Feb15:09:33:45
4	LABEL_ARR	DATA	13312	06Feb15:09:33:44
5	LABEL_ARR_0	DATA	13312	06Feb15:09:33:44
6	LABEL_ARR_1	DATA	13312	06Feb15:09:33:44
7	MAPARR	DATA	414720	06Feb15:09:33:43
8	MAPPROV	DATA	9364480	06Feb15:09:33:43
9	PDF	CATALOG	697344	06Feb15:09:33:45
10	RATESPYLL_ARR	DATA	33792	06Feb15:09:33:43
11	RATESPYLL_AVERAGE_2	DATA	9216	06Feb15:09:33:43
12	RATESPYLL_GEO	DATA	33792	06Feb15:09:33:43
13	RBYSEX_GEO	DATA	10421248	06Feb15:09:33:43
14	RES1	DATA	9216	06Feb15:09:33:44
15	SASGOPT	CATALOG	5120	06Feb15:08:10:23
16	SASMACR	CATALOG	5120	06Feb15:08:10:23
17	SELECT_2_PERC	DATA	9216	06Feb15:09:33:44
18	TABLES	DATA	33792	06Feb15:09:33:27
