# Supplementary material for: Mapping the cause-specific premature mortality reveals large between-districts disparity in Belgium, 2003–2009
Source: Arch Public Health. 2015 Mar 23;73(1):13. doi: 10.1186/s13690-015-0060-5 (PMC4412101; doi:10.1186/s13690-015-0060-5)
Supplement: Additional file 19: Figure S19. — MAP Diabete (analysis in multiple causes) Women175. [file 13690_2015_60_MOESM19_ESM.pdf]

Diabete(analysis in multiple causes) Premature Mortality in Women (1-74 yr), Belgium 2003-2009

Age-Adjusted Mortality Rates (Std: Belgian population 2000)

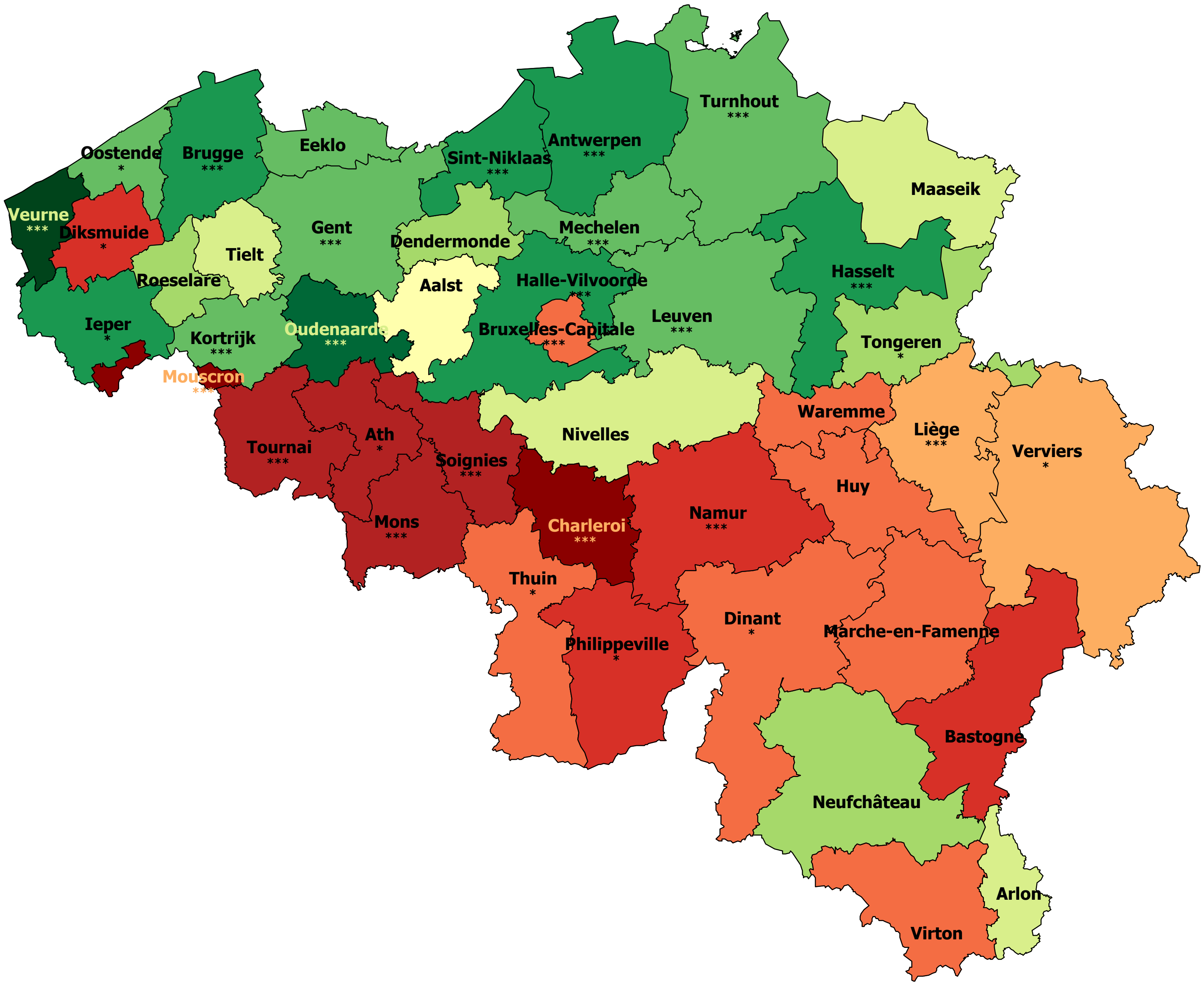

Std Rates p 100.000

- No cases
- > 0.0 - < 8.7
- 8.7 - < 9.6
- 9.6 - < 10.5
- 10.5 - < 11.6
- 11.6 - < 12.7
- 12.7 - < 14.0
- 14.0 - < 15.4 Mean= 14.7
- 15.4 - < 16.9
- 16.9 - < 18.6
- 18.6 - < 20.5
- 20.5 - < 22.6
- 22.6 - < 24.8
- >= 24.8

Range: 8 - 28 per 100.000
