# Supplementary material for: Mapping the cause-specific premature mortality reveals large between-districts disparity in Belgium, 2003–2009
Source: Arch Public Health. 2015 Mar 23;73(1):13. doi: 10.1186/s13690-015-0060-5 (PMC4412101; doi:10.1186/s13690-015-0060-5)
Supplement: Additional file 23: Figure S22. — MAP C.O.P.D. Men175. [file 13690_2015_60_MOESM23_ESM.pdf]

# Alcohol-related (analysis in multiple causes) Premature Mortality in Women (1-74 yr), Belgium 2003-2009

## Age-Adjusted Mortality Rates (Std: Belgian population 2000)

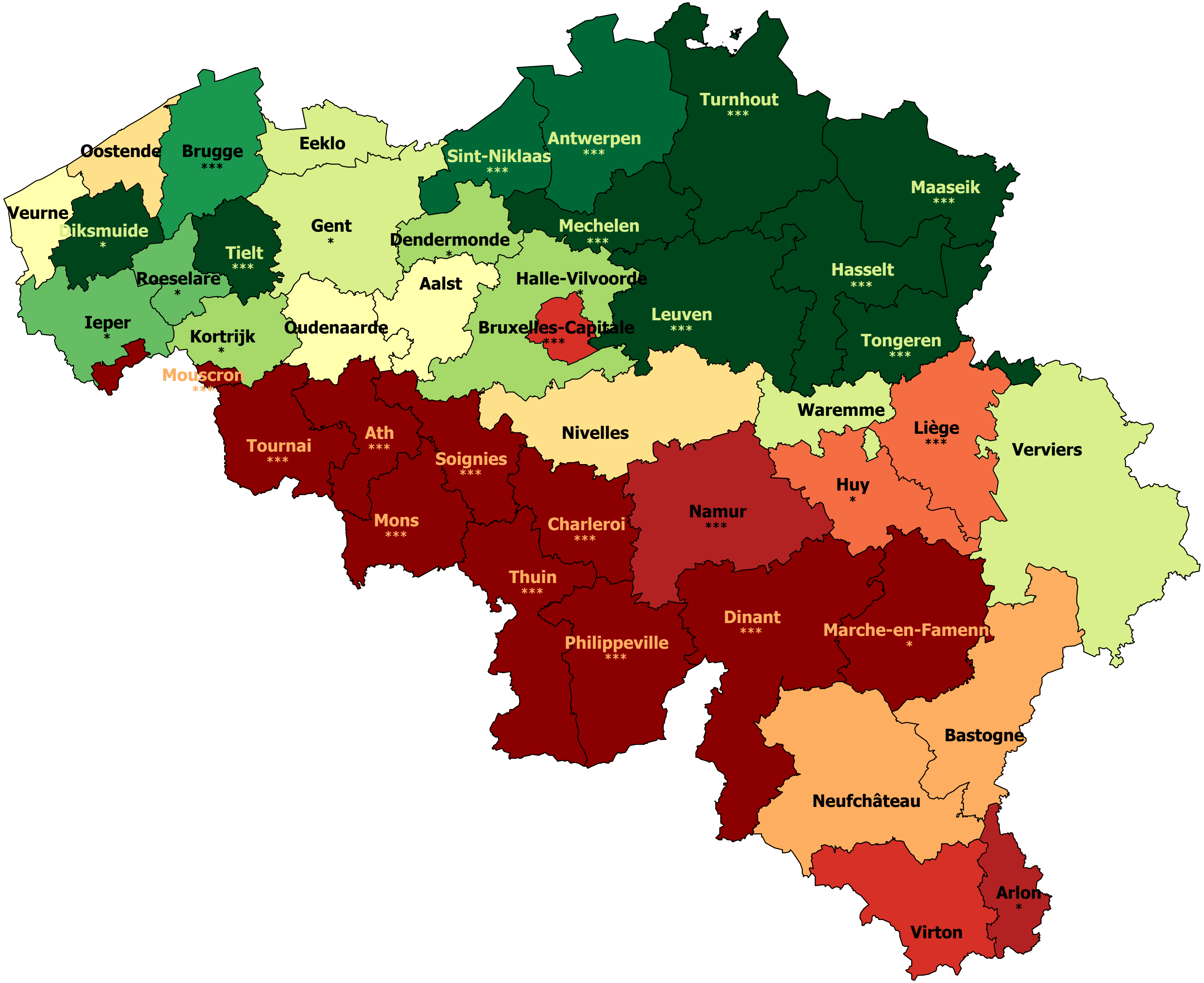


Std Rates p 100.000

- No cases
- > 0.0 - < 8.1
- 8.1 - < 8.9
- 8.9 - < 9.8
- 9.8 - < 10.8
- 10.8 - < 11.8
- 11.8 - < 13.0
- 13.0 - < 14.3 Mean= 13.7
- 14.3 - < 15.8
- 15.8 - < 17.3
- 17.3 - < 19.1
- 19.1 - < 21.0
- 21.0 - < 23.1
- >= 23.1

Range: 4 - 31 per 100.000
