# Supplementary material for: Mapping the cause-specific premature mortality reveals large between-districts disparity in Belgium, 2003–2009
Source: Arch Public Health. 2015 Mar 23;73(1):13. doi: 10.1186/s13690-015-0060-5 (PMC4412101; doi:10.1186/s13690-015-0060-5)
Supplement: Additional file 25: Figure S25. — MAP Suicide Men175. [file 13690_2015_60_MOESM25_ESM.pdf]

Suicide Premature Mortality in Men (1-74 yr), Belgium 2003-2009
Age-Adjusted Mortality Rates (Std: Belgian population 2000)

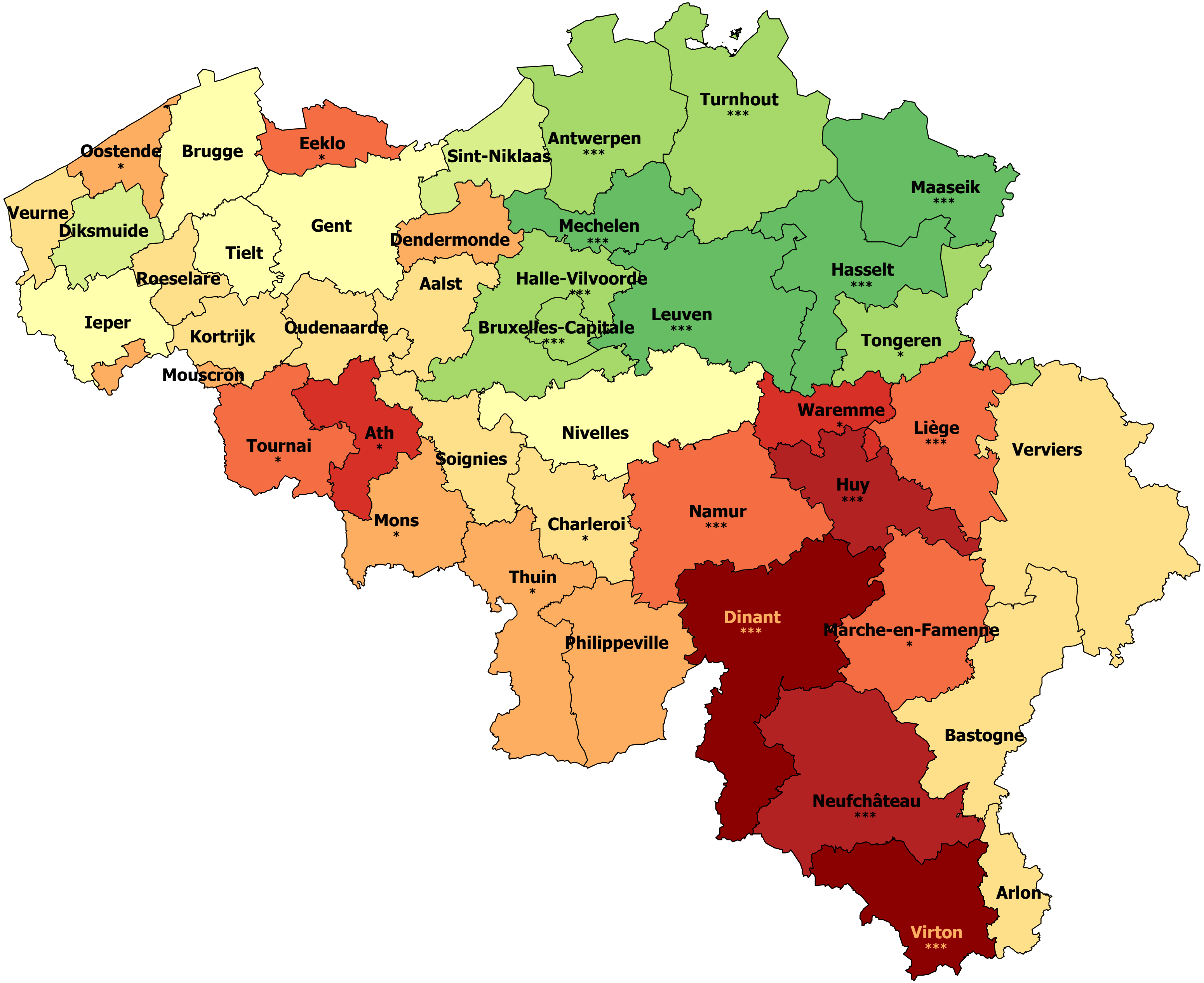

Std Rates p 100.000

- No cases
- > 0.0 - < 15.6
- 15.6 - < 17.2
- 17.2 - < 18.9
- 18.9 - < 20.8
- 20.8 - < 22.9
- 22.9 - < 25.2
- 25.2 - < 27.7 Mean= 26.4
- 27.7 - < 30.4
- 30.4 - < 33.5
- 33.5 - < 36.8
- 36.8 - < 40.5
- 40.5 - < 44.6
- >= 44.6

Range: 19 - 46 per 100.000
