# Supplementary material for: Mapping the cause-specific premature mortality reveals large between-districts disparity in Belgium, 2003–2009
Source: Arch Public Health. 2015 Mar 23;73(1):13. doi: 10.1186/s13690-015-0060-5 (PMC4412101; doi:10.1186/s13690-015-0060-5)
Supplement: Additional file 26: Table S1. — All cause Men 175. [file 13690_2015_60_MOESM26_ESM.zip › 13690_2015_60_MOESM26_ESM.html]

SAS Output


# Premature Mortality in Men (1-74 yr), Belgium 2003-2009

# Ranking of the arrondissements by increased mortality

# Age-adjusted rates per 100.000

| Rank | ARROND | Age-adj.Rates | CI on age-adj.Rates | smr | p value\* |
| --- | --- | --- | --- | --- | --- |
| 1 | Maaseik | 384.5 | [ 370; 399] | 77.3 | <0.001 |
| 2 | Turnhout | 398.2 | [ 388; 409] | 80.2 | <0.001 |
| 3 | Leuven | 398.8 | [ 389; 409] | 80.9 | <0.001 |
| 4 | Hasselt | 404.4 | [ 393; 416] | 81.7 | <0.001 |
| 5 | Halle-Vilvoorde | 422.6 | [ 413; 432] | 85.9 | <0.001 |
| 6 | Roeselare | 423.4 | [ 405; 442] | 86.2 | <0.001 |
| 7 | Mechelen | 426.0 | [ 413; 439] | 86.7 | <0.001 |
| 8 | Tongeren | 426.4 | [ 410; 443] | 85.8 | <0.001 |
| 9 | Diksmuide | 427.1 | [ 395; 460] | 87.0 | <0.001 |
| 10 | Brugge | 427.3 | [ 414; 440] | 86.9 | <0.001 |
| 11 | Sint Niklaas | 432.1 | [ 417; 447] | 87.8 | <0.001 |
| 12 | Antwerpen | 433.4 | [ 426; 441] | 88.0 | <0.001 |
| 13 | Tielt | 433.6 | [ 409; 458] | 88.1 | <0.001 |
| 14 | Kortrijk | 447.8 | [ 434; 462] | 91.4 | <0.001 |
| 15 | Gent | 448.1 | [ 438; 459] | 91.4 | <0.001 |
| 16 | Eeklo | 448.7 | [ 424; 474] | 91.0 | <0.01 |
| 17 | Veurne | 456.8 | [ 429; 485] | 92.3 | <0.05 |
| 18 | Nivelles | 460.4 | [ 447; 473] | 93.7 | <0.001 |
| 19 | Ieper | 471.8 | [ 448; 495] | 96.5 | ns. |
| 20 | Oudenaarde | 484.5 | [ 462; 507] | 98.8 | ns. |
| 21 | Aalst | 498.8 | [ 484; 514] | 101.9 | ns. |
| 22 | Dendermonde | 499.9 | [ 482; 518] | 101.5 | ns. |
| 23 | Verviers | 508.4 | [ 493; 524] | 103.5 | <0.05 |
| 24 | Oostende | 520.1 | [ 501; 539] | 105.7 | <0.01 |
| 25 | Brussels | 524.0 | [ 515; 533] | 106.0 | <0.001 |
| 26 | Arlon | 543.4 | [ 506; 581] | 111.7 | <0.01 |
| 27 | Waremme | 552.7 | [ 520; 585] | 112.0 | <0.001 |
| 28 | Marche-en-Famenne | 572.0 | [ 534; 610] | 118.0 | <0.001 |
| 29 | Neufchateau | 573.4 | [ 537; 610] | 119.2 | <0.001 |
| 30 | Bastogne | 576.0 | [ 532; 620] | 118.6 | <0.001 |
| 31 | Huy | 577.1 | [ 550; 605] | 118.1 | <0.001 |
| 32 | Namur | 580.2 | [ 564; 597] | 119.2 | <0.001 |
| 33 | Li�ge | 584.5 | [ 573; 596] | 119.6 | <0.001 |
| 34 | Mouscron | 587.3 | [ 554; 620] | 120.3 | <0.001 |
| 35 | Virton | 592.6 | [ 552; 633] | 121.8 | <0.001 |
| 36 | Dinant | 613.3 | [ 586; 641] | 126.0 | <0.001 |
| 37 | Soignies | 620.9 | [ 599; 643] | 127.0 | <0.001 |
| 38 | Tournai | 639.6 | [ 615; 664] | 131.5 | <0.001 |
| 39 | Philippeville | 647.8 | [ 612; 684] | 134.3 | <0.001 |
| 40 | Thuin | 648.9 | [ 625; 673] | 133.3 | <0.001 |
| 41 | Ath | 659.7 | [ 627; 693] | 135.6 | <0.001 |
| 42 | Charleroi | 697.0 | [ 682; 712] | 143.1 | <0.001 |
| 43 | Mons | 700.4 | [ 680; 720] | 144.1 | <0.001 |

  

# Mean Rate = 490.5

# 

# \* p value of the z statistic testing for a the difference between the arrondissement's rate and the mean rate
