# Supplementary material for: Mapping the cause-specific premature mortality reveals large between-districts disparity in Belgium, 2003–2009
Source: Arch Public Health. 2015 Mar 23;73(1):13. doi: 10.1186/s13690-015-0060-5 (PMC4412101; doi:10.1186/s13690-015-0060-5)
Supplement: Additional file 27: Table S2. — All cause Women 175. [file 13690_2015_60_MOESM27_ESM.zip › 13690_2015_60_MOESM27_ESM.html]

SAS Output


# Premature Mortality in Women (1-74 yr), Belgium 2003-2009

# Ranking of the arrondissements by increased mortality

# Age-adjusted rates per 100.000

| Rank | ARROND | Age-adj.Rates | CI on age-adj.Rates | smr | p value\* |
| --- | --- | --- | --- | --- | --- |
| 1 | Tielt | 206.8 | [ 190; 223] | 79.8 | <0.001 |
| 2 | Leuven | 210.6 | [ 203; 218] | 81.7 | <0.001 |
| 3 | Ieper | 211.0 | [ 196; 226] | 82.0 | <0.001 |
| 4 | Maaseik | 215.9 | [ 205; 227] | 83.5 | <0.001 |
| 5 | Turnhout | 217.2 | [ 209; 225] | 84.1 | <0.001 |
| 6 | Brugge | 220.9 | [ 212; 230] | 85.4 | <0.001 |
| 7 | Halle-Vilvoorde | 220.9 | [ 214; 227] | 85.7 | <0.001 |
| 8 | Kortrijk | 222.8 | [ 213; 232] | 86.3 | <0.001 |
| 9 | Oudenaarde | 223.6 | [ 209; 238] | 87.4 | <0.001 |
| 10 | Roeselare | 226.5 | [ 213; 240] | 87.4 | <0.001 |
| 11 | Hasselt | 227.6 | [ 219; 236] | 88.1 | <0.001 |
| 12 | Tongeren | 230.5 | [ 219; 242] | 88.8 | <0.001 |
| 13 | Mechelen | 232.3 | [ 223; 241] | 90.4 | <0.001 |
| 14 | Sint Niklaas | 234.3 | [ 223; 245] | 91.0 | <0.001 |
| 15 | Veurne | 234.9 | [ 215; 255] | 90.0 | <0.05 |
| 16 | Nivelles | 235.1 | [ 226; 244] | 90.9 | <0.001 |
| 17 | Gent | 238.2 | [ 231; 246] | 92.1 | <0.001 |
| 18 | Eeklo | 241.5 | [ 223; 260] | 92.5 | ns. |
| 19 | Diksmuide | 247.3 | [ 223; 272] | 96.1 | ns. |
| 20 | Oostende | 248.0 | [ 235; 261] | 95.7 | ns. |
| 21 | Aalst | 249.9 | [ 240; 260] | 96.8 | ns. |
| 22 | Antwerpen | 254.1 | [ 249; 260] | 98.6 | ns. |
| 23 | Verviers | 260.5 | [ 250; 271] | 101.1 | ns. |
| 24 | Dendermonde | 260.8 | [ 248; 273] | 101.0 | ns. |
| 25 | Arlon | 260.9 | [ 236; 285] | 101.6 | ns. |
| 26 | Bastogne | 271.9 | [ 243; 301] | 105.6 | ns. |
| 27 | Marche-en-Famenne | 277.8 | [ 252; 303] | 107.7 | ns. |
| 28 | Mouscron | 280.3 | [ 259; 302] | 108.6 | <0.05 |
| 29 | Virton | 285.8 | [ 259; 313] | 110.5 | <0.05 |
| 30 | Neufchateau | 287.9 | [ 263; 313] | 111.2 | <0.05 |
| 31 | Soignies | 289.8 | [ 276; 304] | 112.1 | <0.001 |
| 32 | Brussels | 291.0 | [ 285; 297] | 112.6 | <0.001 |
| 33 | Namur | 292.0 | [ 281; 303] | 113.3 | <0.001 |
| 34 | Waremme | 292.9 | [ 271; 315] | 113.5 | <0.01 |
| 35 | Thuin | 294.4 | [ 279; 310] | 114.3 | <0.001 |
| 36 | Philippeville | 295.2 | [ 272; 318] | 115.0 | <0.01 |
| 37 | Tournai | 305.0 | [ 289; 321] | 118.0 | <0.001 |
| 38 | Dinant | 313.4 | [ 295; 332] | 121.2 | <0.001 |
| 39 | Ath | 317.1 | [ 296; 338] | 122.7 | <0.001 |
| 40 | Huy | 317.6 | [ 298; 337] | 122.9 | <0.001 |
| 41 | Li�ge | 321.4 | [ 314; 329] | 124.1 | <0.001 |
| 42 | Mons | 323.2 | [ 311; 336] | 125.1 | <0.001 |
| 43 | Charleroi | 339.4 | [ 330; 349] | 131.7 | <0.001 |

  

# Mean Rate = 257.9

# 

# \* p value of the z statistic testing for a the difference between the arrondissement's rate and the mean rate
