# Supplementary material for: Mapping the cause-specific premature mortality reveals large between-districts disparity in Belgium, 2003–2009
Source: Arch Public Health. 2015 Mar 23;73(1):13. doi: 10.1186/s13690-015-0060-5 (PMC4412101; doi:10.1186/s13690-015-0060-5)
Supplement: Additional file 28: Table S3. — Cardiovascular Diseases Men 175. [file 13690_2015_60_MOESM28_ESM.zip › 13690_2015_60_MOESM28_ESM.html]

SAS Output


# Cardiovascular Diseases Premature Mortality in Men (1-74 yr), Belgium 2003-2009

# Ranking of the arrondissements by increased mortality

# Age-adjusted rates per 100.000

| Rank | ARROND | Age-adj.Rates | CI on age-adj.Rates | smr | p value\* |
| --- | --- | --- | --- | --- | --- |
| 1 | Maaseik | 80.8 | [74.3;87.4] | 81.2 | <0.001 |
| 2 | Nivelles | 81.4 | [75.8;86.9] | 82.5 | <0.001 |
| 3 | Sint Niklaas | 83.4 | [76.7;90.0] | 84.8 | <0.001 |
| 4 | Hasselt | 84.8 | [79.7;90.0] | 85.6 | <0.001 |
| 5 | Halle-Vilvoorde | 85.2 | [80.9;89.5] | 86.5 | <0.001 |
| 6 | Turnhout | 86.7 | [81.7;91.6] | 87.1 | <0.001 |
| 7 | Leuven | 87.1 | [82.3;91.9] | 88.7 | <0.001 |
| 8 | Eeklo | 88.1 | [77.0;99.2] | 89.0 | ns. |
| 9 | Antwerpen | 88.7 | [85.4;92.1] | 90.2 | <0.001 |
| 10 | Brugge | 89.1 | [83.1;95.1] | 90.4 | <0.01 |
| 11 | Gent | 89.5 | [84.8;94.2] | 91.4 | <0.001 |
| 12 | Tongeren | 89.7 | [82.3;97.2] | 90.6 | <0.05 |
| 13 | Mouscron | 91.1 | [77.9; 104] | 90.6 | ns. |
| 14 | Kortrijk | 92.1 | [85.9;98.4] | 93.8 | ns. |
| 15 | Diksmuide | 92.8 | [77.7; 108] | 94.9 | ns. |
| 16 | Mechelen | 92.8 | [86.9;98.8] | 94.7 | ns. |
| 17 | Tielt | 93.4 | [82.2; 105] | 94.6 | ns. |
| 18 | Roeselare | 93.8 | [85.0; 103] | 94.9 | ns. |
| 19 | Oudenaarde | 97.3 | [87.3; 107] | 99.0 | ns. |
| 20 | Bastogne | 97.9 | [79.8; 116] | 102.2 | ns. |
| 21 | Dendermonde | 98.0 | [90.1; 106] | 98.5 | ns. |
| 22 | Ieper | 98.5 | [87.8; 109] | 100.4 | ns. |
| 23 | Arlon | 99.2 | [83.1; 115] | 101.5 | ns. |
| 24 | Veurne | 100.2 | [87.4; 113] | 101.4 | ns. |
| 25 | Aalst | 102.1 | [95.4; 109] | 103.9 | ns. |
| 26 | Neufchateau | 103.4 | [87.9; 119] | 106.3 | ns. |
| 27 | Dinant | 105.5 | [94.0; 117] | 107.4 | ns. |
| 28 | Namur | 106.3 | [99.1; 113] | 108.4 | <0.05 |
| 29 | Tournai | 106.6 | [96.4; 117] | 106.7 | ns. |
| 30 | Virton | 107.4 | [90.1; 125] | 110.5 | ns. |
| 31 | Oostende | 107.5 | [98.8; 116] | 108.5 | <0.05 |
| 32 | Huy | 107.6 | [95.7; 119] | 111.6 | ns. |
| 33 | Brussels | 108.9 | [ 105; 113] | 110.3 | <0.001 |
| 34 | Verviers | 110.4 | [ 103; 118] | 111.7 | <0.01 |
| 35 | Li�ge | 112.3 | [ 107; 117] | 114.3 | <0.001 |
| 36 | Marche-en-Famenne | 115.6 | [98.4; 133] | 118.0 | <0.05 |
| 37 | Soignies | 120.2 | [ 110; 130] | 121.6 | <0.001 |
| 38 | Waremme | 122.3 | [ 107; 138] | 124.4 | <0.01 |
| 39 | Thuin | 122.7 | [ 112; 133] | 125.8 | <0.001 |
| 40 | Mons | 125.3 | [ 117; 134] | 129.2 | <0.001 |
| 41 | Ath | 126.3 | [ 112; 141] | 127.3 | <0.001 |
| 42 | Philippeville | 134.5 | [ 118; 151] | 139.7 | <0.001 |
| 43 | Charleroi | 138.2 | [ 131; 145] | 142.1 | <0.001 |

  

# Mean Rate = 98.4

# 

# \* p value of the z statistic testing for a the difference between the arrondissement's rate and the mean rate
