# Supplementary material for: Mapping the cause-specific premature mortality reveals large between-districts disparity in Belgium, 2003–2009
Source: Arch Public Health. 2015 Mar 23;73(1):13. doi: 10.1186/s13690-015-0060-5 (PMC4412101; doi:10.1186/s13690-015-0060-5)
Supplement: Additional file 29: Table S4. — Cardiovascular Diseases Women 175. [file 13690_2015_60_MOESM29_ESM.zip › 13690_2015_60_MOESM29_ESM.html]

SAS Output


# Cardiovascular Diseases Premature Mortality in Women (1-74 yr), Belgium 2003-2009

# Ranking of the arrondissements by increased mortality

# Age-adjusted rates per 100.000

| Rank | ARROND | Age-adj.Rates | CI on age-adj.Rates | smr | p value\* |
| --- | --- | --- | --- | --- | --- |
| 1 | Nivelles | 27.7 | [24.6;30.7] | 71.2 | <0.001 |
| 2 | Tielt | 28.1 | [22.1;34.1] | 72.7 | <0.001 |
| 3 | Eeklo | 28.7 | [22.5;34.9] | 72.9 | <0.01 |
| 4 | Leuven | 30.3 | [27.6;33.0] | 77.8 | <0.001 |
| 5 | Ieper | 31.0 | [25.2;36.8] | 79.3 | <0.01 |
| 6 | Kortrijk | 31.5 | [27.9;35.0] | 80.4 | <0.001 |
| 7 | Brugge | 31.5 | [28.1;34.9] | 80.7 | <0.001 |
| 8 | Halle-Vilvoorde | 31.8 | [29.3;34.2] | 81.5 | <0.001 |
| 9 | Mouscron | 33.0 | [25.8;40.3] | 85.6 | ns. |
| 10 | Gent | 33.4 | [30.7;36.1] | 85.4 | <0.001 |
| 11 | Oudenaarde | 33.5 | [27.9;39.1] | 86.3 | ns. |
| 12 | Turnhout | 34.0 | [30.9;37.1] | 87.1 | <0.01 |
| 13 | Arlon | 34.5 | [25.4;43.5] | 87.3 | ns. |
| 14 | Veurne | 36.1 | [28.7;43.6] | 91.3 | ns. |
| 15 | Maaseik | 36.2 | [31.8;40.5] | 92.4 | ns. |
| 16 | Mechelen | 36.2 | [32.6;39.8] | 92.6 | ns. |
| 17 | Hasselt | 36.4 | [33.1;39.6] | 93.2 | ns. |
| 18 | Virton | 37.5 | [27.8;47.2] | 96.8 | ns. |
| 19 | Sint Niklaas | 37.8 | [33.4;42.1] | 96.9 | ns. |
| 20 | Antwerpen | 38.0 | [35.9;40.1] | 97.3 | ns. |
| 21 | Roeselare | 38.5 | [33.1;44.0] | 98.8 | ns. |
| 22 | Ath | 38.8 | [31.3;46.3] | 100.0 | ns. |
| 23 | Tongeren | 39.2 | [34.4;44.0] | 99.8 | ns. |
| 24 | Philippeville | 39.2 | [30.8;47.6] | 101.6 | ns. |
| 25 | Oostende | 39.4 | [34.4;44.4] | 100.7 | ns. |
| 26 | Aalst | 40.2 | [36.2;44.2] | 102.7 | ns. |
| 27 | Marche-en-Famenne | 40.7 | [30.9;50.4] | 104.0 | ns. |
| 28 | Dendermonde | 41.0 | [36.1;45.8] | 105.0 | ns. |
| 29 | Diksmuide | 41.0 | [31.2;50.8] | 105.2 | ns. |
| 30 | Verviers | 42.3 | [38.0;46.6] | 108.6 | ns. |
| 31 | Neufchateau | 42.7 | [33.0;52.4] | 107.7 | ns. |
| 32 | Brussels | 43.3 | [40.9;45.7] | 111.0 | <0.001 |
| 33 | Tournai | 43.4 | [37.5;49.4] | 111.8 | ns. |
| 34 | Bastogne | 43.8 | [32.3;55.4] | 112.7 | ns. |
| 35 | Namur | 46.0 | [41.6;50.4] | 117.6 | <0.01 |
| 36 | Thuin | 46.1 | [40.1;52.1] | 117.9 | <0.05 |
| 37 | Dinant | 46.6 | [39.4;53.8] | 119.4 | <0.05 |
| 38 | Soignies | 47.0 | [41.4;52.7] | 119.6 | <0.01 |
| 39 | Li�ge | 48.1 | [45.1;51.1] | 122.2 | <0.001 |
| 40 | Waremme | 50.1 | [41.0;59.2] | 129.3 | <0.05 |
| 41 | Huy | 52.4 | [44.5;60.2] | 135.3 | <0.001 |
| 42 | Mons | 53.2 | [48.2;58.2] | 136.6 | <0.001 |
| 43 | Charleroi | 57.7 | [53.7;61.8] | 147.7 | <0.001 |

  

# Mean Rate = 39.0

# 

# \* p value of the z statistic testing for a the difference between the arrondissement's rate and the mean rate
