# Supplementary material for: Mapping the cause-specific premature mortality reveals large between-districts disparity in Belgium, 2003–2009
Source: Arch Public Health. 2015 Mar 23;73(1):13. doi: 10.1186/s13690-015-0060-5 (PMC4412101; doi:10.1186/s13690-015-0060-5)
Supplement: Additional file 30: Table S5. — Cerebrovasc.dis. &Hypertension Men 175. [file 13690_2015_60_MOESM30_ESM.zip › 13690_2015_60_MOESM30_ESM.html]

SAS Output


# Cerebrovasc.dis.&Hypertension Premature Mortality in Men (1-74 yr), Belgium 2003-2009

# Ranking of the arrondissements by increased mortality

# Age-adjusted rates per 100.000

| Rank | ARROND | Age-adj.Rates | CI on age-adj.Rates | smr | p value\* |
| --- | --- | --- | --- | --- | --- |
| 1 | Maaseik | 16.6 | [13.6;19.6] | 70.9 | <0.001 |
| 2 | Diksmuide | 17.1 | [10.6;23.5] | 75.6 | ns. |
| 3 | Veurne | 17.5 | [12.0;23.0] | 72.7 | ns. |
| 4 | Roeselare | 17.8 | [14.0;21.6] | 77.6 | <0.01 |
| 5 | Eeklo | 17.9 | [12.9;23.0] | 77.0 | ns. |
| 6 | Nivelles | 18.2 | [15.6;20.8] | 80.2 | <0.001 |
| 7 | Brugge | 18.4 | [15.7;21.1] | 79.8 | <0.01 |
| 8 | Turnhout | 18.7 | [16.3;21.0] | 80.6 | <0.001 |
| 9 | Leuven | 18.8 | [16.5;21.0] | 81.6 | <0.001 |
| 10 | Ieper | 18.9 | [14.2;23.6] | 81.8 | ns. |
| 11 | Kortrijk | 19.3 | [16.4;22.2] | 83.7 | <0.05 |
| 12 | Hasselt | 20.3 | [17.7;22.8] | 88.2 | <0.05 |
| 13 | Halle-Vilvoorde | 20.4 | [18.3;22.5] | 89.2 | <0.05 |
| 14 | Gent | 20.4 | [18.1;22.6] | 88.7 | <0.05 |
| 15 | Tielt | 21.0 | [15.7;26.3] | 93.2 | ns. |
| 16 | Tongeren | 22.0 | [18.3;25.7] | 94.3 | ns. |
| 17 | Mechelen | 22.0 | [19.1;25.0] | 96.1 | ns. |
| 18 | Oostende | 22.1 | [18.2;26.1] | 95.6 | ns. |
| 19 | Antwerpen | 22.4 | [20.7;24.1] | 97.6 | ns. |
| 20 | Aalst | 22.7 | [19.5;25.9] | 99.0 | ns. |
| 21 | Li�ge | 22.7 | [20.4;24.9] | 98.5 | ns. |
| 22 | Bastogne | 23.8 | [14.6;33.0] | 99.5 | ns. |
| 23 | Dendermonde | 24.0 | [20.1;27.9] | 104.9 | ns. |
| 24 | Namur | 24.1 | [20.6;27.6] | 103.0 | ns. |
| 25 | Verviers | 24.6 | [21.1;28.0] | 105.0 | ns. |
| 26 | Sint Niklaas | 24.8 | [21.1;28.4] | 107.5 | ns. |
| 27 | Brussels | 24.9 | [22.9;26.9] | 109.3 | ns. |
| 28 | Marche-en-Famenne | 25.7 | [17.5;33.8] | 113.2 | ns. |
| 29 | Waremme | 26.3 | [19.2;33.5] | 112.4 | ns. |
| 30 | Arlon | 26.4 | [18.0;34.7] | 115.4 | ns. |
| 31 | Oudenaarde | 26.4 | [21.1;31.6] | 114.4 | ns. |
| 32 | Mouscron | 26.8 | [19.7;34.0] | 116.4 | ns. |
| 33 | Virton | 27.2 | [18.6;35.8] | 122.6 | ns. |
| 34 | Soignies | 27.4 | [22.8;32.0] | 124.1 | ns. |
| 35 | Dinant | 27.8 | [21.9;33.6] | 123.3 | ns. |
| 36 | Huy | 27.9 | [21.7;34.2] | 118.3 | ns. |
| 37 | Neufchateau | 29.5 | [21.2;37.8] | 129.8 | ns. |
| 38 | Tournai | 32.7 | [27.1;38.3] | 146.3 | <0.001 |
| 39 | Mons | 33.7 | [29.3;38.2] | 149.6 | <0.001 |
| 40 | Thuin | 33.9 | [28.3;39.5] | 148.1 | <0.001 |
| 41 | Philippeville | 34.2 | [25.7;42.6] | 145.8 | <0.01 |
| 42 | Charleroi | 34.2 | [30.8;37.6] | 149.3 | <0.001 |
| 43 | Ath | 35.7 | [27.9;43.5] | 156.1 | <0.01 |

  

# Mean Rate = 22.9

# 

# \* p value of the z statistic testing for a the difference between the arrondissement's rate and the mean rate
