# Supplementary material for: Mapping the cause-specific premature mortality reveals large between-districts disparity in Belgium, 2003–2009
Source: Arch Public Health. 2015 Mar 23;73(1):13. doi: 10.1186/s13690-015-0060-5 (PMC4412101; doi:10.1186/s13690-015-0060-5)
Supplement: Additional file 31: Table S6. — Cerebrovasc. dis. & Hypertension Women 175. [file 13690_2015_60_MOESM31_ESM.zip › 13690_2015_60_MOESM31_ESM.html]

SAS Output


# Cerebrovasc.dis.&Hypertension Premature Mortality in Women (1-74 yr), Belgium 2003-2009

# Ranking of the arrondissements by increased mortality

# Age-adjusted rates per 100.000

| Rank | ARROND | Age-adj.Rates | CI on age-adj.Rates | smr | p value\* |
| --- | --- | --- | --- | --- | --- |
| 1 | Roeselare | 10.2 | [ 7.3;13.0] | 62.8 | <0.001 |
| 2 | Arlon | 10.5 | [ 5.5;15.6] | 65.1 | <0.05 |
| 3 | Tielt | 11.0 | [ 7.3;14.8] | 69.7 | <0.05 |
| 4 | Kortrijk | 11.3 | [ 9.2;13.4] | 71.1 | <0.001 |
| 5 | Brugge | 12.3 | [10.2;14.5] | 77.5 | <0.01 |
| 6 | Leuven | 12.5 | [10.8;14.2] | 79.5 | <0.001 |
| 7 | Nivelles | 12.8 | [10.7;14.9] | 80.4 | <0.01 |
| 8 | Virton | 12.9 | [ 7.1;18.7] | 77.8 | ns. |
| 9 | Verviers | 13.2 | [10.8;15.6] | 83.6 | <0.05 |
| 10 | Maaseik | 13.5 | [10.8;16.2] | 84.8 | ns. |
| 11 | Ieper | 14.0 | [10.1;17.8] | 90.1 | ns. |
| 12 | Halle-Vilvoorde | 14.4 | [12.7;16.1] | 90.6 | ns. |
| 13 | Gent | 14.4 | [12.6;16.2] | 91.6 | ns. |
| 14 | Huy | 14.5 | [10.4;18.6] | 92.9 | ns. |
| 15 | Veurne | 14.5 | [ 9.9;19.2] | 93.5 | ns. |
| 16 | Eeklo | 14.9 | [10.4;19.4] | 93.5 | ns. |
| 17 | Oudenaarde | 14.9 | [11.2;18.7] | 96.4 | ns. |
| 18 | Marche-en-Famenne | 15.2 | [ 9.2;21.1] | 95.5 | ns. |
| 19 | Turnhout | 15.2 | [13.2;17.3] | 96.2 | ns. |
| 20 | Waremme | 15.3 | [10.3;20.4] | 97.0 | ns. |
| 21 | Bastogne | 15.4 | [ 8.6;22.1] | 98.9 | ns. |
| 22 | Tongeren | 15.4 | [12.4;18.4] | 98.6 | ns. |
| 23 | Aalst | 15.5 | [13.0;18.0] | 97.4 | ns. |
| 24 | Mouscron | 15.7 | [10.7;20.7] | 100.6 | ns. |
| 25 | Oostende | 16.0 | [12.8;19.3] | 99.7 | ns. |
| 26 | Sint Niklaas | 16.0 | [13.2;18.8] | 101.7 | ns. |
| 27 | Hasselt | 16.2 | [14.0;18.4] | 101.4 | ns. |
| 28 | Namur | 16.5 | [13.9;19.1] | 104.2 | ns. |
| 29 | Thuin | 16.9 | [13.3;20.6] | 106.0 | ns. |
| 30 | Brussels | 17.1 | [15.6;18.6] | 108.1 | ns. |
| 31 | Mechelen | 17.2 | [14.7;19.6] | 109.3 | ns. |
| 32 | Antwerpen | 17.3 | [15.8;18.7] | 109.5 | ns. |
| 33 | Li�ge | 17.5 | [15.7;19.3] | 110.1 | ns. |
| 34 | Mons | 18.2 | [15.2;21.1] | 114.9 | ns. |
| 35 | Tournai | 18.3 | [14.4;22.2] | 115.7 | ns. |
| 36 | Soignies | 19.2 | [15.6;22.7] | 122.7 | ns. |
| 37 | Ath | 19.3 | [14.0;24.6] | 119.8 | ns. |
| 38 | Philippeville | 19.4 | [13.5;25.4] | 122.6 | ns. |
| 39 | Dinant | 19.4 | [14.8;24.1] | 122.7 | ns. |
| 40 | Diksmuide | 19.7 | [13.0;26.4] | 128.0 | ns. |
| 41 | Neufchateau | 19.8 | [13.2;26.3] | 123.7 | ns. |
| 42 | Dendermonde | 20.4 | [16.9;23.9] | 128.2 | <0.01 |
| 43 | Charleroi | 21.4 | [19.0;23.8] | 136.2 | <0.001 |

  

# Mean Rate = 15.8

# 

# \* p value of the z statistic testing for a the difference between the arrondissement's rate and the mean rate
