# Supplementary material for: Mapping the cause-specific premature mortality reveals large between-districts disparity in Belgium, 2003–2009
Source: Arch Public Health. 2015 Mar 23;73(1):13. doi: 10.1186/s13690-015-0060-5 (PMC4412101; doi:10.1186/s13690-015-0060-5)
Supplement: Additional file 32: Table S7. — Lung Ca Men 175. [file 13690_2015_60_MOESM32_ESM.zip › 13690_2015_60_MOESM32_ESM.html]

SAS Output


# Lung Ca Premature Mortality in Men (1-74 yr), Belgium 2003-2009

# Ranking of the arrondissements by increased mortality

# Age-adjusted rates per 100.000

| Rank | ARROND | Age-adj.Rates | CI on age-adj.Rates | smr | p value\* |
| --- | --- | --- | --- | --- | --- |
| 1 | Veurne | 47.7 | [39.1;56.4] | 74.4 | <0.001 |
| 2 | Nivelles | 53.0 | [48.6;57.5] | 81.2 | <0.001 |
| 3 | Halle-Vilvoorde | 55.4 | [52.0;58.9] | 84.7 | <0.001 |
| 4 | Antwerpen | 57.4 | [54.7;60.2] | 88.1 | <0.001 |
| 5 | Brugge | 57.7 | [53.0;62.5] | 88.7 | <0.01 |
| 6 | Leuven | 58.4 | [54.5;62.3] | 89.4 | <0.01 |
| 7 | Mechelen | 58.7 | [54.0;63.5] | 90.3 | <0.01 |
| 8 | Sint Niklaas | 59.5 | [53.9;65.1] | 91.6 | ns. |
| 9 | Eeklo | 59.5 | [50.4;68.5] | 91.7 | ns. |
| 10 | Brussels | 60.7 | [57.6;63.8] | 93.9 | <0.01 |
| 11 | Gent | 60.8 | [56.9;64.6] | 93.4 | <0.05 |
| 12 | Marche-en-Famenne | 60.9 | [48.5;73.3] | 95.8 | ns. |
| 13 | Maaseik | 62.7 | [56.9;68.5] | 95.5 | ns. |
| 14 | Tournai | 63.5 | [55.8;71.3] | 99.2 | ns. |
| 15 | Neufchateau | 63.8 | [51.7;76.0] | 99.8 | ns. |
| 16 | Verviers | 64.0 | [58.4;69.5] | 98.1 | ns. |
| 17 | Kortrijk | 64.1 | [58.9;69.4] | 98.4 | ns. |
| 18 | Oudenaarde | 64.2 | [56.0;72.4] | 98.8 | ns. |
| 19 | Roeselare | 64.9 | [57.7;72.2] | 100.0 | ns. |
| 20 | Turnhout | 65.2 | [60.9;69.5] | 99.4 | ns. |
| 21 | Tielt | 65.7 | [56.3;75.1] | 100.6 | ns. |
| 22 | Ieper | 65.9 | [57.1;74.6] | 101.6 | ns. |
| 23 | Waremme | 67.2 | [56.0;78.5] | 103.7 | ns. |
| 24 | Thuin | 67.7 | [59.9;75.4] | 106.5 | ns. |
| 25 | Mouscron | 68.7 | [57.4;80.1] | 104.8 | ns. |
| 26 | Oostende | 68.7 | [61.9;75.6] | 106.3 | ns. |
| 27 | Aalst | 69.2 | [63.7;74.8] | 106.5 | ns. |
| 28 | Diksmuide | 69.3 | [56.3;82.4] | 106.9 | ns. |
| 29 | Dendermonde | 69.6 | [62.9;76.2] | 106.4 | ns. |
| 30 | Arlon | 70.2 | [56.5;84.0] | 105.9 | ns. |
| 31 | Hasselt | 70.4 | [65.7;75.0] | 107.2 | <0.05 |
| 32 | Tongeren | 72.8 | [66.1;79.5] | 109.3 | <0.05 |
| 33 | Ath | 72.9 | [61.9;83.8] | 113.6 | ns. |
| 34 | Namur | 73.1 | [67.1;79.0] | 112.3 | <0.01 |
| 35 | Huy | 75.1 | [65.2;85.1] | 117.6 | <0.05 |
| 36 | Li�ge | 75.8 | [71.7;79.9] | 117.2 | <0.001 |
| 37 | Philippeville | 76.4 | [64.1;88.8] | 119.0 | ns. |
| 38 | Virton | 76.4 | [61.6;91.3] | 115.4 | ns. |
| 39 | Mons | 78.2 | [71.5;84.9] | 122.1 | <0.001 |
| 40 | Soignies | 78.3 | [70.5;86.1] | 120.3 | <0.001 |
| 41 | Dinant | 79.2 | [69.2;89.1] | 121.2 | <0.01 |
| 42 | Bastogne | 82.4 | [65.6;99.2] | 126.1 | <0.05 |
| 43 | Charleroi | 87.4 | [82.1;92.8] | 136.6 | <0.001 |

  

# Mean Rate = 65.1

# 

# \* p value of the z statistic testing for a the difference between the arrondissement's rate and the mean rate
