# Supplementary material for: Mapping the cause-specific premature mortality reveals large between-districts disparity in Belgium, 2003–2009
Source: Arch Public Health. 2015 Mar 23;73(1):13. doi: 10.1186/s13690-015-0060-5 (PMC4412101; doi:10.1186/s13690-015-0060-5)
Supplement: Additional file 33: Table S8. — Lung Ca Women 175. [file 13690_2015_60_MOESM33_ESM.zip › 13690_2015_60_MOESM33_ESM.html]

SAS Output


# Lung Ca Premature Mortality in Women (1-74 yr), Belgium 2003-2009

# Ranking of the arrondissements by increased mortality

# Age-adjusted rates per 100.000

| Rank | ARROND | Age-adj.Rates | CI on age-adj.Rates | smr | p value\* |
| --- | --- | --- | --- | --- | --- |
| 1 | Oudenaarde | 9.4 | [ 6.4;12.5] | 51.8 | <0.001 |
| 2 | Tielt | 9.5 | [ 5.9;13.0] | 53.0 | <0.001 |
| 3 | Kortrijk | 9.8 | [ 7.9;11.8] | 54.6 | <0.001 |
| 4 | Ieper | 9.9 | [ 6.6;13.1] | 55.3 | <0.001 |
| 5 | Diksmuide | 11.3 | [ 6.1;16.6] | 62.8 | <0.05 |
| 6 | Tournai | 11.4 | [ 8.4;14.5] | 63.4 | <0.001 |
| 7 | Dendermonde | 11.5 | [ 8.9;14.1] | 64.0 | <0.001 |
| 8 | Roeselare | 11.5 | [ 8.5;14.6] | 63.1 | <0.001 |
| 9 | Veurne | 11.8 | [ 7.6;16.0] | 67.7 | <0.01 |
| 10 | Brugge | 12.2 | [10.1;14.3] | 68.4 | <0.001 |
| 11 | Leuven | 12.9 | [11.2;14.7] | 72.5 | <0.001 |
| 12 | Aalst | 13.1 | [10.8;15.4] | 73.1 | <0.001 |
| 13 | Eeklo | 13.8 | [ 9.5;18.2] | 75.8 | ns. |
| 14 | Mouscron | 14.4 | [ 9.5;19.4] | 76.4 | ns. |
| 15 | Neufchateau | 14.5 | [ 8.9;20.1] | 81.1 | ns. |
| 16 | Halle-Vilvoorde | 14.6 | [13.0;16.3] | 81.9 | <0.001 |
| 17 | Gent | 15.0 | [13.1;16.8] | 83.5 | <0.01 |
| 18 | Soignies | 15.0 | [11.9;18.2] | 84.9 | ns. |
| 19 | Maaseik | 15.1 | [12.3;17.9] | 84.4 | ns. |
| 20 | Philippeville | 15.9 | [10.5;21.3] | 87.7 | ns. |
| 21 | Sint Niklaas | 16.1 | [13.3;19.0] | 90.5 | ns. |
| 22 | Mons | 16.7 | [13.9;19.5] | 93.3 | ns. |
| 23 | Mechelen | 17.3 | [14.8;19.8] | 96.6 | ns. |
| 24 | Thuin | 17.3 | [13.7;21.0] | 96.9 | ns. |
| 25 | Tongeren | 17.4 | [14.2;20.5] | 96.2 | ns. |
| 26 | Oostende | 17.6 | [14.2;21.1] | 95.5 | ns. |
| 27 | Arlon | 17.6 | [11.3;24.0] | 100.9 | ns. |
| 28 | Nivelles | 18.0 | [15.6;20.4] | 100.2 | ns. |
| 29 | Bastogne | 18.3 | [10.8;25.8] | 101.0 | ns. |
| 30 | Turnhout | 18.7 | [16.5;21.0] | 104.5 | ns. |
| 31 | Hasselt | 18.7 | [16.4;21.0] | 104.5 | ns. |
| 32 | Charleroi | 20.7 | [18.3;23.1] | 116.1 | <0.05 |
| 33 | Antwerpen | 21.0 | [19.4;22.6] | 117.8 | <0.001 |
| 34 | Waremme | 21.0 | [15.1;26.9] | 116.8 | ns. |
| 35 | Virton | 21.3 | [13.9;28.8] | 117.2 | ns. |
| 36 | Namur | 22.0 | [19.0;24.9] | 124.8 | <0.01 |
| 37 | Ath | 22.3 | [16.6;27.9] | 122.9 | ns. |
| 38 | Marche-en-Famenne | 23.0 | [15.7;30.2] | 130.2 | ns. |
| 39 | Brussels | 23.3 | [21.5;25.0] | 129.6 | <0.001 |
| 40 | Huy | 24.0 | [18.6;29.3] | 130.9 | <0.05 |
| 41 | Verviers | 24.0 | [20.8;27.2] | 134.6 | <0.001 |
| 42 | Dinant | 24.5 | [19.3;29.7] | 136.9 | <0.05 |
| 43 | Li�ge | 28.6 | [26.2;30.9] | 157.5 | <0.001 |

  

# Mean Rate = 17.9

# 

# \* p value of the z statistic testing for a the difference between the arrondissement's rate and the mean rate
