# Supplementary material for: Mapping the cause-specific premature mortality reveals large between-districts disparity in Belgium, 2003–2009
Source: Arch Public Health. 2015 Mar 23;73(1):13. doi: 10.1186/s13690-015-0060-5 (PMC4412101; doi:10.1186/s13690-015-0060-5)
Supplement: Additional file 34: Table S9. — Lip, Oral Cavity, Pharynx, Larynx & oesoph. Ca Men 175. [file 13690_2015_60_MOESM34_ESM.zip › 13690_2015_60_MOESM34_ESM.html]

SAS Output


# Lip, Oral Cavity,Pharynx, Larynx & oesoph.Ca Premature Mortality in Men (1-74 yr), Belgium 2003-2009

# Ranking of the arrondissements by increased mortality

# Age-adjusted rates per 100.000

| Rank | ARROND | Age-adj.Rates | CI on age-adj.Rates | smr | p value\* |
| --- | --- | --- | --- | --- | --- |
| 1 | Turnhout | 9.3 | [ 7.7;10.8] | 53.5 | <0.001 |
| 2 | Maaseik | 9.5 | [ 7.3;11.7] | 53.9 | <0.001 |
| 3 | Huy | 10.8 | [ 7.0;14.6] | 58.4 | <0.01 |
| 4 | Tongeren | 11.0 | [ 8.5;13.6] | 62.2 | <0.001 |
| 5 | Hasselt | 11.1 | [ 9.2;12.9] | 63.7 | <0.001 |
| 6 | Mechelen | 13.2 | [11.0;15.5] | 76.5 | <0.001 |
| 7 | Verviers | 13.7 | [11.2;16.2] | 80.9 | <0.01 |
| 8 | Leuven | 14.6 | [12.7;16.6] | 83.4 | <0.05 |
| 9 | Arlon | 14.9 | [ 8.7;21.0] | 85.6 | ns. |
| 10 | Antwerpen | 14.9 | [13.5;16.3] | 86.3 | <0.01 |
| 11 | Eeklo | 15.0 | [10.5;19.5] | 88.1 | ns. |
| 12 | Soignies | 15.2 | [11.8;18.5] | 88.1 | ns. |
| 13 | Li�ge | 15.7 | [13.9;17.5] | 91.2 | ns. |
| 14 | Nivelles | 15.7 | [13.3;18.1] | 90.3 | ns. |
| 15 | Philippeville | 16.8 | [11.3;22.2] | 104.3 | ns. |
| 16 | Roeselare | 16.8 | [13.1;20.4] | 98.0 | ns. |
| 17 | Bastogne | 16.8 | [ 9.5;24.1] | 100.7 | ns. |
| 18 | Sint Niklaas | 16.9 | [13.9;19.8] | 96.7 | ns. |
| 19 | Waremme | 17.2 | [11.5;22.9] | 93.7 | ns. |
| 20 | Namur | 17.2 | [14.5;20.0] | 102.4 | ns. |
| 21 | Halle-Vilvoorde | 17.4 | [15.5;19.3] | 100.8 | ns. |
| 22 | Brugge | 17.6 | [15.0;20.2] | 102.9 | ns. |
| 23 | Diksmuide | 17.7 | [11.1;24.3] | 101.8 | ns. |
| 24 | Kortrijk | 18.0 | [15.3;20.8] | 105.5 | ns. |
| 25 | Marche-en-Famenne | 18.2 | [11.5;24.9] | 104.4 | ns. |
| 26 | Tielt | 18.6 | [13.7;23.5] | 109.0 | ns. |
| 27 | Veurne | 18.8 | [13.2;24.3] | 109.0 | ns. |
| 28 | Brussels | 19.1 | [17.4;20.8] | 108.8 | <0.05 |
| 29 | Ieper | 19.7 | [15.0;24.4] | 116.7 | ns. |
| 30 | Gent | 20.1 | [17.9;22.2] | 115.5 | <0.05 |
| 31 | Dinant | 20.3 | [15.4;25.2] | 117.9 | ns. |
| 32 | Thuin | 20.6 | [16.5;24.8] | 122.2 | ns. |
| 33 | Neufchateau | 21.1 | [14.2;27.9] | 123.4 | ns. |
| 34 | Dendermonde | 22.3 | [18.6;26.0] | 131.4 | <0.01 |
| 35 | Virton | 22.4 | [14.5;30.2] | 128.5 | ns. |
| 36 | Charleroi | 22.6 | [20.0;25.3] | 131.1 | <0.001 |
| 37 | Oostende | 24.0 | [20.0;28.1] | 140.5 | <0.01 |
| 38 | Oudenaarde | 24.2 | [19.2;29.1] | 141.5 | <0.01 |
| 39 | Aalst | 25.6 | [22.3;28.9] | 149.5 | <0.001 |
| 40 | Mons | 26.4 | [22.7;30.1] | 158.9 | <0.001 |
| 41 | Mouscron | 26.6 | [19.8;33.5] | 156.1 | <0.01 |
| 42 | Ath | 27.5 | [21.1;34.0] | 167.0 | <0.01 |
| 43 | Tournai | 29.1 | [24.0;34.2] | 172.4 | <0.001 |

  

# Mean Rate = 17.2

# 

# \* p value of the z statistic testing for a the difference between the arrondissement's rate and the mean rate
