# Supplementary material for: Mapping the cause-specific premature mortality reveals large between-districts disparity in Belgium, 2003–2009
Source: Arch Public Health. 2015 Mar 23;73(1):13. doi: 10.1186/s13690-015-0060-5 (PMC4412101; doi:10.1186/s13690-015-0060-5)
Supplement: Additional file 35: Table S10. — Lip, Oral Cavity, Pharynx, Larynx & oesoph. Ca Women 175. [file 13690_2015_60_MOESM35_ESM.zip › 13690_2015_60_MOESM35_ESM.html]

SAS Output


# Lip, Oral Cavity,Pharynx, Larynx & oesoph.Ca Premature Mortality in Women (1-74 yr), Belgium 2003-2009

# Ranking of the arrondissements by increased mortality

# Age-adjusted rates per 100.000

| Rank | ARROND | Age-adj.Rates | CI on age-adj.Rates | smr | p value\* |
| --- | --- | --- | --- | --- | --- |
| 1 | Ieper | 1.4 | [ 0.2; 2.7] | 38.3 | <0.001 |
| 2 | Roeselare | 2.0 | [ 0.8; 3.3] | 54.7 | <0.05 |
| 3 | Turnhout | 2.1 | [ 1.4; 2.9] | 57.4 | <0.001 |
| 4 | Tongeren | 2.3 | [ 1.1; 3.5] | 60.5 | <0.05 |
| 5 | Sint Niklaas | 2.3 | [ 1.3; 3.4] | 61.7 | <0.05 |
| 6 | Brugge | 2.4 | [ 1.4; 3.3] | 64.8 | <0.01 |
| 7 | Hasselt | 2.4 | [ 1.6; 3.2] | 64.2 | <0.01 |
| 8 | Eeklo | 2.4 | [ 0.6; 4.2] | 66.0 | ns. |
| 9 | Thuin | 2.5 | [ 1.1; 3.9] | 69.6 | ns. |
| 10 | Maaseik | 2.6 | [ 1.5; 3.8] | 72.3 | ns. |
| 11 | Verviers | 2.8 | [ 1.7; 3.9] | 75.8 | ns. |
| 12 | Halle-Vilvoorde | 3.0 | [ 2.2; 3.7] | 82.2 | ns. |
| 13 | Diksmuide | 3.0 | [ 0.4; 5.7] | 84.8 | ns. |
| 14 | Mechelen | 3.1 | [ 2.0; 4.1] | 82.5 | ns. |
| 15 | Tielt | 3.2 | [ 1.1; 5.2] | 82.9 | ns. |
| 16 | Leuven | 3.2 | [ 2.3; 4.1] | 86.5 | ns. |
| 17 | Oudenaarde | 3.2 | [ 1.4; 4.9] | 88.4 | ns. |
| 18 | Nivelles | 3.3 | [ 2.3; 4.3] | 90.3 | ns. |
| 19 | Tournai | 3.4 | [ 1.7; 5.1] | 91.2 | ns. |
| 20 | Aalst | 3.4 | [ 2.3; 4.6] | 96.7 | ns. |
| 21 | Dendermonde | 3.5 | [ 2.1; 4.9] | 94.2 | ns. |
| 22 | Virton | 3.7 | [ 0.7; 6.6] | 107.4 | ns. |
| 23 | Gent | 3.7 | [ 2.8; 4.6] | 102.2 | ns. |
| 24 | Philippeville | 3.7 | [ 1.1; 6.4] | 100.2 | ns. |
| 25 | Antwerpen | 3.8 | [ 3.1; 4.4] | 102.8 | ns. |
| 26 | Bastogne | 3.8 | [ 0.5; 7.2] | 107.0 | ns. |
| 27 | Namur | 4.4 | [ 3.0; 5.7] | 122.6 | ns. |
| 28 | Neufchateau | 4.4 | [ 1.3; 7.5] | 121.4 | ns. |
| 29 | Veurne | 4.4 | [ 1.9; 7.0] | 126.1 | ns. |
| 30 | Dinant | 4.5 | [ 2.2; 6.7] | 117.4 | ns. |
| 31 | Kortrijk | 4.5 | [ 3.1; 5.8] | 119.9 | ns. |
| 32 | Oostende | 4.5 | [ 2.8; 6.2] | 121.0 | ns. |
| 33 | Li�ge | 4.6 | [ 3.7; 5.5] | 125.7 | ns. |
| 34 | Mons | 4.7 | [ 3.2; 6.2] | 128.6 | ns. |
| 35 | Huy | 4.7 | [ 2.4; 7.0] | 129.2 | ns. |
| 36 | Charleroi | 4.7 | [ 3.6; 5.9] | 127.7 | ns. |
| 37 | Soignies | 5.2 | [ 3.4; 7.1] | 142.1 | ns. |
| 38 | Brussels | 5.3 | [ 4.4; 6.1] | 141.7 | <0.001 |
| 39 | Arlon | 5.3 | [ 1.8; 8.8] | 147.5 | ns. |
| 40 | Mouscron | 5.4 | [ 2.4; 8.3] | 145.8 | ns. |
| 41 | Ath | 5.5 | [ 2.7; 8.3] | 149.3 | ns. |
| 42 | Waremme | 6.3 | [ 3.1; 9.6] | 170.6 | ns. |
| 43 | Marche-en-Famenne | 7.0 | [ 3.0;11.0] | 195.0 | ns. |

  

# Mean Rate = 3.7

# 

# \* p value of the z statistic testing for a the difference between the arrondissement's rate and the mean rate
