# Supplementary material for: Mapping the cause-specific premature mortality reveals large between-districts disparity in Belgium, 2003–2009
Source: Arch Public Health. 2015 Mar 23;73(1):13. doi: 10.1186/s13690-015-0060-5 (PMC4412101; doi:10.1186/s13690-015-0060-5)
Supplement: Additional file 36: Table S11. — Breast Ca Women 175. [file 13690_2015_60_MOESM36_ESM.zip › 13690_2015_60_MOESM36_ESM.html]

SAS Output


# Breast Ca Premature Mortality in Women (1-74 yr), Belgium 2003-2009

# Ranking of the arrondissements by increased mortality

# Age-adjusted rates per 100.000

| Rank | ARROND | Age-adj.Rates | CI on age-adj.Rates | smr | p value\* |
| --- | --- | --- | --- | --- | --- |
| 1 | Marche-en-Famenne | 19.6 | [12.9;26.3] | 75.7 | ns. |
| 2 | Arlon | 19.8 | [13.0;26.5] | 75.6 | ns. |
| 3 | Veurne | 20.1 | [14.4;25.8] | 76.9 | <0.05 |
| 4 | Verviers | 21.6 | [18.6;24.7] | 82.7 | <0.01 |
| 5 | Maaseik | 21.8 | [18.5;25.1] | 83.7 | <0.05 |
| 6 | Namur | 22.2 | [19.2;25.2] | 85.0 | <0.05 |
| 7 | Dinant | 22.7 | [17.7;27.8] | 86.7 | ns. |
| 8 | Thuin | 23.1 | [18.9;27.3] | 89.6 | ns. |
| 9 | Oudenaarde | 23.3 | [18.6;28.1] | 89.7 | ns. |
| 10 | Turnhout | 23.3 | [20.8;25.9] | 88.6 | <0.05 |
| 11 | Huy | 23.4 | [18.2;28.6] | 88.6 | ns. |
| 12 | Li�ge | 23.9 | [21.8;26.0] | 90.9 | <0.05 |
| 13 | Waremme | 24.4 | [18.2;30.5] | 95.9 | ns. |
| 14 | Neufchateau | 24.6 | [17.3;31.9] | 94.2 | ns. |
| 15 | Nivelles | 24.8 | [21.9;27.6] | 93.5 | ns. |
| 16 | Sint Niklaas | 24.8 | [21.3;28.3] | 95.0 | ns. |
| 17 | Hasselt | 24.8 | [22.1;27.5] | 94.8 | ns. |
| 18 | Mons | 25.3 | [21.9;28.7] | 98.4 | ns. |
| 19 | Philippeville | 25.9 | [19.1;32.7] | 101.5 | ns. |
| 20 | Leuven | 26.1 | [23.6;28.6] | 100.2 | ns. |
| 21 | Oostende | 26.2 | [22.0;30.4] | 98.6 | ns. |
| 22 | Tournai | 26.5 | [21.8;31.2] | 101.1 | ns. |
| 23 | Halle-Vilvoorde | 26.7 | [24.4;28.9] | 102.0 | ns. |
| 24 | Soignies | 26.7 | [22.5;30.9] | 101.0 | ns. |
| 25 | Bastogne | 26.8 | [17.8;35.9] | 102.1 | ns. |
| 26 | Antwerpen | 26.8 | [25.0;28.6] | 102.8 | ns. |
| 27 | Gent | 26.9 | [24.4;29.3] | 102.1 | ns. |
| 28 | Charleroi | 27.1 | [24.3;29.8] | 104.1 | ns. |
| 29 | Tielt | 27.2 | [21.2;33.2] | 103.1 | ns. |
| 30 | Brugge | 27.2 | [24.0;30.4] | 103.3 | ns. |
| 31 | Mouscron | 27.2 | [20.5;34.0] | 102.5 | ns. |
| 32 | Tongeren | 27.3 | [23.3;31.3] | 104.4 | ns. |
| 33 | Brussels | 27.3 | [25.4;29.2] | 104.7 | ns. |
| 34 | Virton | 27.3 | [18.9;35.8] | 102.7 | ns. |
| 35 | Mechelen | 27.7 | [24.6;30.8] | 106.4 | ns. |
| 36 | Ieper | 28.6 | [23.0;34.3] | 108.2 | ns. |
| 37 | Aalst | 28.7 | [25.3;32.1] | 109.7 | ns. |
| 38 | Roeselare | 29.7 | [24.9;34.5] | 114.5 | ns. |
| 39 | Ath | 30.2 | [23.6;36.8] | 115.5 | ns. |
| 40 | Eeklo | 31.1 | [24.6;37.7] | 118.3 | ns. |
| 41 | Kortrijk | 32.5 | [28.9;36.1] | 124.4 | <0.001 |
| 42 | Dendermonde | 34.2 | [29.7;38.7] | 130.6 | <0.001 |
| 43 | Diksmuide | 35.2 | [26.0;44.5] | 134.6 | ns. |

  

# Mean Rate = 26.2

# 

# \* p value of the z statistic testing for a the difference between the arrondissement's rate and the mean rate
