# Supplementary material for: Mapping the cause-specific premature mortality reveals large between-districts disparity in Belgium, 2003–2009
Source: Arch Public Health. 2015 Mar 23;73(1):13. doi: 10.1186/s13690-015-0060-5 (PMC4412101; doi:10.1186/s13690-015-0060-5)
Supplement: Additional file 37: Table S12. — Colorectal Ca Men 175. [file 13690_2015_60_MOESM37_ESM.zip › 13690_2015_60_MOESM37_ESM.html]

SAS Output


# Colorectal Ca Premature Mortality in Men (1-74 yr), Belgium 2003-2009

# Ranking of the arrondissements by increased mortality

# Age-adjusted rates per 100.000

| Rank | ARROND | Age-adj.Rates | CI on age-adj.Rates | smr | p value\* |
| --- | --- | --- | --- | --- | --- |
| 1 | Marche-en-Famenne | 9.5 | [ 4.5;14.5] | 58.4 | <0.05 |
| 2 | Virton | 9.8 | [ 4.6;15.0] | 63.8 | <0.05 |
| 3 | Verviers | 11.9 | [ 9.6;14.3] | 76.2 | <0.01 |
| 4 | Dinant | 12.2 | [ 8.3;16.1] | 77.3 | ns. |
| 5 | Veurne | 12.3 | [ 7.9;16.7] | 77.5 | ns. |
| 6 | Eeklo | 13.0 | [ 8.8;17.3] | 81.1 | ns. |
| 7 | Diksmuide | 13.4 | [ 7.7;19.2] | 84.5 | ns. |
| 8 | Tongeren | 14.1 | [11.2;17.0] | 89.2 | ns. |
| 9 | Turnhout | 14.2 | [12.2;16.2] | 88.0 | ns. |
| 10 | Mouscron | 14.2 | [ 9.1;19.2] | 93.9 | ns. |
| 11 | Arlon | 14.2 | [ 8.1;20.4] | 89.8 | ns. |
| 12 | Neufchateau | 14.3 | [ 8.5;20.1] | 91.5 | ns. |
| 13 | Sint Niklaas | 14.3 | [11.6;17.1] | 89.6 | ns. |
| 14 | Hasselt | 14.6 | [12.4;16.7] | 91.7 | ns. |
| 15 | Oostende | 14.6 | [11.4;17.7] | 92.8 | ns. |
| 16 | Tielt | 14.6 | [10.2;19.1] | 92.2 | ns. |
| 17 | Li�ge | 14.9 | [13.1;16.7] | 94.0 | ns. |
| 18 | Brugge | 15.0 | [12.5;17.4] | 93.2 | ns. |
| 19 | Brussels | 15.4 | [13.8;16.9] | 96.8 | ns. |
| 20 | Leuven | 15.4 | [13.4;17.4] | 96.7 | ns. |
| 21 | Nivelles | 15.5 | [13.0;17.9] | 96.6 | ns. |
| 22 | Namur | 15.6 | [12.8;18.4] | 96.2 | ns. |
| 23 | Ieper | 15.8 | [11.5;20.1] | 98.4 | ns. |
| 24 | Huy | 15.9 | [11.3;20.5] | 101.1 | ns. |
| 25 | Tournai | 15.9 | [12.0;19.8] | 102.6 | ns. |
| 26 | Mechelen | 16.0 | [13.6;18.5] | 100.6 | ns. |
| 27 | Philippeville | 16.2 | [10.5;21.8] | 104.5 | ns. |
| 28 | Maaseik | 16.2 | [13.2;19.1] | 101.5 | ns. |
| 29 | Charleroi | 16.4 | [14.1;18.8] | 104.1 | ns. |
| 30 | Soignies | 16.5 | [12.9;20.1] | 103.4 | ns. |
| 31 | Antwerpen | 16.5 | [15.1;18.0] | 102.8 | ns. |
| 32 | Halle-Vilvoorde | 16.6 | [14.7;18.5] | 104.0 | ns. |
| 33 | Roeselare | 16.7 | [13.0;20.4] | 104.7 | ns. |
| 34 | Gent | 17.3 | [15.3;19.4] | 108.2 | ns. |
| 35 | Mons | 17.4 | [14.2;20.6] | 109.7 | ns. |
| 36 | Bastogne | 17.8 | [10.1;25.5] | 116.1 | ns. |
| 37 | Oudenaarde | 18.1 | [13.7;22.4] | 112.1 | ns. |
| 38 | Aalst | 18.3 | [15.5;21.2] | 115.0 | ns. |
| 39 | Waremme | 19.5 | [13.3;25.7] | 118.9 | ns. |
| 40 | Kortrijk | 19.7 | [16.8;22.6] | 123.6 | <0.05 |
| 41 | Ath | 20.6 | [14.8;26.5] | 132.5 | ns. |
| 42 | Thuin | 21.0 | [16.6;25.4] | 130.8 | <0.05 |
| 43 | Dendermonde | 23.1 | [19.3;27.0] | 144.0 | <0.001 |

  

# Mean Rate = 15.9

# 

# \* p value of the z statistic testing for a the difference between the arrondissement's rate and the mean rate
