# Supplementary material for: Mapping the cause-specific premature mortality reveals large between-districts disparity in Belgium, 2003–2009
Source: Arch Public Health. 2015 Mar 23;73(1):13. doi: 10.1186/s13690-015-0060-5 (PMC4412101; doi:10.1186/s13690-015-0060-5)
Supplement: Additional file 38: Table S13. — Colorectal Ca Women 175. [file 13690_2015_60_MOESM38_ESM.zip › 13690_2015_60_MOESM38_ESM.html]

SAS Output


# Colorectal Ca Premature Mortality in Women (1-74 yr), Belgium 2003-2009

# Ranking of the arrondissements by increased mortality

# Age-adjusted rates per 100.000

| Rank | ARROND | Age-adj.Rates | CI on age-adj.Rates | smr | p value\* |
| --- | --- | --- | --- | --- | --- |
| 1 | Bastogne | 4.2 | [ 0.5; 7.8] | 39.7 | <0.01 |
| 2 | Eeklo | 5.6 | [ 2.8; 8.4] | 55.4 | <0.01 |
| 3 | Waremme | 6.1 | [ 2.9; 9.4] | 60.0 | <0.05 |
| 4 | Verviers | 7.3 | [ 5.5; 9.1] | 72.6 | <0.01 |
| 5 | Ath | 7.4 | [ 4.1;10.6] | 74.7 | ns. |
| 6 | Philippeville | 7.8 | [ 4.1;11.6] | 80.3 | ns. |
| 7 | Li�ge | 7.9 | [ 6.6; 9.1] | 79.1 | <0.01 |
| 8 | Namur | 8.3 | [ 6.4;10.1] | 83.9 | ns. |
| 9 | Leuven | 8.7 | [ 7.3;10.2] | 86.4 | ns. |
| 10 | Oostende | 8.9 | [ 6.6;11.3] | 91.3 | ns. |
| 11 | Oudenaarde | 9.0 | [ 6.1;11.9] | 92.2 | ns. |
| 12 | Mechelen | 9.0 | [ 7.2;10.8] | 89.8 | ns. |
| 13 | Nivelles | 9.3 | [ 7.6;11.1] | 92.3 | ns. |
| 14 | Huy | 9.4 | [ 6.0;12.8] | 92.1 | ns. |
| 15 | Neufchateau | 9.4 | [ 4.9;13.9] | 96.1 | ns. |
| 16 | Brugge | 9.4 | [ 7.6;11.3] | 94.8 | ns. |
| 17 | Halle-Vilvoorde | 9.5 | [ 8.1;10.9] | 95.2 | ns. |
| 18 | Kortrijk | 9.6 | [ 7.7;11.6] | 96.7 | ns. |
| 19 | Turnhout | 9.7 | [ 8.1;11.3] | 98.1 | ns. |
| 20 | Mouscron | 9.9 | [ 5.9;13.9] | 100.9 | ns. |
| 21 | Gent | 10.0 | [ 8.5;11.5] | 100.6 | ns. |
| 22 | Thuin | 10.2 | [ 7.3;13.0] | 99.1 | ns. |
| 23 | Arlon | 10.2 | [ 5.3;15.1] | 104.1 | ns. |
| 24 | Maaseik | 10.3 | [ 8.0;12.6] | 104.0 | ns. |
| 25 | Dendermonde | 10.6 | [ 8.1;13.0] | 106.6 | ns. |
| 26 | Brussels | 10.6 | [ 9.4;11.8] | 106.8 | ns. |
| 27 | Antwerpen | 10.6 | [ 9.5;11.7] | 106.8 | ns. |
| 28 | Tongeren | 10.6 | [ 8.1;13.1] | 108.3 | ns. |
| 29 | Sint Niklaas | 10.8 | [ 8.5;13.1] | 107.9 | ns. |
| 30 | Virton | 10.8 | [ 5.5;16.1] | 105.7 | ns. |
| 31 | Veurne | 10.9 | [ 6.9;14.9] | 111.9 | ns. |
| 32 | Aalst | 11.0 | [ 8.9;13.1] | 112.9 | ns. |
| 33 | Hasselt | 11.1 | [ 9.3;12.9] | 110.8 | ns. |
| 34 | Roeselare | 11.1 | [ 8.2;14.0] | 112.0 | ns. |
| 35 | Charleroi | 11.2 | [ 9.4;12.9] | 112.0 | ns. |
| 36 | Ieper | 11.8 | [ 8.2;15.4] | 118.4 | ns. |
| 37 | Tournai | 12.2 | [ 9.0;15.3] | 124.2 | ns. |
| 38 | Diksmuide | 12.3 | [ 6.9;17.8] | 124.1 | ns. |
| 39 | Dinant | 12.4 | [ 8.6;16.2] | 122.4 | ns. |
| 40 | Marche-en-Famenne | 12.4 | [ 7.0;17.9] | 121.8 | ns. |
| 41 | Mons | 12.6 | [10.2;15.1] | 127.1 | <0.05 |
| 42 | Tielt | 12.7 | [ 8.6;16.7] | 128.3 | ns. |
| 43 | Soignies | 12.9 | [ 9.9;15.8] | 130.3 | ns. |

  

# Mean Rate = 10.0

# 

# \* p value of the z statistic testing for a the difference between the arrondissement's rate and the mean rate
