# Supplementary material for: Mapping the cause-specific premature mortality reveals large between-districts disparity in Belgium, 2003–2009
Source: Arch Public Health. 2015 Mar 23;73(1):13. doi: 10.1186/s13690-015-0060-5 (PMC4412101; doi:10.1186/s13690-015-0060-5)
Supplement: Additional file 39: Table S14. — Diabete (analysis in multiple causes) Men 175. [file 13690_2015_60_MOESM39_ESM.zip › 13690_2015_60_MOESM39_ESM.html]

SAS Output


# Diabete(analysis in multiple causes) Premature Mortality in Men (1-74 yr), Belgium 2003-2009

# Ranking of the arrondissements by increased mortality

# Age-adjusted rates per 100.000

| Rank | ARROND | Age-adj.Rates | CI on age-adj.Rates | smr | p value\* |
| --- | --- | --- | --- | --- | --- |
| 1 | Turnhout | 13.4 | [11.5;15.4] | 52.2 | <0.001 |
| 2 | Hasselt | 14.1 | [12.1;16.2] | 55.8 | <0.001 |
| 3 | Tongeren | 14.6 | [11.5;17.6] | 56.1 | <0.001 |
| 4 | Oostende | 15.4 | [12.1;18.6] | 60.6 | <0.001 |
| 5 | Veurne | 15.6 | [10.5;20.7] | 59.6 | <0.001 |
| 6 | Maaseik | 15.6 | [12.7;18.6] | 58.3 | <0.001 |
| 7 | Antwerpen | 16.2 | [14.7;17.6] | 62.6 | <0.001 |
| 8 | Sint Niklaas | 16.4 | [13.4;19.3] | 64.8 | <0.001 |
| 9 | Leuven | 16.6 | [14.5;18.7] | 64.6 | <0.001 |
| 10 | Kortrijk | 18.0 | [15.2;20.8] | 70.3 | <0.001 |
| 11 | Gent | 18.1 | [16.0;20.2] | 70.2 | <0.001 |
| 12 | Mechelen | 18.8 | [16.1;21.5] | 72.6 | <0.001 |
| 13 | Brugge | 19.1 | [16.4;21.9] | 75.0 | <0.001 |
| 14 | Eeklo | 19.6 | [14.4;24.8] | 75.7 | <0.05 |
| 15 | Oudenaarde | 20.5 | [15.9;25.1] | 81.2 | <0.05 |
| 16 | Halle-Vilvoorde | 22.1 | [19.9;24.3] | 85.7 | <0.01 |
| 17 | Dendermonde | 22.7 | [18.9;26.5] | 87.6 | ns. |
| 18 | Roeselare | 22.7 | [18.4;27.1] | 88.2 | ns. |
| 19 | Ieper | 23.5 | [18.3;28.7] | 91.8 | ns. |
| 20 | Tielt | 23.8 | [18.1;29.5] | 91.5 | ns. |
| 21 | Neufchateau | 24.7 | [17.0;32.5] | 94.7 | ns. |
| 22 | Aalst | 25.0 | [21.7;28.3] | 97.9 | ns. |
| 23 | Diksmuide | 26.1 | [18.1;34.1] | 102.6 | ns. |
| 24 | Nivelles | 29.7 | [26.3;33.0] | 116.1 | <0.05 |
| 25 | Li�ge | 30.2 | [27.6;32.8] | 117.7 | <0.001 |
| 26 | Verviers | 31.0 | [27.1;34.9] | 119.8 | <0.01 |
| 27 | Huy | 31.2 | [24.8;37.7] | 124.2 | ns. |
| 28 | Marche-en-Famenne | 32.1 | [23.1;41.2] | 127.0 | ns. |
| 29 | Arlon | 33.3 | [23.9;42.7] | 130.1 | ns. |
| 30 | Dinant | 33.6 | [27.1;40.1] | 131.4 | <0.05 |
| 31 | Waremme | 33.9 | [25.9;41.9] | 134.4 | <0.05 |
| 32 | Virton | 34.7 | [24.9;44.5] | 138.5 | ns. |
| 33 | Namur | 35.0 | [30.9;39.2] | 139.0 | <0.001 |
| 34 | Philippeville | 35.3 | [26.9;43.7] | 142.0 | <0.05 |
| 35 | Brussels | 39.0 | [36.5;41.5] | 150.2 | <0.001 |
| 36 | Soignies | 43.4 | [37.6;49.2] | 171.6 | <0.001 |
| 37 | Bastogne | 44.0 | [31.8;56.3] | 175.1 | <0.01 |
| 38 | Thuin | 44.4 | [38.1;50.7] | 175.2 | <0.001 |
| 39 | Tournai | 45.2 | [38.6;51.8] | 178.6 | <0.001 |
| 40 | Charleroi | 47.2 | [43.3;51.2] | 187.4 | <0.001 |
| 41 | Ath | 48.1 | [39.0;57.1] | 188.0 | <0.001 |
| 42 | Mons | 51.7 | [46.2;57.2] | 203.2 | <0.001 |
| 43 | Mouscron | 54.9 | [44.7;65.1] | 212.9 | <0.001 |

  

# Mean Rate = 25.6

# 

# \* p value of the z statistic testing for a the difference between the arrondissement's rate and the mean rate
