# Supplementary material for: Mapping the cause-specific premature mortality reveals large between-districts disparity in Belgium, 2003–2009
Source: Arch Public Health. 2015 Mar 23;73(1):13. doi: 10.1186/s13690-015-0060-5 (PMC4412101; doi:10.1186/s13690-015-0060-5)
Supplement: Additional file 40: Table S15. — Diabete (analysis in multiple causes) Women 175. [file 13690_2015_60_MOESM40_ESM.zip › 13690_2015_60_MOESM40_ESM.html]

SAS Output


# Diabete(analysis in multiple causes) Premature Mortality in Women (1-74 yr), Belgium 2003-2009

# Ranking of the arrondissements by increased mortality

# Age-adjusted rates per 100.000

| Rank | ARROND | Age-adj.Rates | CI on age-adj.Rates | smr | p value\* |
| --- | --- | --- | --- | --- | --- |
| 1 | Veurne | 7.6 | [ 4.2;11.0] | 51.3 | <0.001 |
| 2 | Oudenaarde | 9.2 | [ 6.2;12.1] | 63.3 | <0.001 |
| 3 | Ieper | 10.1 | [ 6.8;13.3] | 69.9 | <0.01 |
| 4 | Sint Niklaas | 10.1 | [ 7.9;12.3] | 69.0 | <0.001 |
| 5 | Antwerpen | 10.2 | [ 9.1;11.3] | 69.6 | <0.001 |
| 6 | Hasselt | 10.3 | [ 8.6;12.0] | 69.5 | <0.001 |
| 7 | Halle-Vilvoorde | 10.3 | [ 8.9;11.7] | 70.4 | <0.001 |
| 8 | Brugge | 10.3 | [ 8.4;12.3] | 69.5 | <0.001 |
| 9 | Leuven | 10.6 | [ 9.0;12.3] | 72.1 | <0.001 |
| 10 | Gent | 10.7 | [ 9.1;12.2] | 72.9 | <0.001 |
| 11 | Turnhout | 10.7 | [ 9.0;12.5] | 72.7 | <0.001 |
| 12 | Kortrijk | 10.8 | [ 8.7;12.9] | 73.2 | <0.001 |
| 13 | Oostende | 10.8 | [ 8.2;13.5] | 72.4 | <0.01 |
| 14 | Mechelen | 10.9 | [ 8.9;12.8] | 74.4 | <0.001 |
| 15 | Eeklo | 11.2 | [ 7.4;15.1] | 76.6 | ns. |
| 16 | Tongeren | 11.8 | [ 9.2;14.5] | 80.5 | <0.05 |
| 17 | Dendermonde | 12.3 | [ 9.6;15.0] | 84.4 | ns. |
| 18 | Neufchateau | 12.5 | [ 7.3;17.8] | 84.2 | ns. |
| 19 | Roeselare | 12.7 | [ 9.6;15.8] | 85.6 | ns. |
| 20 | Arlon | 12.8 | [ 7.3;18.2] | 87.6 | ns. |
| 21 | Nivelles | 12.8 | [10.8;14.9] | 87.4 | ns. |
| 22 | Tielt | 12.9 | [ 8.8;16.9] | 88.3 | ns. |
| 23 | Maaseik | 13.0 | [10.3;15.6] | 87.6 | ns. |
| 24 | Aalst | 14.7 | [12.3;17.1] | 101.0 | ns. |
| 25 | Verviers | 18.1 | [15.2;20.9] | 122.7 | <0.05 |
| 26 | Li�ge | 18.1 | [16.2;19.9] | 121.4 | <0.001 |
| 27 | Thuin | 19.1 | [15.3;23.0] | 129.8 | <0.05 |
| 28 | Huy | 19.3 | [14.5;24.2] | 130.8 | ns. |
| 29 | Brussels | 19.6 | [18.0;21.2] | 133.4 | <0.001 |
| 30 | Virton | 19.8 | [12.7;27.0] | 133.5 | ns. |
| 31 | Marche-en-Famenne | 19.9 | [13.1;26.7] | 136.7 | ns. |
| 32 | Waremme | 20.0 | [14.3;25.8] | 137.7 | ns. |
| 33 | Dinant | 20.2 | [15.4;24.9] | 135.9 | <0.05 |
| 34 | Namur | 20.9 | [17.9;23.8] | 143.2 | <0.001 |
| 35 | Philippeville | 21.6 | [15.3;27.9] | 148.3 | <0.05 |
| 36 | Bastogne | 22.0 | [13.8;30.2] | 150.4 | ns. |
| 37 | Diksmuide | 22.1 | [15.0;29.3] | 153.7 | <0.05 |
| 38 | Tournai | 22.7 | [18.3;27.1] | 152.6 | <0.001 |
| 39 | Ath | 23.6 | [17.8;29.5] | 162.4 | <0.01 |
| 40 | Mons | 23.8 | [20.4;27.1] | 163.1 | <0.001 |
| 41 | Soignies | 24.2 | [20.1;28.2] | 165.5 | <0.001 |
| 42 | Mouscron | 26.1 | [19.6;32.6] | 179.2 | <0.001 |
| 43 | Charleroi | 28.3 | [25.5;31.1] | 192.7 | <0.001 |

  

# Mean Rate = 14.7

# 

# \* p value of the z statistic testing for a the difference between the arrondissement's rate and the mean rate
