# Supplementary material for: Mapping the cause-specific premature mortality reveals large between-districts disparity in Belgium, 2003–2009
Source: Arch Public Health. 2015 Mar 23;73(1):13. doi: 10.1186/s13690-015-0060-5 (PMC4412101; doi:10.1186/s13690-015-0060-5)
Supplement: Additional file 41: Table S16. — Mental & neurol. Dis (excluding alc-rel) Men 175. [file 13690_2015_60_MOESM41_ESM.zip › 13690_2015_60_MOESM41_ESM.html]

SAS Output


# Mental&neurol.Dis (excluding alc-rel) Premature Mortality in Men (1-74 yr), Belgium 2003-2009

# Ranking of the arrondissements by increased mortality

# Age-adjusted rates per 100.000

| Rank | ARROND | Age-adj.Rates | CI on age-adj.Rates | smr | p value\* |
| --- | --- | --- | --- | --- | --- |
| 1 | Oudenaarde | 9.6 | [ 6.4;12.8] | 55.2 | <0.001 |
| 2 | Diksmuide | 9.8 | [ 4.8;14.7] | 56.3 | <0.01 |
| 3 | Hasselt | 10.7 | [ 8.9;12.5] | 62.3 | <0.001 |
| 4 | Roeselare | 11.6 | [ 8.5;14.7] | 68.4 | <0.001 |
| 5 | Sint Niklaas | 11.8 | [ 9.3;14.3] | 68.7 | <0.001 |
| 6 | Ieper | 11.8 | [ 8.1;15.5] | 70.7 | <0.01 |
| 7 | Eeklo | 12.5 | [ 8.3;16.6] | 74.5 | <0.05 |
| 8 | Turnhout | 12.9 | [11.0;14.9] | 74.0 | <0.001 |
| 9 | Halle-Vilvoorde | 13.3 | [11.6;15.0] | 77.0 | <0.001 |
| 10 | Leuven | 13.4 | [11.5;15.3] | 78.0 | <0.001 |
| 11 | Kortrijk | 13.6 | [11.2;16.0] | 80.3 | <0.01 |
| 12 | Tielt | 13.8 | [ 9.5;18.1] | 79.7 | ns. |
| 13 | Gent | 14.2 | [12.3;16.1] | 82.3 | <0.01 |
| 14 | Tongeren | 14.2 | [11.2;17.2] | 82.5 | ns. |
| 15 | Maaseik | 14.5 | [11.7;17.3] | 83.9 | ns. |
| 16 | Mechelen | 14.9 | [12.5;17.3] | 86.4 | ns. |
| 17 | Veurne | 15.3 | [10.2;20.5] | 89.2 | ns. |
| 18 | Dendermonde | 15.3 | [12.2;18.5] | 89.5 | ns. |
| 19 | Brugge | 15.7 | [13.1;18.2] | 90.8 | ns. |
| 20 | Oostende | 16.0 | [12.5;19.4] | 89.8 | ns. |
| 21 | Antwerpen | 16.0 | [14.5;17.4] | 92.4 | ns. |
| 22 | Nivelles | 16.9 | [14.4;19.4] | 98.0 | ns. |
| 23 | Mouscron | 17.5 | [11.9;23.1] | 106.7 | ns. |
| 24 | Aalst | 17.7 | [14.9;20.5] | 102.4 | ns. |
| 25 | Brussels | 17.9 | [16.2;19.6] | 103.5 | ns. |
| 26 | Ath | 18.3 | [12.8;23.8] | 108.9 | ns. |
| 27 | Marche-en-Famenne | 18.5 | [11.7;25.3] | 110.6 | ns. |
| 28 | Arlon | 19.7 | [12.5;26.9] | 110.6 | ns. |
| 29 | Waremme | 19.7 | [13.6;25.8] | 114.2 | ns. |
| 30 | Philippeville | 19.7 | [13.5;26.0] | 118.2 | ns. |
| 31 | Neufchateau | 21.8 | [14.9;28.7] | 135.9 | ns. |
| 32 | Thuin | 22.6 | [18.1;27.1] | 133.1 | <0.05 |
| 33 | Li�ge | 23.5 | [21.3;25.8] | 136.7 | <0.001 |
| 34 | Verviers | 23.9 | [20.5;27.3] | 136.7 | <0.001 |
| 35 | Bastogne | 24.0 | [15.0;32.9] | 138.2 | ns. |
| 36 | Soignies | 24.1 | [19.8;28.5] | 140.9 | <0.01 |
| 37 | Huy | 24.6 | [18.9;30.3] | 145.1 | <0.05 |
| 38 | Charleroi | 25.0 | [22.1;27.8] | 147.1 | <0.001 |
| 39 | Namur | 25.4 | [21.9;28.8] | 150.9 | <0.001 |
| 40 | Virton | 25.7 | [17.3;34.1] | 151.6 | <0.05 |
| 41 | Mons | 27.1 | [23.2;31.0] | 159.6 | <0.001 |
| 42 | Dinant | 27.8 | [21.9;33.6] | 165.5 | <0.001 |
| 43 | Tournai | 28.3 | [23.1;33.6] | 162.5 | <0.001 |

  

# Mean Rate = 17.2

# 

# \* p value of the z statistic testing for a the difference between the arrondissement's rate and the mean rate
