# Supplementary material for: Mapping the cause-specific premature mortality reveals large between-districts disparity in Belgium, 2003–2009
Source: Arch Public Health. 2015 Mar 23;73(1):13. doi: 10.1186/s13690-015-0060-5 (PMC4412101; doi:10.1186/s13690-015-0060-5)
Supplement: Additional file 42: Table S17. — Mental & neurol. Dis (excluding alc-rel) Women 175. [file 13690_2015_60_MOESM42_ESM.zip › 13690_2015_60_MOESM42_ESM.html]

SAS Output


# Mental&neurol.Dis (excluding alc-rel) Premature Mortality in Women (1-74 yr), Belgium 2003-2009

# Ranking of the arrondissements by increased mortality

# Age-adjusted rates per 100.000

| Rank | ARROND | Age-adj.Rates | CI on age-adj.Rates | smr | p value\* |
| --- | --- | --- | --- | --- | --- |
| 1 | Ieper | 4.3 | [ 2.2; 6.5] | 36.8 | <0.001 |
| 2 | Diksmuide | 7.7 | [ 3.3;12.0] | 60.6 | <0.05 |
| 3 | Oostende | 9.0 | [ 6.5;11.5] | 70.4 | <0.05 |
| 4 | Eeklo | 9.3 | [ 5.7;12.9] | 73.9 | ns. |
| 5 | Dendermonde | 9.4 | [ 7.1;11.8] | 77.3 | <0.05 |
| 6 | Kortrijk | 9.5 | [ 7.6;11.4] | 78.4 | <0.01 |
| 7 | Halle-Vilvoorde | 9.7 | [ 8.3;11.0] | 78.8 | <0.001 |
| 8 | Gent | 9.9 | [ 8.4;11.4] | 80.6 | <0.01 |
| 9 | Brugge | 9.9 | [ 8.0;11.9] | 82.5 | <0.05 |
| 10 | Sint Niklaas | 10.0 | [ 7.7;12.2] | 82.4 | <0.05 |
| 11 | Roeselare | 10.3 | [ 7.5;13.1] | 85.1 | ns. |
| 12 | Hasselt | 10.5 | [ 8.7;12.2] | 84.1 | ns. |
| 13 | Turnhout | 10.5 | [ 8.8;12.2] | 87.1 | <0.05 |
| 14 | Tielt | 10.6 | [ 6.8;14.3] | 85.2 | ns. |
| 15 | Maaseik | 10.7 | [ 8.3;13.1] | 86.5 | ns. |
| 16 | Oudenaarde | 10.7 | [ 7.6;13.9] | 89.2 | ns. |
| 17 | Leuven | 11.2 | [ 9.5;12.8] | 90.4 | ns. |
| 18 | Marche-en-Famenne | 11.3 | [ 6.2;16.4] | 94.0 | ns. |
| 19 | Antwerpen | 11.3 | [10.2;12.5] | 92.5 | ns. |
| 20 | Mechelen | 11.3 | [ 9.3;13.3] | 92.4 | ns. |
| 21 | Veurne | 11.6 | [ 7.0;16.2] | 87.6 | ns. |
| 22 | Tongeren | 11.8 | [ 9.1;14.4] | 95.6 | ns. |
| 23 | Dinant | 12.1 | [ 8.4;15.8] | 97.6 | ns. |
| 24 | Aalst | 12.1 | [ 9.9;14.3] | 98.3 | ns. |
| 25 | Brussels | 12.3 | [11.0;13.5] | 100.3 | ns. |
| 26 | Nivelles | 12.6 | [10.5;14.6] | 101.9 | ns. |
| 27 | Soignies | 12.6 | [ 9.7;15.5] | 100.8 | ns. |
| 28 | Philippeville | 13.5 | [ 8.5;18.5] | 108.7 | ns. |
| 29 | Li�ge | 15.6 | [13.9;17.3] | 128.0 | <0.001 |
| 30 | Namur | 15.7 | [13.1;18.3] | 126.1 | <0.01 |
| 31 | Neufchateau | 15.9 | [10.1;21.7] | 132.5 | ns. |
| 32 | Waremme | 16.2 | [11.0;21.4] | 132.8 | ns. |
| 33 | Thuin | 16.3 | [12.8;19.8] | 136.2 | <0.05 |
| 34 | Mons | 16.4 | [13.6;19.1] | 133.8 | <0.01 |
| 35 | Verviers | 16.4 | [13.7;19.1] | 134.4 | <0.01 |
| 36 | Bastogne | 16.4 | [ 9.4;23.5] | 133.7 | ns. |
| 37 | Ath | 16.8 | [11.9;21.8] | 137.1 | ns. |
| 38 | Mouscron | 17.3 | [12.0;22.6] | 140.9 | ns. |
| 39 | Charleroi | 17.5 | [15.3;19.8] | 143.7 | <0.001 |
| 40 | Virton | 17.6 | [10.9;24.2] | 142.9 | ns. |
| 41 | Tournai | 18.0 | [14.1;21.9] | 145.0 | <0.01 |
| 42 | Huy | 19.3 | [14.5;24.0] | 160.3 | <0.01 |
| 43 | Arlon | 19.9 | [13.1;26.6] | 162.6 | <0.05 |

  

# Mean Rate = 12.2

# 

# \* p value of the z statistic testing for a the difference between the arrondissement's rate and the mean rate
