# Supplementary material for: Mapping the cause-specific premature mortality reveals large between-districts disparity in Belgium, 2003–2009
Source: Arch Public Health. 2015 Mar 23;73(1):13. doi: 10.1186/s13690-015-0060-5 (PMC4412101; doi:10.1186/s13690-015-0060-5)
Supplement: Additional file 43: Table S18. — C.O.P.D. Men 175. [file 13690_2015_60_MOESM43_ESM.zip › 13690_2015_60_MOESM43_ESM.html]

SAS Output


# C.O.P.D. Premature Mortality in Men (1-74 yr), Belgium 2003-2009

# Ranking of the arrondissements by increased mortality

# Age-adjusted rates per 100.000

| Rank | ARROND | Age-adj.Rates | CI on age-adj.Rates | smr | p value\* |
| --- | --- | --- | --- | --- | --- |
| 1 | Brugge | 14.1 | [11.7;16.5] | 62.5 | <0.001 |
| 2 | Diksmuide | 14.8 | [ 8.7;20.8] | 65.9 | <0.05 |
| 3 | Oostende | 14.8 | [11.6;18.0] | 66.1 | <0.001 |
| 4 | Maaseik | 15.7 | [12.7;18.6] | 67.9 | <0.001 |
| 5 | Kortrijk | 15.9 | [13.2;18.5] | 70.2 | <0.001 |
| 6 | Oudenaarde | 15.9 | [11.8;20.0] | 70.3 | <0.01 |
| 7 | Veurne | 16.6 | [11.5;21.8] | 74.1 | <0.05 |
| 8 | Halle-Vilvoorde | 16.8 | [14.9;18.7] | 74.2 | <0.001 |
| 9 | Antwerpen | 16.8 | [15.3;18.3] | 74.0 | <0.001 |
| 10 | Roeselare | 17.4 | [13.6;21.1] | 77.1 | <0.01 |
| 11 | Sint Niklaas | 17.4 | [14.3;20.5] | 76.1 | <0.01 |
| 12 | Leuven | 18.7 | [16.4;20.9] | 81.8 | <0.001 |
| 13 | Mechelen | 20.1 | [17.3;22.9] | 87.5 | ns. |
| 14 | Dendermonde | 20.3 | [16.6;23.9] | 88.9 | ns. |
| 15 | Tielt | 20.4 | [15.1;25.7] | 89.2 | ns. |
| 16 | Eeklo | 20.4 | [15.0;25.7] | 89.5 | ns. |
| 17 | Tongeren | 20.6 | [17.0;24.3] | 89.7 | ns. |
| 18 | Arlon | 20.8 | [13.2;28.4] | 90.0 | ns. |
| 19 | Gent | 21.0 | [18.7;23.3] | 92.9 | ns. |
| 20 | Nivelles | 21.1 | [18.2;24.0] | 91.7 | ns. |
| 21 | Hasselt | 21.3 | [18.7;23.9] | 93.1 | ns. |
| 22 | Ieper | 22.1 | [17.0;27.2] | 98.8 | ns. |
| 23 | Neufchateau | 22.4 | [15.1;29.6] | 101.8 | ns. |
| 24 | Aalst | 23.6 | [20.4;26.9] | 104.1 | ns. |
| 25 | Turnhout | 23.8 | [21.1;26.4] | 103.8 | ns. |
| 26 | Verviers | 24.0 | [20.6;27.5] | 106.7 | ns. |
| 27 | Ath | 24.6 | [18.1;31.2] | 108.3 | ns. |
| 28 | Tournai | 25.2 | [20.3;30.2] | 115.8 | ns. |
| 29 | Marche-en-Famenne | 25.6 | [17.4;33.8] | 115.2 | ns. |
| 30 | Brussels | 25.6 | [23.5;27.7] | 112.9 | <0.01 |
| 31 | Mouscron | 25.8 | [18.8;32.8] | 116.1 | ns. |
| 32 | Soignies | 27.6 | [22.9;32.4] | 121.8 | <0.05 |
| 33 | Huy | 28.0 | [21.8;34.2] | 126.0 | ns. |
| 34 | Dinant | 29.4 | [23.2;35.5] | 130.4 | <0.05 |
| 35 | Bastogne | 29.7 | [19.5;39.9] | 132.5 | ns. |
| 36 | Namur | 30.1 | [26.2;34.0] | 135.3 | <0.001 |
| 37 | Virton | 30.5 | [21.1;40.0] | 134.2 | ns. |
| 38 | Waremme | 30.6 | [22.7;38.4] | 131.4 | <0.05 |
| 39 | Li�ge | 32.3 | [29.6;35.0] | 143.7 | <0.001 |
| 40 | Thuin | 33.6 | [28.0;39.2] | 150.2 | <0.001 |
| 41 | Mons | 36.8 | [32.0;41.5] | 163.8 | <0.001 |
| 42 | Philippeville | 38.9 | [30.0;47.9] | 175.3 | <0.001 |
| 43 | Charleroi | 39.5 | [35.8;43.2] | 176.9 | <0.001 |

  

# Mean Rate = 22.6

# 

# \* p value of the z statistic testing for a the difference between the arrondissement's rate and the mean rate
