# Supplementary material for: Mapping the cause-specific premature mortality reveals large between-districts disparity in Belgium, 2003–2009
Source: Arch Public Health. 2015 Mar 23;73(1):13. doi: 10.1186/s13690-015-0060-5 (PMC4412101; doi:10.1186/s13690-015-0060-5)
Supplement: Additional file 44: Table S19. — C.O.P.D. Women 175. [file 13690_2015_60_MOESM44_ESM.zip › 13690_2015_60_MOESM44_ESM.html]

SAS Output


# C.O.P.D. Premature Mortality in Women (1-74 yr), Belgium 2003-2009

# Ranking of the arrondissements by increased mortality

# Age-adjusted rates per 100.000

| Rank | ARROND | Age-adj.Rates | CI on age-adj.Rates | smr | p value\* |
| --- | --- | --- | --- | --- | --- |
| 1 | Diksmuide | 2.5 | [ 0.0; 5.0] | 29.3 | <0.001 |
| 2 | Mouscron | 2.9 | [ 0.7; 5.0] | 34.8 | <0.001 |
| 3 | Tielt | 3.0 | [ 1.0; 4.9] | 35.8 | <0.001 |
| 4 | Oudenaarde | 3.2 | [ 1.5; 5.0] | 38.2 | <0.001 |
| 5 | Roeselare | 3.2 | [ 1.7; 4.8] | 37.6 | <0.001 |
| 6 | Virton | 3.9 | [ 0.8; 6.9] | 47.2 | <0.01 |
| 7 | Eeklo | 4.4 | [ 2.0; 6.8] | 53.0 | <0.01 |
| 8 | Ieper | 4.4 | [ 2.3; 6.6] | 53.1 | <0.001 |
| 9 | Veurne | 4.5 | [ 1.8; 7.1] | 49.2 | <0.01 |
| 10 | Oostende | 4.9 | [ 3.2; 6.7] | 58.8 | <0.001 |
| 11 | Kortrijk | 5.5 | [ 4.0; 7.0] | 65.4 | <0.001 |
| 12 | Halle-Vilvoorde | 5.7 | [ 4.6; 6.7] | 66.9 | <0.001 |
| 13 | Leuven | 5.8 | [ 4.6; 7.0] | 67.9 | <0.001 |
| 14 | Gent | 5.9 | [ 4.7; 7.0] | 70.1 | <0.001 |
| 15 | Aalst | 5.9 | [ 4.4; 7.4] | 71.6 | <0.01 |
| 16 | Ath | 5.9 | [ 3.0; 8.8] | 71.0 | ns. |
| 17 | Marche-en-Famenne | 6.0 | [ 2.3; 9.7] | 72.5 | ns. |
| 18 | Brugge | 6.1 | [ 4.6; 7.6] | 73.1 | <0.01 |
| 19 | Mechelen | 6.3 | [ 4.8; 7.8] | 75.7 | <0.01 |
| 20 | Nivelles | 6.5 | [ 5.0; 8.0] | 76.1 | <0.05 |
| 21 | Tournai | 6.7 | [ 4.3; 9.1] | 78.8 | ns. |
| 22 | Sint Niklaas | 6.9 | [ 5.1; 8.8] | 82.2 | ns. |
| 23 | Maaseik | 7.3 | [ 5.3; 9.3] | 85.1 | ns. |
| 24 | Turnhout | 7.4 | [ 6.0; 8.8] | 88.6 | ns. |
| 25 | Dendermonde | 7.5 | [ 5.4; 9.6] | 90.1 | ns. |
| 26 | Hasselt | 8.0 | [ 6.5; 9.5] | 97.0 | ns. |
| 27 | Tongeren | 8.1 | [ 5.9;10.3] | 96.3 | ns. |
| 28 | Antwerpen | 8.1 | [ 7.1; 9.1] | 96.8 | ns. |
| 29 | Soignies | 8.1 | [ 5.8;10.5] | 97.5 | ns. |
| 30 | Philippeville | 8.3 | [ 4.3;12.2] | 95.4 | ns. |
| 31 | Brussels | 9.7 | [ 8.5;10.8] | 114.4 | <0.05 |
| 32 | Mons | 9.9 | [ 7.7;12.1] | 116.6 | ns. |
| 33 | Bastogne | 10.0 | [ 4.6;15.5] | 123.0 | ns. |
| 34 | Thuin | 10.5 | [ 7.6;13.4] | 125.1 | ns. |
| 35 | Dinant | 10.8 | [ 7.3;14.3] | 127.6 | ns. |
| 36 | Arlon | 11.3 | [ 6.1;16.6] | 131.7 | ns. |
| 37 | Namur | 12.5 | [10.2;14.8] | 152.7 | <0.001 |
| 38 | Charleroi | 13.1 | [11.2;15.0] | 154.6 | <0.001 |
| 39 | Verviers | 13.2 | [10.8;15.6] | 159.1 | <0.001 |
| 40 | Neufchateau | 13.7 | [ 8.1;19.2] | 154.5 | ns. |
| 41 | Huy | 14.9 | [10.6;19.1] | 176.5 | <0.01 |
| 42 | Waremme | 17.6 | [12.2;23.0] | 210.0 | <0.001 |
| 43 | Li�ge | 18.9 | [17.0;20.8] | 225.8 | <0.001 |

  

# Mean Rate = 8.4

# 

# \* p value of the z statistic testing for a the difference between the arrondissement's rate and the mean rate
