# Supplementary material for: Mapping the cause-specific premature mortality reveals large between-districts disparity in Belgium, 2003–2009
Source: Arch Public Health. 2015 Mar 23;73(1):13. doi: 10.1186/s13690-015-0060-5 (PMC4412101; doi:10.1186/s13690-015-0060-5)
Supplement: Additional file 45: Table S20. — Alcohol-related (analysis in multiple causes) Men 175. [file 13690_2015_60_MOESM45_ESM.zip › 13690_2015_60_MOESM45_ESM.html]

SAS Output


# Alcohol-related (analysis in multiple causes) Premature Mortality in Men (1-74 yr), Belgium 2003-2009

# Ranking of the arrondissements by increased mortality

# Age-adjusted rates per 100.000

| Rank | ARROND | Age-adj.Rates | CI on age-adj.Rates | smr | p value\* |
| --- | --- | --- | --- | --- | --- |
| 1 | Maaseik | 8.7 | [ 6.6;10.8] | 22.8 | <0.001 |
| 2 | Turnhout | 12.8 | [11.0;14.7] | 34.3 | <0.001 |
| 3 | Hasselt | 14.8 | [12.8;16.9] | 40.3 | <0.001 |
| 4 | Tongeren | 16.0 | [12.9;19.0] | 42.0 | <0.001 |
| 5 | Mechelen | 19.7 | [17.0;22.4] | 53.6 | <0.001 |
| 6 | Tielt | 21.2 | [15.9;26.5] | 57.2 | <0.001 |
| 7 | Leuven | 21.4 | [19.1;23.7] | 58.0 | <0.001 |
| 8 | Roeselare | 21.5 | [17.3;25.6] | 57.9 | <0.001 |
| 9 | Eeklo | 22.1 | [16.6;27.5] | 61.6 | <0.001 |
| 10 | Antwerpen | 22.1 | [20.4;23.7] | 60.1 | <0.001 |
| 11 | Diksmuide | 22.9 | [15.5;30.3] | 62.7 | <0.001 |
| 12 | Sint Niklaas | 23.3 | [19.8;26.7] | 63.3 | <0.001 |
| 13 | Gent | 26.5 | [24.0;29.0] | 71.9 | <0.001 |
| 14 | Brugge | 27.2 | [23.9;30.5] | 74.5 | <0.001 |
| 15 | Dendermonde | 29.5 | [25.3;33.8] | 78.7 | <0.01 |
| 16 | Halle-Vilvoorde | 31.4 | [28.9;34.0] | 84.8 | <0.001 |
| 17 | Ieper | 32.0 | [26.0;38.0] | 86.8 | ns. |
| 18 | Kortrijk | 32.2 | [28.5;35.9] | 87.7 | <0.05 |
| 19 | Oostende | 33.5 | [28.6;38.4] | 90.4 | ns. |
| 20 | Waremme | 34.1 | [26.4;41.7] | 93.8 | ns. |
| 21 | Oudenaarde | 37.5 | [31.3;43.7] | 101.8 | ns. |
| 22 | Verviers | 39.7 | [35.5;43.9] | 108.2 | ns. |
| 23 | Aalst | 39.8 | [35.7;43.9] | 108.9 | ns. |
| 24 | Veurne | 41.5 | [33.0;50.1] | 110.5 | ns. |
| 25 | Nivelles | 41.8 | [38.0;45.6] | 112.9 | <0.05 |
| 26 | Bastogne | 44.2 | [32.4;56.1] | 119.9 | ns. |
| 27 | Huy | 46.8 | [39.2;54.4] | 126.6 | <0.01 |
| 28 | Li�ge | 50.5 | [47.3;53.7] | 138.1 | <0.001 |
| 29 | Dinant | 51.6 | [43.8;59.4] | 142.3 | <0.001 |
| 30 | Neufchateau | 52.2 | [41.5;62.9] | 142.9 | <0.01 |
| 31 | Marche-en-Famenne | 55.0 | [43.5;66.5] | 148.6 | <0.01 |
| 32 | Brussels | 55.8 | [52.9;58.7] | 149.7 | <0.001 |
| 33 | Arlon | 58.0 | [46.2;69.8] | 160.5 | <0.001 |
| 34 | Namur | 59.8 | [54.7;64.9] | 163.0 | <0.001 |
| 35 | Virton | 60.3 | [47.8;72.8] | 167.3 | <0.001 |
| 36 | Ath | 60.9 | [51.2;70.6] | 164.6 | <0.001 |
| 37 | Soignies | 63.3 | [56.6;70.1] | 173.8 | <0.001 |
| 38 | Philippeville | 63.9 | [53.1;74.7] | 179.8 | <0.001 |
| 39 | Tournai | 67.4 | [59.7;75.1] | 184.9 | <0.001 |
| 40 | Thuin | 67.7 | [60.2;75.2] | 183.4 | <0.001 |
| 41 | Mouscron | 68.3 | [57.2;79.3] | 182.9 | <0.001 |
| 42 | Charleroi | 70.2 | [65.6;74.8] | 190.9 | <0.001 |
| 43 | Mons | 78.6 | [72.2;85.0] | 216.1 | <0.001 |

  

# Mean Rate = 36.7

# 

# \* p value of the z statistic testing for a the difference between the arrondissement's rate and the mean rate
