# Supplementary material for: Mapping the cause-specific premature mortality reveals large between-districts disparity in Belgium, 2003–2009
Source: Arch Public Health. 2015 Mar 23;73(1):13. doi: 10.1186/s13690-015-0060-5 (PMC4412101; doi:10.1186/s13690-015-0060-5)
Supplement: Additional file 46: Table S21. — Alcohol-related (analysis in multiple causes) Women 175. [file 13690_2015_60_MOESM46_ESM.zip › 13690_2015_60_MOESM46_ESM.html]

SAS Output


# Alcohol-related (analysis in multiple causes) Premature Mortality in Women (1-74 yr), Belgium 2003-2009

# Ranking of the arrondissements by increased mortality

# Age-adjusted rates per 100.000

| Rank | ARROND | Age-adj.Rates | CI on age-adj.Rates | smr | p value\* |
| --- | --- | --- | --- | --- | --- |
| 1 | Maaseik | 3.5 | [ 2.2; 4.9] | 25.8 | <0.001 |
| 2 | Turnhout | 4.4 | [ 3.3; 5.5] | 31.6 | <0.001 |
| 3 | Hasselt | 4.7 | [ 3.6; 5.9] | 34.9 | <0.001 |
| 4 | Tongeren | 4.8 | [ 3.2; 6.5] | 35.7 | <0.001 |
| 5 | Mechelen | 5.6 | [ 4.2; 7.0] | 41.3 | <0.001 |
| 6 | Leuven | 6.9 | [ 5.6; 8.2] | 51.1 | <0.001 |
| 7 | Diksmuide | 6.9 | [ 2.8;11.1] | 51.0 | <0.01 |
| 8 | Tielt | 7.0 | [ 3.9;10.1] | 50.3 | <0.001 |
| 9 | Sint Niklaas | 8.3 | [ 6.3;10.3] | 61.5 | <0.001 |
| 10 | Antwerpen | 8.7 | [ 7.6; 9.7] | 63.2 | <0.001 |
| 11 | Brugge | 9.2 | [ 7.3;11.1] | 67.4 | <0.001 |
| 12 | Ieper | 9.8 | [ 6.5;13.1] | 71.6 | <0.05 |
| 13 | Roeselare | 10.2 | [ 7.4;13.1] | 73.9 | <0.05 |
| 14 | Dendermonde | 10.9 | [ 8.4;13.5] | 80.0 | <0.05 |
| 15 | Kortrijk | 11.2 | [ 9.1;13.3] | 82.6 | <0.05 |
| 16 | Halle-Vilvoorde | 11.6 | [10.2;13.1] | 85.6 | <0.05 |
| 17 | Gent | 11.9 | [10.3;13.6] | 87.7 | <0.05 |
| 18 | Verviers | 12.1 | [ 9.8;14.3] | 88.9 | ns. |
| 19 | Eeklo | 12.2 | [ 8.1;16.3] | 88.3 | ns. |
| 20 | Waremme | 12.5 | [ 8.0;17.1] | 90.9 | ns. |
| 21 | Aalst | 13.6 | [11.3;16.0] | 100.1 | ns. |
| 22 | Oudenaarde | 13.7 | [10.0;17.3] | 100.5 | ns. |
| 23 | Veurne | 13.9 | [ 9.1;18.6] | 102.3 | ns. |
| 24 | Nivelles | 14.6 | [12.4;16.7] | 104.9 | ns. |
| 25 | Oostende | 15.6 | [12.3;18.8] | 115.2 | ns. |
| 26 | Bastogne | 16.6 | [ 9.5;23.7] | 120.2 | ns. |
| 27 | Neufchateau | 16.6 | [10.7;22.6] | 122.5 | ns. |
| 28 | Li�ge | 17.7 | [15.8;19.5] | 129.6 | <0.001 |
| 29 | Huy | 18.6 | [14.0;23.2] | 134.9 | <0.05 |
| 30 | Virton | 19.9 | [12.8;27.1] | 143.4 | ns. |
| 31 | Brussels | 20.0 | [18.4;21.6] | 145.5 | <0.001 |
| 32 | Arlon | 21.4 | [14.4;28.4] | 156.7 | <0.05 |
| 33 | Namur | 21.5 | [18.5;24.5] | 157.2 | <0.001 |
| 34 | Thuin | 23.1 | [18.9;27.3] | 170.2 | <0.001 |
| 35 | Soignies | 23.1 | [19.2;27.0] | 166.6 | <0.001 |
| 36 | Marche-en-Famenne | 23.9 | [16.5;31.4] | 174.3 | <0.01 |
| 37 | Charleroi | 24.7 | [22.1;27.3] | 181.3 | <0.001 |
| 38 | Philippeville | 25.2 | [18.5;31.9] | 186.3 | <0.001 |
| 39 | Tournai | 25.3 | [20.7;29.9] | 181.8 | <0.001 |
| 40 | Dinant | 26.7 | [21.2;32.2] | 195.6 | <0.001 |
| 41 | Mouscron | 27.3 | [20.7;33.9] | 202.2 | <0.001 |
| 42 | Ath | 27.6 | [21.2;33.9] | 195.9 | <0.001 |
| 43 | Mons | 30.6 | [26.9;34.4] | 221.5 | <0.001 |

  

# Mean Rate = 13.7

# 

# \* p value of the z statistic testing for a the difference between the arrondissement's rate and the mean rate
