# Supplementary material for: Mapping the cause-specific premature mortality reveals large between-districts disparity in Belgium, 2003–2009
Source: Arch Public Health. 2015 Mar 23;73(1):13. doi: 10.1186/s13690-015-0060-5 (PMC4412101; doi:10.1186/s13690-015-0060-5)
Supplement: Additional file 47: Table S22. — Road Accident Men 175. [file 13690_2015_60_MOESM47_ESM.zip › 13690_2015_60_MOESM47_ESM.html]

SAS Output


# Road Accident Premature Mortality in Men (1-74 yr), Belgium 2003-2009

# Ranking of the arrondissements by increased mortality

# Age-adjusted rates per 100.000

| Rank | ARROND | Age-adj.Rates | CI on age-adj.Rates | smr | p value\* |
| --- | --- | --- | --- | --- | --- |
| 1 | Brussels | 6.6 | [ 5.7; 7.5] | 39.5 | <0.001 |
| 2 | Antwerpen | 10.8 | [ 9.6;12.0] | 66.6 | <0.001 |
| 3 | Leuven | 10.8 | [ 9.1;12.5] | 67.0 | <0.001 |
| 4 | Halle-Vilvoorde | 12.4 | [10.7;14.0] | 75.8 | <0.001 |
| 5 | Mechelen | 12.5 | [10.3;14.7] | 76.6 | <0.01 |
| 6 | Gent | 12.5 | [10.8;14.2] | 77.6 | <0.001 |
| 7 | Kortrijk | 13.1 | [10.7;15.5] | 81.4 | <0.05 |
| 8 | Sint Niklaas | 13.4 | [10.7;16.1] | 81.4 | <0.05 |
| 9 | Dendermonde | 14.5 | [11.5;17.6] | 90.8 | ns. |
| 10 | Aalst | 14.8 | [12.2;17.4] | 93.3 | ns. |
| 11 | Hasselt | 15.2 | [13.1;17.4] | 93.4 | ns. |
| 12 | Turnhout | 15.6 | [13.5;17.7] | 95.9 | ns. |
| 13 | Brugge | 16.2 | [13.5;19.0] | 99.1 | ns. |
| 14 | Li�ge | 16.4 | [14.6;18.3] | 101.9 | ns. |
| 15 | Nivelles | 16.9 | [14.5;19.3] | 105.4 | ns. |
| 16 | Roeselare | 17.4 | [13.6;21.3] | 107.7 | ns. |
| 17 | Oostende | 17.7 | [13.8;21.6] | 109.8 | ns. |
| 18 | Maaseik | 18.3 | [15.2;21.4] | 112.2 | ns. |
| 19 | Tongeren | 18.3 | [15.0;21.7] | 114.7 | ns. |
| 20 | Eeklo | 18.7 | [13.3;24.1] | 112.0 | ns. |
| 21 | Verviers | 18.9 | [16.0;21.8] | 118.9 | ns. |
| 22 | Oudenaarde | 19.0 | [14.5;23.6] | 117.4 | ns. |
| 23 | Soignies | 20.0 | [16.3;23.8] | 124.5 | <0.05 |
| 24 | Tielt | 20.2 | [15.0;25.5] | 124.0 | ns. |
| 25 | Veurne | 20.5 | [13.7;27.4] | 126.3 | ns. |
| 26 | Tournai | 20.7 | [16.5;24.9] | 129.7 | <0.05 |
| 27 | Mouscron | 21.6 | [15.4;27.8] | 134.5 | ns. |
| 28 | Ieper | 22.5 | [17.4;27.6] | 142.2 | <0.05 |
| 29 | Charleroi | 22.6 | [20.0;25.2] | 141.0 | <0.001 |
| 30 | Namur | 23.2 | [20.1;26.3] | 144.5 | <0.001 |
| 31 | Mons | 23.4 | [20.0;26.9] | 143.8 | <0.001 |
| 32 | Arlon | 23.6 | [16.4;30.8] | 145.4 | <0.05 |
| 33 | Huy | 25.2 | [19.7;30.8] | 153.6 | <0.01 |
| 34 | Waremme | 26.9 | [20.1;33.7] | 168.8 | <0.01 |
| 35 | Bastogne | 27.1 | [18.4;35.7] | 169.3 | <0.05 |
| 36 | Diksmuide | 27.2 | [19.0;35.5] | 167.9 | <0.01 |
| 37 | Virton | 27.6 | [19.3;35.9] | 169.3 | <0.01 |
| 38 | Thuin | 28.6 | [23.6;33.5] | 177.7 | <0.001 |
| 39 | Neufchateau | 34.2 | [25.8;42.7] | 218.6 | <0.001 |
| 40 | Philippeville | 34.8 | [26.5;43.2] | 214.1 | <0.001 |
| 41 | Ath | 38.0 | [30.3;45.7] | 234.0 | <0.001 |
| 42 | Dinant | 39.9 | [33.0;46.9] | 246.0 | <0.001 |
| 43 | Marche-en-Famenne | 42.0 | [32.0;51.9] | 259.4 | <0.001 |

  

# Mean Rate = 16.1

# 

# \* p value of the z statistic testing for a the difference between the arrondissement's rate and the mean rate
