# Supplementary material for: Mapping the cause-specific premature mortality reveals large between-districts disparity in Belgium, 2003–2009
Source: Arch Public Health. 2015 Mar 23;73(1):13. doi: 10.1186/s13690-015-0060-5 (PMC4412101; doi:10.1186/s13690-015-0060-5)
Supplement: Additional file 48: Table S23. — Road Accident Women 175. [file 13690_2015_60_MOESM48_ESM.zip › 13690_2015_60_MOESM48_ESM.html]

SAS Output


# Road Accident Premature Mortality in Women (1-74 yr), Belgium 2003-2009

# Ranking of the arrondissements by increased mortality

# Age-adjusted rates per 100.000

| Rank | ARROND | Age-adj.Rates | CI on age-adj.Rates | smr | p value\* |
| --- | --- | --- | --- | --- | --- |
| 1 | Brussels | 2.1 | [ 1.6; 2.6] | 44.4 | <0.001 |
| 2 | Halle-Vilvoorde | 2.7 | [ 2.0; 3.5] | 60.3 | <0.001 |
| 3 | Sint Niklaas | 2.8 | [ 1.5; 4.0] | 59.6 | <0.01 |
| 4 | Mechelen | 3.2 | [ 2.1; 4.3] | 70.0 | <0.05 |
| 5 | Mouscron | 3.2 | [ 0.8; 5.6] | 68.5 | ns. |
| 6 | Antwerpen | 3.3 | [ 2.6; 3.9] | 72.0 | <0.001 |
| 7 | Verviers | 3.4 | [ 2.1; 4.6] | 73.4 | ns. |
| 8 | Leuven | 3.4 | [ 2.5; 4.3] | 76.3 | <0.05 |
| 9 | Aalst | 3.4 | [ 2.2; 4.7] | 74.2 | ns. |
| 10 | Gent | 3.8 | [ 2.8; 4.7] | 81.7 | ns. |
| 11 | Dendermonde | 3.8 | [ 2.2; 5.3] | 83.1 | ns. |
| 12 | Oostende | 3.9 | [ 2.1; 5.6] | 92.2 | ns. |
| 13 | Brugge | 3.9 | [ 2.5; 5.3] | 79.4 | ns. |
| 14 | Kortrijk | 4.0 | [ 2.7; 5.4] | 89.1 | ns. |
| 15 | Arlon | 4.4 | [ 1.4; 7.5] | 100.9 | ns. |
| 16 | Turnhout | 4.7 | [ 3.5; 5.8] | 104.1 | ns. |
| 17 | Nivelles | 4.8 | [ 3.5; 6.0] | 104.2 | ns. |
| 18 | Li�ge | 5.2 | [ 4.1; 6.2] | 111.1 | ns. |
| 19 | Hasselt | 5.2 | [ 3.9; 6.4] | 112.7 | ns. |
| 20 | Ieper | 5.5 | [ 3.0; 8.0] | 127.3 | ns. |
| 21 | Charleroi | 5.6 | [ 4.4; 6.9] | 123.2 | ns. |
| 22 | Maaseik | 5.7 | [ 4.0; 7.5] | 126.0 | ns. |
| 23 | Mons | 5.7 | [ 4.0; 7.4] | 122.5 | ns. |
| 24 | Roeselare | 5.7 | [ 3.5; 8.0] | 126.0 | ns. |
| 25 | Soignies | 6.1 | [ 4.0; 8.1] | 134.2 | ns. |
| 26 | Huy | 6.2 | [ 3.5; 9.0] | 133.0 | ns. |
| 27 | Tongeren | 6.3 | [ 4.3; 8.4] | 133.6 | ns. |
| 28 | Oudenaarde | 6.4 | [ 3.8; 9.0] | 143.7 | ns. |
| 29 | Veurne | 6.6 | [ 2.7;10.5] | 137.9 | ns. |
| 30 | Namur | 6.8 | [ 5.1; 8.4] | 147.6 | <0.05 |
| 31 | Diksmuide | 6.8 | [ 2.6;11.0] | 145.1 | ns. |
| 32 | Thuin | 7.1 | [ 4.6; 9.5] | 149.2 | <0.05 |
| 33 | Waremme | 7.1 | [ 3.6;10.5] | 152.7 | ns. |
| 34 | Eeklo | 7.2 | [ 3.8;10.5] | 155.3 | ns. |
| 35 | Tielt | 7.3 | [ 4.1;10.6] | 157.8 | ns. |
| 36 | Bastogne | 8.2 | [ 3.3;13.1] | 179.2 | ns. |
| 37 | Dinant | 9.1 | [ 5.8;12.4] | 200.7 | <0.01 |
| 38 | Virton | 9.4 | [ 4.6;14.2] | 209.9 | <0.05 |
| 39 | Neufchateau | 9.7 | [ 5.1;14.3] | 206.2 | <0.05 |
| 40 | Ath | 10.1 | [ 6.2;14.0] | 221.6 | <0.01 |
| 41 | Tournai | 10.1 | [ 7.2;13.1] | 220.6 | <0.001 |
| 42 | Marche-en-Famenne | 12.9 | [ 7.3;18.5] | 278.5 | <0.01 |
| 43 | Philippeville | 13.8 | [ 8.6;19.0] | 293.0 | <0.001 |

  

# Mean Rate = 4.6

# 

# \* p value of the z statistic testing for a the difference between the arrondissement's rate and the mean rate
