# Supplementary material for: Mapping the cause-specific premature mortality reveals large between-districts disparity in Belgium, 2003–2009
Source: Arch Public Health. 2015 Mar 23;73(1):13. doi: 10.1186/s13690-015-0060-5 (PMC4412101; doi:10.1186/s13690-015-0060-5)
Supplement: Additional file 49: Table S24. — Non-Transport accident Men 175. [file 13690_2015_60_MOESM49_ESM.zip › 13690_2015_60_MOESM49_ESM.html]

SAS Output


# Non-Transport accident Premature Mortality in Men (1-74 yr), Belgium 2003-2009

# Ranking of the arrondissements by increased mortality

# Age-adjusted rates per 100.000

| Rank | ARROND | Age-adj.Rates | CI on age-adj.Rates | smr | p value\* |
| --- | --- | --- | --- | --- | --- |
| 1 | Maaseik | 8.3 | [ 6.2;10.4] | 52.7 | <0.001 |
| 2 | Leuven | 9.5 | [ 7.9;11.0] | 61.8 | <0.001 |
| 3 | Sint Niklaas | 9.8 | [ 7.5;12.0] | 61.5 | <0.001 |
| 4 | Hasselt | 10.3 | [ 8.5;12.0] | 66.9 | <0.001 |
| 5 | Diksmuide | 10.4 | [ 5.3;15.5] | 66.0 | ns. |
| 6 | Tongeren | 10.8 | [ 8.3;13.4] | 69.5 | <0.001 |
| 7 | Turnhout | 11.0 | [ 9.3;12.8] | 69.8 | <0.001 |
| 8 | Mechelen | 11.1 | [ 9.0;13.1] | 69.7 | <0.001 |
| 9 | Roeselare | 11.3 | [ 8.2;14.3] | 72.0 | <0.01 |
| 10 | Halle-Vilvoorde | 12.1 | [10.4;13.7] | 77.4 | <0.001 |
| 11 | Brugge | 12.4 | [10.1;14.6] | 80.5 | <0.01 |
| 12 | Waremme | 12.5 | [ 7.7;17.2] | 78.2 | ns. |
| 13 | Kortrijk | 12.5 | [10.2;14.9] | 80.4 | <0.05 |
| 14 | Antwerpen | 13.1 | [11.9;14.4] | 84.7 | <0.001 |
| 15 | Aalst | 13.6 | [11.2;16.0] | 89.5 | ns. |
| 16 | Dendermonde | 14.1 | [11.1;17.1] | 90.6 | ns. |
| 17 | Eeklo | 14.2 | [ 9.6;18.9] | 88.4 | ns. |
| 18 | Veurne | 14.4 | [ 8.9;20.0] | 86.4 | ns. |
| 19 | Gent | 14.5 | [12.6;16.3] | 93.9 | ns. |
| 20 | Ieper | 14.7 | [10.6;18.7] | 96.6 | ns. |
| 21 | Tielt | 14.8 | [10.3;19.3] | 94.0 | ns. |
| 22 | Nivelles | 16.3 | [13.9;18.7] | 102.9 | ns. |
| 23 | Verviers | 16.7 | [13.9;19.5] | 106.5 | ns. |
| 24 | Brussels | 18.1 | [16.5;19.6] | 116.0 | <0.01 |
| 25 | Tournai | 18.1 | [14.1;22.1] | 121.6 | ns. |
| 26 | Oudenaarde | 19.0 | [14.5;23.4] | 122.5 | ns. |
| 27 | Oostende | 20.0 | [16.0;24.0] | 125.3 | <0.05 |
| 28 | Virton | 20.3 | [12.8;27.7] | 125.0 | ns. |
| 29 | Mouscron | 20.5 | [14.5;26.6] | 134.9 | ns. |
| 30 | Arlon | 20.6 | [13.5;27.6] | 128.4 | ns. |
| 31 | Thuin | 20.9 | [16.6;25.1] | 134.9 | <0.05 |
| 32 | Namur | 21.0 | [17.9;24.1] | 132.8 | <0.001 |
| 33 | Mons | 21.6 | [18.2;25.0] | 138.1 | <0.001 |
| 34 | Huy | 21.9 | [16.8;27.1] | 145.7 | <0.05 |
| 35 | Marche-en-Famenne | 22.5 | [15.0;30.0] | 139.6 | ns. |
| 36 | Li�ge | 22.5 | [20.3;24.7] | 145.1 | <0.001 |
| 37 | Soignies | 23.2 | [19.1;27.3] | 149.5 | <0.001 |
| 38 | Charleroi | 23.2 | [20.5;25.9] | 148.6 | <0.001 |
| 39 | Philippeville | 23.7 | [17.0;30.4] | 158.4 | <0.05 |
| 40 | Ath | 24.3 | [18.2;30.4] | 160.1 | <0.01 |
| 41 | Neufchateau | 25.2 | [17.7;32.7] | 161.9 | <0.05 |
| 42 | Dinant | 25.5 | [20.0;31.0] | 167.2 | <0.001 |
| 43 | Bastogne | 30.5 | [21.1;39.9] | 207.0 | <0.01 |

  

# Mean Rate = 15.5

# 

# \* p value of the z statistic testing for a the difference between the arrondissement's rate and the mean rate
