# Supplementary material for: Mapping the cause-specific premature mortality reveals large between-districts disparity in Belgium, 2003–2009
Source: Arch Public Health. 2015 Mar 23;73(1):13. doi: 10.1186/s13690-015-0060-5 (PMC4412101; doi:10.1186/s13690-015-0060-5)
Supplement: Additional file 50: Table S25. — Non-Transport accident Women 175. [file 13690_2015_60_MOESM50_ESM.zip › 13690_2015_60_MOESM50_ESM.html]

SAS Output


# Non-Transport accident Premature Mortality in Women (1-74 yr), Belgium 2003-2009

# Ranking of the arrondissements by increased mortality

# Age-adjusted rates per 100.000

| Rank | ARROND | Age-adj.Rates | CI on age-adj.Rates | smr | p value\* |
| --- | --- | --- | --- | --- | --- |
| 1 | Eeklo | 2.9 | [ 0.9; 4.9] | 41.7 | <0.001 |
| 2 | Tongeren | 3.0 | [ 1.7; 4.4] | 44.6 | <0.001 |
| 3 | Hasselt | 3.4 | [ 2.4; 4.4] | 49.4 | <0.001 |
| 4 | Maaseik | 3.5 | [ 2.1; 4.9] | 51.2 | <0.001 |
| 5 | Tielt | 4.1 | [ 1.8; 6.4] | 59.9 | <0.05 |
| 6 | Turnhout | 4.4 | [ 3.3; 5.5] | 64.9 | <0.001 |
| 7 | Kortrijk | 4.6 | [ 3.2; 6.0] | 67.5 | <0.01 |
| 8 | Leuven | 4.9 | [ 3.8; 6.0] | 70.5 | <0.001 |
| 9 | Diksmuide | 4.9 | [ 1.5; 8.3] | 73.5 | ns. |
| 10 | Sint Niklaas | 4.9 | [ 3.4; 6.5] | 71.6 | <0.05 |
| 11 | Mechelen | 5.0 | [ 3.7; 6.4] | 73.7 | <0.01 |
| 12 | Oostende | 5.1 | [ 3.2; 7.1] | 71.6 | ns. |
| 13 | Halle-Vilvoorde | 5.2 | [ 4.2; 6.2] | 76.7 | <0.01 |
| 14 | Veurne | 5.3 | [ 2.4; 8.2] | 86.2 | ns. |
| 15 | Waremme | 5.4 | [ 2.3; 8.5] | 73.8 | ns. |
| 16 | Roeselare | 5.5 | [ 3.4; 7.5] | 80.9 | ns. |
| 17 | Aalst | 5.5 | [ 4.0; 7.1] | 81.1 | ns. |
| 18 | Brugge | 5.7 | [ 4.2; 7.2] | 80.3 | ns. |
| 19 | Dendermonde | 5.8 | [ 3.9; 7.7] | 83.1 | ns. |
| 20 | Oudenaarde | 5.8 | [ 3.4; 8.2] | 85.1 | ns. |
| 21 | Neufchateau | 5.9 | [ 2.2; 9.5] | 80.9 | ns. |
| 22 | Bastogne | 6.0 | [ 1.8;10.2] | 89.5 | ns. |
| 23 | Antwerpen | 6.2 | [ 5.3; 7.1] | 89.0 | ns. |
| 24 | Ieper | 6.7 | [ 4.0; 9.5] | 96.5 | ns. |
| 25 | Gent | 6.9 | [ 5.7; 8.2] | 100.0 | ns. |
| 26 | Verviers | 7.0 | [ 5.3; 8.8] | 101.9 | ns. |
| 27 | Virton | 7.2 | [ 2.9;11.5] | 103.0 | ns. |
| 28 | Marche-en-Famenne | 7.3 | [ 3.1;11.4] | 104.9 | ns. |
| 29 | Soignies | 7.4 | [ 5.2; 9.6] | 109.6 | ns. |
| 30 | Namur | 7.9 | [ 6.0; 9.7] | 112.5 | ns. |
| 31 | Ath | 8.1 | [ 4.7;11.6] | 119.2 | ns. |
| 32 | Brussels | 8.3 | [ 7.3; 9.4] | 121.0 | <0.01 |
| 33 | Dinant | 8.5 | [ 5.4;11.6] | 123.6 | ns. |
| 34 | Nivelles | 8.9 | [ 7.2;10.6] | 128.3 | <0.05 |
| 35 | Thuin | 9.2 | [ 6.5;11.9] | 135.6 | ns. |
| 36 | Tournai | 9.5 | [ 6.7;12.3] | 140.3 | ns. |
| 37 | Mouscron | 9.7 | [ 5.7;13.6] | 141.7 | ns. |
| 38 | Mons | 10.6 | [ 8.3;12.8] | 154.8 | <0.01 |
| 39 | Li�ge | 11.4 | [ 9.9;12.9] | 163.6 | <0.001 |
| 40 | Charleroi | 12.1 | [10.3;13.9] | 175.9 | <0.001 |
| 41 | Huy | 12.6 | [ 8.8;16.5] | 183.3 | <0.01 |
| 42 | Philippeville | 13.0 | [ 8.2;17.8] | 192.9 | <0.05 |
| 43 | Arlon | 13.8 | [ 8.3;19.4] | 206.5 | <0.05 |

  

# Mean Rate = 6.9

# 

# \* p value of the z statistic testing for a the difference between the arrondissement's rate and the mean rate
