# Supplementary material for: Mapping the cause-specific premature mortality reveals large between-districts disparity in Belgium, 2003–2009
Source: Arch Public Health. 2015 Mar 23;73(1):13. doi: 10.1186/s13690-015-0060-5 (PMC4412101; doi:10.1186/s13690-015-0060-5)
Supplement: Additional file 51: Table S26. — Suicide Men 175. [file 13690_2015_60_MOESM51_ESM.zip › 13690_2015_60_MOESM51_ESM.html]

SAS Output


# Suicide Premature Mortality in Men (1-74 yr), Belgium 2003-2009

# Ranking of the arrondissements by increased mortality

# Age-adjusted rates per 100.000

| Rank | ARROND | Age-adj.Rates | CI on age-adj.Rates | smr | p value\* |
| --- | --- | --- | --- | --- | --- |
| 1 | Maaseik | 19.2 | [16.1;22.4] | 72.0 | <0.001 |
| 2 | Mechelen | 20.2 | [17.5;23.0] | 76.9 | <0.001 |
| 3 | Leuven | 20.4 | [18.1;22.7] | 77.3 | <0.001 |
| 4 | Hasselt | 20.6 | [18.2;23.1] | 77.6 | <0.001 |
| 5 | Antwerpen | 20.9 | [19.2;22.5] | 78.5 | <0.001 |
| 6 | Brussels | 21.0 | [19.4;22.6] | 78.5 | <0.001 |
| 7 | Tongeren | 21.3 | [17.7;24.8] | 81.2 | <0.01 |
| 8 | Halle-Vilvoorde | 21.5 | [19.3;23.6] | 81.7 | <0.001 |
| 9 | Turnhout | 21.8 | [19.3;24.2] | 82.1 | <0.001 |
| 10 | Diksmuide | 23.4 | [15.8;30.9] | 89.4 | ns. |
| 11 | Sint Niklaas | 25.1 | [21.5;28.7] | 93.4 | ns. |
| 12 | Gent | 25.3 | [22.9;27.8] | 95.5 | ns. |
| 13 | Nivelles | 26.2 | [23.2;29.2] | 100.1 | ns. |
| 14 | Ieper | 26.2 | [20.7;31.7] | 100.0 | ns. |
| 15 | Tielt | 26.3 | [20.3;32.3] | 98.4 | ns. |
| 16 | Brugge | 27.5 | [24.0;31.0] | 103.7 | ns. |
| 17 | Bastogne | 28.7 | [19.6;37.8] | 110.3 | ns. |
| 18 | Oudenaarde | 28.8 | [23.4;34.3] | 109.0 | ns. |
| 19 | Aalst | 28.9 | [25.3;32.5] | 107.4 | ns. |
| 20 | Soignies | 29.0 | [24.5;33.5] | 111.1 | ns. |
| 21 | Kortrijk | 29.0 | [25.5;32.6] | 110.2 | ns. |
| 22 | Roeselare | 29.1 | [24.1;34.0] | 109.0 | ns. |
| 23 | Veurne | 29.1 | [21.3;36.9] | 113.7 | ns. |
| 24 | Verviers | 29.9 | [26.2;33.5] | 114.2 | ns. |
| 25 | Charleroi | 30.1 | [27.1;33.1] | 113.8 | <0.05 |
| 26 | Arlon | 30.4 | [22.3;38.6] | 117.1 | ns. |
| 27 | Dendermonde | 30.4 | [26.1;34.8] | 115.0 | ns. |
| 28 | Mouscron | 31.0 | [23.6;38.4] | 120.7 | ns. |
| 29 | Oostende | 31.8 | [26.7;36.9] | 120.3 | <0.05 |
| 30 | Mons | 32.1 | [28.0;36.1] | 121.5 | <0.01 |
| 31 | Thuin | 32.6 | [27.3;37.8] | 124.0 | <0.05 |
| 32 | Philippeville | 33.1 | [25.1;41.1] | 125.8 | ns. |
| 33 | Li�ge | 33.5 | [30.9;36.1] | 127.0 | <0.001 |
| 34 | Tournai | 34.2 | [28.7;39.7] | 128.7 | <0.01 |
| 35 | Namur | 36.2 | [32.3;40.1] | 138.3 | <0.001 |
| 36 | Eeklo | 36.5 | [29.1;43.8] | 135.6 | <0.01 |
| 37 | Marche-en-Famenne | 36.6 | [27.3;45.9] | 137.0 | <0.05 |
| 38 | Waremme | 37.9 | [29.8;45.9] | 143.2 | <0.01 |
| 39 | Ath | 38.1 | [30.4;45.7] | 143.0 | <0.01 |
| 40 | Neufchateau | 43.3 | [33.6;52.9] | 165.3 | <0.001 |
| 41 | Huy | 43.9 | [36.6;51.1] | 165.4 | <0.001 |
| 42 | Virton | 45.1 | [34.5;55.8] | 172.2 | <0.001 |
| 43 | Dinant | 46.5 | [38.9;54.0] | 173.4 | <0.001 |

  

# Mean Rate = 26.4

# 

# \* p value of the z statistic testing for a the difference between the arrondissement's rate and the mean rate
