# Supplementary material for: Mapping the cause-specific premature mortality reveals large between-districts disparity in Belgium, 2003–2009
Source: Arch Public Health. 2015 Mar 23;73(1):13. doi: 10.1186/s13690-015-0060-5 (PMC4412101; doi:10.1186/s13690-015-0060-5)
Supplement: Additional file 52: Table S27. — Suicide Women 175. [file 13690_2015_60_MOESM52_ESM.zip › 13690_2015_60_MOESM52_ESM.html]

SAS Output


# Suicide Premature Mortality in Women (1-74 yr), Belgium 2003-2009

# Ranking of the arrondissements by increased mortality

# Age-adjusted rates per 100.000

| Rank | ARROND | Age-adj.Rates | CI on age-adj.Rates | smr | p value\* |
| --- | --- | --- | --- | --- | --- |
| 1 | Maaseik | 5.7 | [ 4.0; 7.4] | 60.3 | <0.001 |
| 2 | Neufchateau | 6.8 | [ 2.9;10.6] | 69.7 | ns. |
| 3 | Sint Niklaas | 6.9 | [ 5.0; 8.7] | 71.3 | <0.01 |
| 4 | Soignies | 7.0 | [ 4.9; 9.2] | 72.5 | <0.05 |
| 5 | Arlon | 7.5 | [ 3.4;11.5] | 77.9 | ns. |
| 6 | Nivelles | 7.7 | [ 6.1; 9.3] | 80.1 | <0.05 |
| 7 | Mechelen | 7.9 | [ 6.2; 9.6] | 81.8 | <0.05 |
| 8 | Antwerpen | 8.0 | [ 7.0; 9.0] | 81.7 | <0.01 |
| 9 | Leuven | 8.1 | [ 6.6; 9.5] | 83.3 | <0.05 |
| 10 | Turnhout | 8.1 | [ 6.6; 9.6] | 83.4 | <0.05 |
| 11 | Thuin | 8.2 | [ 5.6;10.8] | 85.3 | ns. |
| 12 | Halle-Vilvoorde | 8.2 | [ 6.9; 9.5] | 83.6 | <0.05 |
| 13 | Tongeren | 8.3 | [ 6.0;10.5] | 83.9 | ns. |
| 14 | Dendermonde | 8.4 | [ 6.2;10.7] | 87.2 | ns. |
| 15 | Oudenaarde | 8.6 | [ 5.7;11.6] | 91.2 | ns. |
| 16 | Hasselt | 8.8 | [ 7.2;10.4] | 91.3 | ns. |
| 17 | Mons | 9.1 | [ 7.1;11.2] | 95.3 | ns. |
| 18 | Charleroi | 9.3 | [ 7.7;11.0] | 95.3 | ns. |
| 19 | Veurne | 9.3 | [ 5.1;13.6] | 101.7 | ns. |
| 20 | Bastogne | 9.6 | [ 4.4;14.9] | 103.0 | ns. |
| 21 | Mouscron | 9.7 | [ 5.6;13.8] | 100.4 | ns. |
| 22 | Tournai | 9.9 | [ 7.0;12.8] | 101.9 | ns. |
| 23 | Verviers | 9.9 | [ 7.8;12.0] | 101.6 | ns. |
| 24 | Ieper | 10.0 | [ 6.7;13.4] | 106.8 | ns. |
| 25 | Kortrijk | 10.2 | [ 8.1;12.3] | 106.5 | ns. |
| 26 | Brussels | 10.3 | [ 9.2;11.4] | 105.1 | ns. |
| 27 | Aalst | 10.6 | [ 8.4;12.7] | 106.4 | ns. |
| 28 | Ath | 10.9 | [ 6.9;14.9] | 113.5 | ns. |
| 29 | Gent | 11.1 | [ 9.5;12.7] | 113.3 | ns. |
| 30 | Waremme | 11.4 | [ 7.1;15.7] | 117.6 | ns. |
| 31 | Tielt | 11.6 | [ 7.6;15.5] | 121.6 | ns. |
| 32 | Philippeville | 11.7 | [ 7.1;16.3] | 125.2 | ns. |
| 33 | Namur | 11.9 | [ 9.6;14.1] | 120.4 | ns. |
| 34 | Virton | 12.1 | [ 6.6;17.5] | 127.2 | ns. |
| 35 | Marche-en-Famenne | 12.3 | [ 6.9;17.8] | 125.1 | ns. |
| 36 | Diksmuide | 12.4 | [ 6.8;18.0] | 129.3 | ns. |
| 37 | Brugge | 12.6 | [10.3;14.9] | 130.5 | <0.05 |
| 38 | Oostende | 12.7 | [ 9.5;15.8] | 133.7 | ns. |
| 39 | Huy | 13.0 | [ 9.1;16.8] | 134.8 | ns. |
| 40 | Eeklo | 13.7 | [ 9.2;18.3] | 141.5 | ns. |
| 41 | Dinant | 13.9 | [ 9.9;18.0] | 144.2 | <0.05 |
| 42 | Li�ge | 14.5 | [12.8;16.2] | 149.7 | <0.001 |
| 43 | Roeselare | 14.6 | [11.1;18.2] | 148.9 | <0.01 |

  

# Mean Rate = 9.7

# 

# \* p value of the z statistic testing for a the difference between the arrondissement's rate and the mean rate
